# Virus-like particles displaying conserved toxin epitopes stimulate polyspecific, murine antibody responses capable of snake venom recognition

**DOI:** 10.1038/s41598-022-13376-x

**Published:** 2022-07-05

**Authors:** Stefanie K. Menzies, Charlotte A. Dawson, Edouard Crittenden, Rebecca J. Edge, Steven R. Hall, Jaffer Alsolaiss, Mark C. Wilkinson, Nicholas R. Casewell, Robert A. Harrison, Stuart Ainsworth

**Affiliations:** 1 Centre for Snakebite Research and Interventions, Liverpool School of Tropical Medicine, Pembroke Place, Liverpool, L3 5QA UK; 2 Centre for Drugs and Diagnostics, Liverpool School of Tropical Medicine, Pembroke Place, Liverpool, L3 5QA UK

**Keywords:** Biological techniques, Biotechnology, Computational biology and bioinformatics, Immunology, Molecular medicine

## Abstract

Antivenom is currently the first-choice treatment for snakebite envenoming. However, only a low proportion of antivenom immunoglobulins are specific to venom toxins, resulting in poor dose efficacy and potency. We sought to investigate whether linear venom epitopes displayed on virus like particles can stimulate an antibody response capable of recognising venom toxins from diverse medically important species. Bioinformatically-designed epitopes, corresponding to predicted conserved regions of group I phospholipase A_2_ and three finger toxins, were engineered for display on the surface of hepatitis B core antigen virus like particles and used to immunise female CD1 mice over a 14 weeks. Antibody responses to all venom epitope virus like particles were detectable by ELISA by the end of the immunisation period, although total antibody and epitope specific antibody titres were variable against the different epitope immunogens. Immunoblots using pooled sera demonstrated recognition of various venom components in a diverse panel of six elapid venoms, representing three continents and four genera. Insufficient antibody yields precluded a thorough assessment of the neutralising ability of the generated antibodies, however we were able to test polyclonal anti-PLA_2_ IgG from three animals against the PLA﻿_2_ activity of *Naja nigricollis* venom, all of which showed no neutralising ability. This study demonstrates proof-of-principle that virus like particles engineered to display conserved toxin linear epitopes can elicit specific antibody responses in mice which are able to recognise a geographically broad range of elapid venoms.

## Introduction

Snakebite envenoming (SBE) is a Neglected Tropical Disease estimated to result in a yearly burden of 138,000 deaths and 400,000 disabilities^1^, and which disproportionately affects the mostly impoverished, rural-dwelling and marginalised communities of the tropics and sub-tropics^1,2^. A key driver of the persisting high SBE mortality and morbidity rate is the lack of safe and effective therapies, coupled to the inaccessibility (both physical and financial) of healthcare^3–6^.

SBE is caused by a diverse number of snake species and a wide variety of pathologies can be observed post envenoming^1^. However, in very broad terms, three main envenoming syndromes are observed globally: haemotoxic envenoming which disrupts haemostasis, neurotoxic envenoming which causes rapid descending neuromuscular paralysis, and envenoming which results in local tissue destruction and often necrosis^1^. Further systemic effects of snake envenoming may include nephrotoxicity and myotoxicity, depending upon the biting species^1^. Currently the only specific therapy for envenoming is antivenom, which consists of antibodies isolated from animals hyper-immunised with crude venom(s)^7^. Antivenom effectiveness is highly variable in neutralising different envenoming pathologies, however. Generally, if an appropriate antivenom is used (i.e. an antivenom indicated for the biting snake species), it is effective at restoring regular systemic haemostasis during severe haemotoxic envenoming^8^, even with substantial delay in administration. However, the extent of antivenom effectiveness in the management of neurotoxic and local envenoming effects remains contested^9–12^. Studies of the pharmacodynamic properties of individual, toxin specific antibodies are likely able to address why antivenoms are more effective against some envenoming pathologies than others^13–15^. Such properties include their structural format (e.g. intact immunoglobulin or fragments such as F(ab’)_2_ and Fab), their specificity and affinity for different toxins with targets in multiple tissue components and the initiation of ultimately detrimental host processes (such as potential proinflammatory cascades) coupled with times to treatment delivery.

Antivenoms are manufactured using high-cost, century-old protocols of immunising horses or sheep with crude venom(s) which comprise ~ 20–100 components. This unguided approach, whilst clearly able to generate lifesaving therapeutics, has limitations in that it does not consider (1) the diverse range of envenoming pathologies, (2) distinct venom protein immunogenicities or (3) distinct toxicities of different venom components^1,16–19^. This is problematic as snake venoms and their toxins vary at every taxonomic level^20^ and some of the most pathogenic and diverse toxin families, particularly those responsible for neurotoxic and cytotoxic pathologies in elapids, are frequently described as poorly immunogenic^21,22^. Consequently, antivenoms are snake-species and geographically restricted and are of very low specificity and potency, with only ~ 15% of antivenom antibodies binding to the venom proteins used as immunogens^23^ resulting in poor clinical dose efficacy, particularly for neurotoxic snake envenoming^21^. As a result of these limitations and the effect they have on treating snakebite globally, the World Health Organization and other organisations such as Wellcome, have recently advocated the development of improved antivenom manufacturing and new envenoming therapies to overcome these deficiencies and ultimately reduce mortality and morbidity^24,25^.

Research over the past two decades has increasingly focussed on overcoming these shortcomings through rationalised selection and design of immunogens to improve the dose and polyspecific efficacy of antivenom^18,25–28^. Facilitated through the increase in accessibility of venom transcriptomes and proteomes^29^, we and several groups have sought to rationalise/tailor immunising mixtures to focus the immune response solely towards medically important and/or cross-species conserved toxins, with the hopes of increasing snake species coverage and dose efficacy. Several approaches have been used with promising results including rational selection of key purified toxins^30,31^, cDNA immunisation of vectors encoding whole toxins^18,32^, use of multi-antigenic peptides and construction of synthetic consensus toxins^33^ and the use of recombinant conserved venom-epitope immunogens^27,34–36^. The latter approach involves producing recombinant molecules, either as DNA or peptide immunogens, which encode linear venom epitopes. Immunisation of animals with these immunogens has demonstrated the production of antibodies capable of cross-generically neutralising venom-induced pathology^27,34^. However, peptide immunogens show variable, unpredictable and, frequently, poor immunogenicity, are often difficult to recombinantly produce and are expensive to chemically synthesise^37^, limiting their application for generating a rationally designed antivenom. One way to potentially overcome these limitations is to genetically fuse peptide immunogens to virus-like particles (VLPs).

VLPs consist of a structural component of a virus that spontaneously self-assembles into a large complex similar in size and shape to its parent virion^38^. As VLPs do not contain any genetic material, they are non-infectious and are unable to replicate^39^. However, due to their size and structural similarity to functional viruses, VLPs are highly immunostimulatory^39,40^. For instance, VLPs can cross link T cell independent and dependent B cell responses to elicit neutralising immune responses without need for adjuvants. Due to their ability to drive strong immune responses, VLPs have been used in vaccine research for 40 years, with several approved for human VLP based vaccines^38,41^. A highly characterised VLP is the Hepatitis B core antigen (HBcAg)^42,43^. When expressed in *E. coli*, HBcAg monomers spontaneously self-assemble into homodimers which subsequently assemble into VLPs consisting of 240 HBcAg monomers. HBcAg can be engineered to harbour heterologous antigens which are prominently displayed in key immunodominant sites when expressed on the VLP surface^43^. Furthermore, immunisation of animals using VLPs displaying heterologous antigens of various pathogens has been demonstrated to elicit protective antibody responses^44–46^, however, the potential of the VLP-approach to generate serotherapies has yet to be investigated.

Here, we sought to investigate whether the characteristics of HBcAg VLPs are suitable for production and display of rationally selected and engineered cross-generic consensus epitopes of three finger toxins (3FTX) and Group I phospholipase A_2_ (PLA_2_) from African and Asian elapid snakes; with the overarching aim of utilising this approach to facilitate the monoclonal antibody discovery and development objectives of the Scientific Partnership for Neglected Tropical Snakebite (SRPNTS; www.srpnts.org). Our results suggest that this approach can elicit immunological responses capable of generating antibodies that cross-generically recognise venom components from snakes inhabiting pan-continental locations.

## Results

### Selection of epitopes

Our strategy for assessing the immunostimulatory potential of VLPs coated with venom antigens started with selection of the most conserved and polyspecifically-representative 3FTX and group I PLA_2_ toxin epitopes, as described in the Supplemental Materials and Methods. The 264 3FTX sequences were assigned to 21 individual homology groups (GR1 through GR21) (Table 1, Supp. Table S2). These homology groups broadly corresponded to functional sub-family annotation, although notable exceptions were observed. For example, type I alpha-neurotoxins (sNTX) splitting into two distinct groups: GR17 & GR15, while GR7 consisted of type IA cytotoxins (CTX) and Orphan group XV (OGXV) (Supp. Table S3).

**Table 1:** Epitopes selected for immunisation.

Name	Homology Group	Toxin type	Peptide sequence	Predicted toxin target host species*	Immun. group
CTX_C	GR7	Cytotoxic 3FTX	TCPEGKNL	*N. annulifera*, *N. mossambica*, *N. naja*, *N. nigricollis*, *N. nivea*, *N. nubiae*, *N. oxiana*, *N. pallida*, *N. philippinensis*	A/M
CTX_F	GR7	Cytotoxic 3FTX	IDVCPKSSLL	*N. atra*, *N. melanoleuca*, *N. mossambica*, *N. nigricollis*, *N. nubiae*, *N. oxiana*, *N. pallida*, *N. philippinensis*, *N. siamensis*, *N. sumatrana*	B
ATX_C	GR1	Aminergic-type 3FTX	DCPDGQNLC	*D. angusticeps*, *D. jamesoni kaimosae*, *D. polylepis*, *D. viridis*, *N. naja*, *N. nigricollis*, *N. nivea*, *N. nubiae*, *N. pallida*, *W. aegyptia*	C
ATX_F	GR1	Aminergic-type 3FTX	TRGCAATCP	*A. scutatus*, *D. angusticeps*, *D. polylepis*, *H. haemachatus*, *N. haje*, *N. kaouthia*, *N. melanoleuca*, *N. mossambica*, *N. naja*, *N. nigricollis*, *N. nivea*, *N. nubiae*, *N. pallida*, *N. siamensis*	D
sNTX_C	GR17	Short type I α 3FTX	CHNQQSSQ	*A. scutatus*, *H. haemachatus*, *N. atra*, *N. kaouthia*, *N. naja*, *N. nivea*, *N. oxiana*, *N. pallida*, *N. philippinensis*, *N. siamensis*, *W. aegyptia*	E
sNTX_F1	GR17	Short type I α 3FTX	DHRGTIIE	*D. jamesoni kaimosae*, *D. jamesoni jamesoni*, *D. polylepis*, *D. viridis*, *H. haemachatus*, *N. atra*, *N. haje*, *N. nivea*, *N. oxiana*, *N. pallida*, *N. philippinensis*, *N. siamensis*	F
sNTX_F2	GR17	Short type I α 3FTX	DHRGYRTE	*N. atra*, *N. kaouthia*, *N. naja*, *N. nigricollis*, *N. siamensis*	G
PLA2_1	–	Group I PLA_2_	KGTPVDDLD	*H. haemachatus*, *N. haje*, *N. melanoleuca*, *N. mossambica*, *N. nigricollis*, *N. nivea*, *N. pallida*	H
PLA2_2	–	Group I PLA_2_	KGTAVDDLD	*H. haemachatus*, *N. mossambica*, *N. nigricollis*, *N. nubiae*, *N. pallida*, *W. aegyptia*	I
PLA2_3	–	Group I PLA_2_	SGTPVDDLD	*H. haemachatus*, *N. atra*, *N. kaouthia*, *N. melanoleuca*, *N. mossambica*, *N. naja*, *N. nigricollis*, *N. nubiae*, *N. pallida*, *N. philippinensis*, *N. siamensis*, *N. sumatrana*, *W. aegyptia*	J
Core string	–	3FTX string (core epitopes)	KKDCPDGQNLCKKCAKTCTEEKKGCTFSCPEKKGCTFTCPEKKTKSCEENSKKTTSCGDYFKKCHNQQSSQKKTCPEGKNL	*D. angusticeps*, *D. jamesoni jamesoni*, *D. jamesoni kaimosae*, *D. polylepis*, *D. viridis*, *H. hemachatus*, *N. annulifera*, *N. haje*, *N. kaouthia*, *N. melanoleuca*, *N. naja*, *N. nigricollis*, *N. nivea*, *N. nubiae*, *N. pallida*, *N. philippinensis*, *N. sumatrana*, *O. hannah*	K
Finger string	–	3FTX string (finger epitopes)	KKTPATTKSCKKDHRGTIIEKKDHRGYRTEKKIDVCPKSSLLKKTPETTEICPKKSGCHLKITKKTRGCAATCPKK	*A. scutatus*, *B. candidus*, *B. multicinctus*, *D. angusticeps*, *D. jamesoni jamesoni*, *D. jamesoni kaimosae*, *D. polylepis*, *D. viridis*, *H. haemachatus*, *M. fulvius*, *N. annulifera*, *N. haje*, *N. kaouthia*, *N. melanoleuca*, *N. naja*, *N. nigricollis*, *N. nivea*, *N. nubiae*, *N. oxiana*, *N. pallida*, *N. philippinensis*, *N. siamensis*, *N. sumatrana*	L

* = species with 100% amino acid toxin-epitope matches.

Of the 21 3FTX groups, six (aminergic-type [ATX] GR1, CTX and OGXV GR7, non-conventional [NCX] GR10, orphan group VIII [OG8] GR13, sNTX GR15 and sNTX GR17) were selected based on their represented frequency in the data set (Supp. Table S2). Hereafter, these groups will be referred to by their toxin-type only (e.g. ATX, CTX, OGXV, NCX, OG8, or SNTX) (Table 1). Combined, these six groups represented 70.6% (187/264) of the 3FTX sequences analysed in the study. Sequence conservation within 3FTX groups (except for NCX) was generally high, with 49–66% of AA residues being ≥ 80% conserved across all group sequences (Supp. Figs. S2 and S3). Group I PLA_2_ sequences were very homogenous (68% and 48% AA residues at 80% and 100% conservation) across full-length sequences (Supp. Fig. S4).

Fifteen individual 3FTX epitopes were designed based on (1) BepiPred predicted epitope regions, (2) conservation within groups, (3) predicted accessibility and (4) their molecular location: either the hydrophobic core or 1st or 3rd finger of 3FTX, designated by “_F” or “_C”, respectively (Supp. Figs. S2, S3, S5, Supp. Table S3, Supp. File S5). It was not always possible to design epitopes corresponding to regions which reflected predicted epitopes due to limited sequence conservation in predicted regions. In such cases epitopes were solely designed on sequence conservation and accessibility (e.g., ATX_F, NCX_F, OG8_C [Supp. Figs. S2, S3, S5]). Epitope ATX_F was near identical (1 AA greater in length) to epitope Pep604-B designed to elicit antibodies against *Micrurus corallinus* 3FTX^35^.

Three variants of a single group I PLA_2_ epitope were designed (Table 1) based on conservation and accessibility (Supp. Figs. S4, S6). Cross-referencing this epitope region with publicly available Snake Toxin and Antivenom Binding profiles^47^ suggests this region is readily recognised by current antivenoms. The selected PLA_2_ epitope region resides between the calcium binding loop (Tyr 28, Gly 30, Gly 32) and Asp 49 (Supp. Figs. S4, S6) residues essential for calcium ion positioning for hydrolytic activity^48^.

### Expression of VLPs presenting snake venom 3FTX and PLA_2_ epitopes

A sub-selection of individual designed epitopes were chosen for expression on VLPs, herein referred to as venom epitope VLPs (veVLPs), and were expressed and purified as described in the Supplemental Materials and Methods. Despite the resulting recombinant veVLPs expressed in *Escherichia coli* proving to be largely insoluble, sufficient quantities of soluble material were obtained for each veVLP. Attempts to improve solubility by varying incubation temperature and inducing IPTG concentration resulted in little-to-no improvement. The methods detailed for the expression of veVLPs resulted in a mean yield of 9.97 mg/L soluble veVLP from a 0.6 L culture (range 1.7–16.4 mg/L). To ensure confidence in the assembly of veVLPs (as opposed to monomers resulting from non-assembly), all affinity-purified veVLPs were concentrated with a 100 kDa MWCO centrifugal filter to deplete any sub-100 kDa proteins whilst retaining assembled veVLPs^49^. All purified veVLPs were visually assessed for purity using anti-His fluorescent immuno-blots, comparing total protein stain (Supp. Fig. S7A) to anti-His signal (Supp. Fig. S7B). As shown in Supp. Fig. S7, the major bands in purified samples contained His-tagged proteins corresponding to the individual expected molecular weight of the reduced monomeric constituent proteins of each veVLP monomer. Larger bands (presumably) corresponding to dimeric complexes of individual monomers were also visible for the majority of veVLPs. The veVLPs were subsequently probed with SAIMR Polyvalent antivenom to determine if antibodies raised against crude venom could recognise venom epitopes displayed on VLPs. Results demonstrated antivenom recognition of four veVLPs—sNTX_F1 and sNTX_F2, 3FTX Core-string and 3FTX Finger-string. No recognition of the other veVLPs by SAIMR Polyvalent was apparent (Supp. Fig. S7D).

### VLPs presenting snake venom epitopes induce antibody responses in mice

Female CD1 mice were immunised with different veVLPs over a 14-week immunisation schedule (see “Materials and methods” section). We were required to humanely euthanise 20 individuals in-line with our experimental licence (Table 2). This severely restricted monitoring of responses in several immunogen groups.

**Table 2:** Summary of individual animal sera recognition of veVLPs and venoms at week 14.

Group	veVLP epitope	Toxin type	Adj.	Analysis	Individual				
					1	2	3	4	5
A	CTX_C	Cytotoxic 3FTx	YES	Blot-versus venom	†	†	†	0	0
				ELISA versus veVLP (OD_405_)				0.70	0.67
B	CTX_F	Cytotoxic 3FTx	YES	Blot-versus venom	†	†	†	†	0
				ELISA versus veVLP (OD_405_)					0.41
C	ATX_C	Aminergic-type 3FTx	YES	Blot-versus venom	0	0	0	†	Dp, Nk, Ns,
				ELISA versus veVLP (OD_405_)	1.95	1.93	1.98		1.82
D	ATX_F	Aminergic-type 3FTx	YES	Blot-versus venom	Bc, Dp, Nk, Ns, Nn	†	0	†	Bc, Dp, Nk, Ns, Nn
				ELISA versus veVLP (OD_405_)	1.87		1.62		1.99
E	sNTX_C	Short type I α 3FTx	YES	Blot-versus venom	0	0	†	†	†
				ELISA versus veVLP (OD_405_)	0.55	0.58			
F	sNTX_F1	Short type I α 3FTx	YES	Blot-versus venom	0	0	Ns	†	Dp, Nk, Ns
				ELISA versus veVLP (OD_405_)	1.33	1.99	1.9		0.33
G	sNTX_F2	Short type I α 3FTx	YES	Blot-versus venom	0	0	0	0	Bc
				ELISA versus veVLP (OD_405_)	1.81	2.14	2.05	0.93	1.99
H	PLA2_1	Group I PLA_2_	YES	Blot-versus venom	Nk, Ns, Nn, Os	†	Nn, Os	†	Nk, Ns, Nn, Os
				ELISA versus veVLP (OD_405_)	1.93		1.94		1.95
I	PLA2_2	Group I PLA_2_	YES	Blot-versus venom	0	0	†	0	0
				ELISA versus veVLP (OD_405_)	1.99	1.88		2.01	1.8
J	PLA2_3	Group I PLA_2_	YES	Blot-versus venom	†	Ns, Nk, Nn, Os	0	0	†
				ELISA versus veVLP (OD_405_)		1.87	1.87	1.59	
K	Core string	3FTx string (core epitopes)	YES	Blot-versus venom	0	Bc, Dp, Nk, Ns	0	0	Nk, Nn, Os
				ELISA versus veVLP (OD_405_)	0.21	2.1	1.1	1.62	1.02
L	Finger string	3FTx string (finger epitopes)	YES	Blot-versus venom	Os	Os	Ns	0	†
				ELISA versus veVLP (OD_405_)	1.79	1.54	2.23	2.04	
M	CTx_C	Cytotoxic 3FTx	NO	Blot-versus venom	Os	Os	Nn, Os	†	Os
				ELISA versus veVLP (OD_405_)	1.02	0.21	0.6		1.58

Sera from individuals that were euthanised before the end of the experiment were unable to be analysed, as represented by the symbol †.

Blot-versus venom rows denotes the venoms recognised by each animal at week 14 via immuno-blot (visual inspection of blots in File S6).

**Figure 1:**
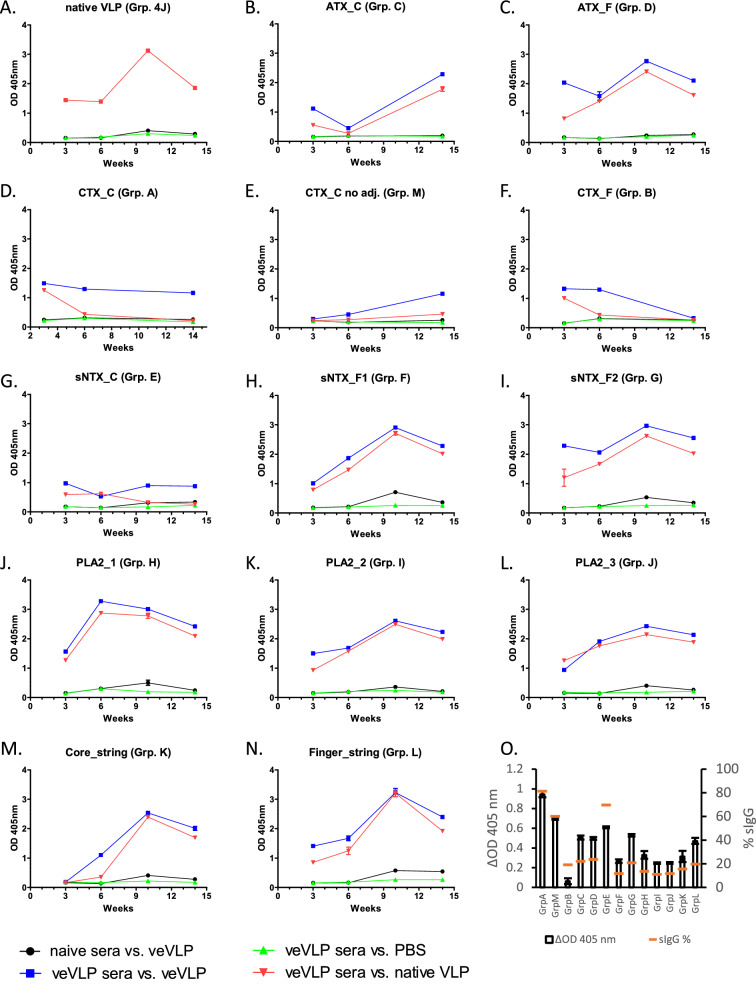
Panels (**A**–**N**) ELISA time course of antibody responses to immunisation with veVLP antigens. OD_405nm_ values displayed are signals generated using a 1 in 500 dilution of neat sera pooled from all individuals in that group. *ATX* Aminergic-type, *CTX* type 1A cytotoxin, *sNTX* short chain neurotoxin, *PLA2* group I phospholipase A_2_, _*C* core located epitope, _*F* finger located epitope. Panel (**O**) Demonstration of proportion of pooled group terminal sera at 1 in 500 dilution, in recognising respective specific epitope versus the VLP, displayed as both difference in OD_405_ (ΔOD405) between veVLP and native VLP, and as a percentage proportion of the total IgG response (specific IgG [sIgG]). Results represent triplicate readings, with exceptions stated in Supp. File 4. Error bars represent ± standard deviation.

*Bc* *Bungarus candidus*, *Dp* *Dendroapsis polylepis*, *Nk* *Naja kaouthia*, *Ns* *Naja subfulva*, *Os* *Oxyuranus scutellatus,* *0* no venoms recognised. ELISA versus veVLP displays absorbance values of the mean 1/500 OD_405_ reading of each individual’s sera at week 14 (see Fig. 1 for more details). *Adj.* adjuvant.

The antibody response of mice to veVLP immunisation was monitored at specific points (at weeks 3, 6, 10 and at the end of experiment at week 14) via ELISA using pooled sera consisting of equal volumes from each individual in each experimental group (Fig. 1, Supp. File S4). Antibody responses to veVLPs were detected at week 3 for all groups (OD_405_ ≥ 1 at 1/500 dilution of sera), with the exception of mice receiving Core-string (Group K) and CTX_C (Group M—without adjuvant) veVLP immunogens, whose signal was indistinguishable to that of naïve serum or negative controls. The OD_405_ for 7 of the 13 veVLP groups continued to increase until reaching a peak at week 10, which then subsequently declined modestly by week 14. Due to a processing error, we unfortunately lost week 10 sera corresponding to animals immunised with CTX_C (Group A—with adjuvant), CTX_C (Group M without adjuvant) and CTX_F (Group B), thus it is not possible to infer if a similar response profile occurred with CTX_C or CTX_F veVLPs.

CTX_C , CTX_F and sNTX veVLP immunised groups (A/M, B and E) provided the lowest overall titres at terminal bleed (week 14) (Fig. 1). Both CTX_C immunised groups (groups A and M with and without adjuvant, respectively) resulted in near identical mean titres (1/500 OD_405_ 1.16 and 1.15, respectively), however, CTX_C with adjuvant (Group A) titres remained stable throughout the schedule, whereas CTX_C without adjuvant (Group M) titres slowly reached this titre by week 14. CTX_F (Group B) titres declined after week 6, possibly reflective of the early euthanasia of 80% of the individuals in this group on humane grounds (Table 2), thus this data only reflects n = 1 from week 6 onwards. sNTX_C (Group E) titres remained stable but relatively low throughout the immunisation period (maximum mean 1/500 OD_405_ = 0.97).

### VLPs presenting snake venom epitopes elicit antibodies against the displayed epitope and against the VLP carrier

To ascertain the proportion of the antibody response directed towards the displayed epitope as opposed to the VLP carrier, pooled sera from each group of veVLP immunised mice was also used to probe nativeVLPs (Fig. 1, Panel O). Results broadly demonstrated three distinct profiles; (1) veVLP generated sera recognised nativeVLPs at slightly lower or equivalent titres compared to recognition of respective veVLPs, throughout the immunisation period (9 groups), (2) veVLP sera initially recognised nativeVLP before rapid declining of nativeVLP-specific titres to baseline (3 groups), or (3) veVLP generated sera displayed negligible recognition of nativeVLP (1 group). These results demonstrate that the majority of veVLPs used in these immunisations are capable of eliciting polyspecific antibodies towards the carrier molecule and the venom epitope.

Using ELISA data generated from pooled sera probed against veVLPs and nativeVLP (Fig. 1), we compared the proportion of apparent epitope-specific response of individual group sera by subtracting the 1/500 OD_405_ response against nativeVLP from that of the response against veVLP (Supp. Fig. S8). Apparent epitope-specific antibody responses were identified across all experimental groups by week 14. The apparent epitope specific antibody response to CTX_C veVLPs (Groups A and M, with and without adjuvant, respectively) and sNTX_C (group E) was the greatest of all immunogens examined—with 60–80% of the overall antibody response (Supp. Fig. S8). However, this could ultimately reflect the poor/modest seroconversion of these groups to these antigens. Sera from the remaining veVLP immunised groups typically demonstrated ~ 20% of their anti-veVLP antibody response could be considered specific towards the heterologous displayed epitopes (Supp. Fig. S8). This proportion of epitope specific response is similar to values observed in other studies investigating anti-carrier antibody response^50,51^.

### Antibodies raised against VLPs presenting snake venom epitopes bind the venoms of a geographically and taxonomically diverse panel of elapid snakes

**Figure 2:**
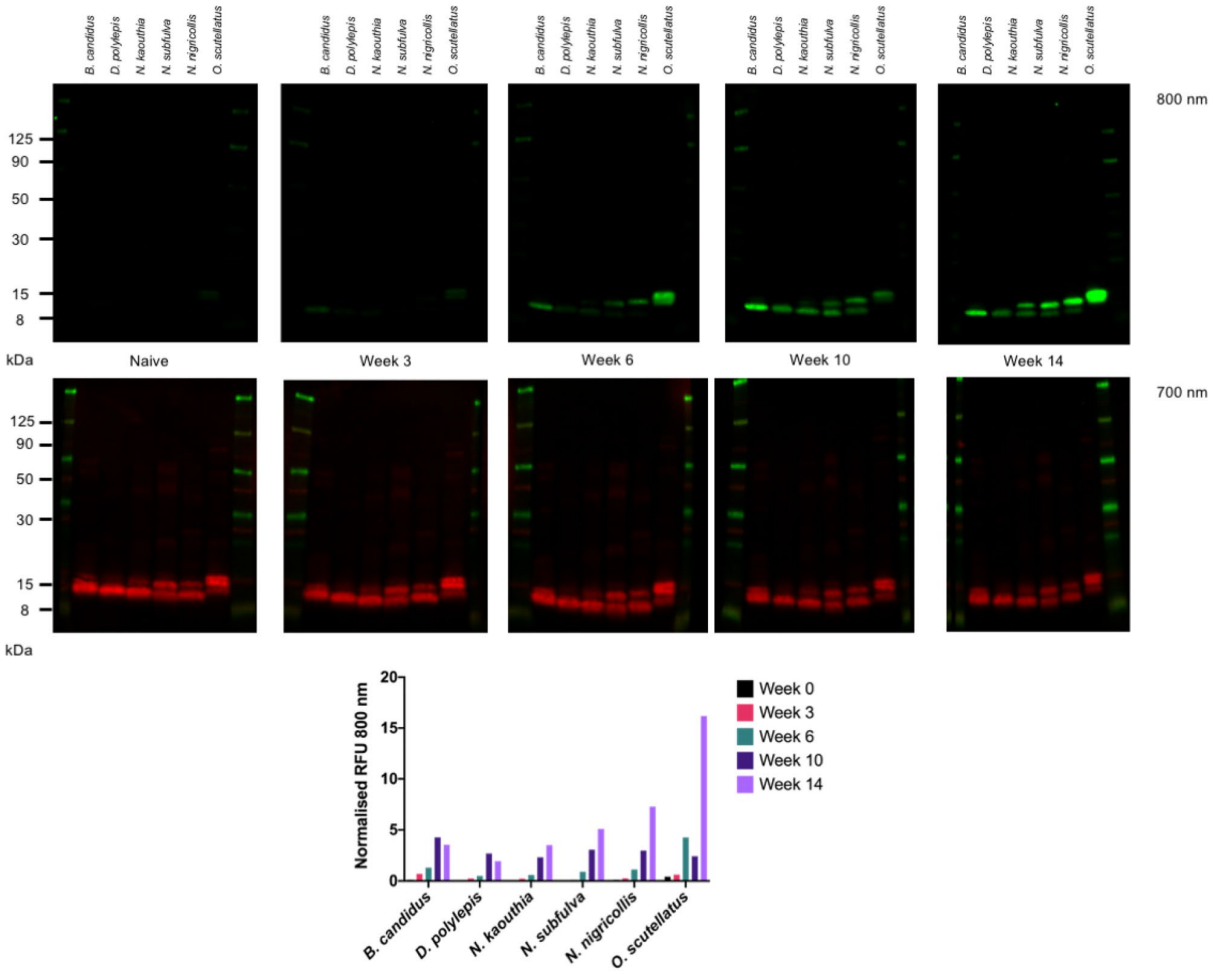
Immuno-blot of elapid snake venoms probed with naïve or veVLP sera. Top: Naïve sera was compared to pooled veVLP sera from all immunisation groups, collected at Weeks 3, 6, 10 and 14 during the experiment. Immuno-blots were performed and imaged for all time points in parallel. Venoms used from left to right were *B. candidus*, *D. polylepis*, *N. kaouthia*, *N. subfulva*, *N. nigricollis* and *O. scutellatus.* Middle panel: Protein loading controls for all blots. Bottom panel: Normalised relative fluorescence units (RFU) in the 800 nm channel for each venom. Multiple gels were used to obtain the five blots above, as indicated by the white space between immuno-blots.

The ability of pools (equal ratio of sera from each immunisation group) of timepoint-specific veVLP generated sera to bind to toxins in *B. candidus*, *D. polylepis*, *N. kaouthia*, *N. subfulva*, *N. nigricollis* and *O. scutellatus* venoms was assessed by fluorescent immunoblotting (Fig. 2). Very low fluorescence signals were initially detectable in sera obtained three weeks post immunisation for five out of six venoms when compared to naïve sera. Fluorescence signals continued to increase at weeks 6 and 10 for all venoms (with the exception of recognition of *O. scutellatus* at week 10). At week 6, recognition of bands corresponding to the expected molecular weights of PLA_2_ toxins were visible in *N. subfulva*, *N. nigricollis* and *O. scutellatus* venoms, but not *N. kaouthia*, for which corresponding bands did not become visibly detectable until week 14 (Fig. 2). No recognition of bands corresponding to PLA_2_ were detected in *B. candidus* or *D. polylepis* venoms at any time point, in line with expectations due to the absence of these specific epitopes in *B. candidus* venom PLA_2_ and the absence of PLA_2_ in *D. polylepis* venom^52^.

Recognition of bands corresponding to 3FTXs were weakly detectable in *D. polylepis* and *N. kaouthia* venoms from week 6, and from week 10 for *B. candidus*, *N. subfulva*, *N. nigricollis* and *O. scutellatus*. At week 14, the intensity of overall recognition of 3FTX from the three *Naja* spp. and *O. scutellatus* venoms had further increased, whilst a slight decrease in overall fluorescence intensity was observed for *B. candidus* and *D. polylepis* (Fig. 2). To test the specificity of the veVLP sera towards the toxins against which they were designed, sera from the best responding animal per immunisation group (as described below) were used in immuno-blot to probe a panel of purified 3FTXs, consisting of muscarinic toxin 3 from *D. angusticeps* (an ATX representative)*,* cytotoxic 3FTX from *N. nigricollis* (a CTX representative) and short chain 3FTX from *N. haje* (a sNTX representative), and a basic group I PLA_2_ from *N. nigricollis*. Across all immuno-blots against purified toxins (Supp. File S7), all sera showed some degree of binding towards the purified PLA_2_, therefore data are shown as fold-difference over naïve signal for normalisation purposes. As shown in Supp. File S7, only one of the three sera raised against veVLP-CTX_C (Group A animal 5) recognised the purified cytotoxic 3FTX; however, these sera did not demonstrate strong recognition of other purified toxins, thereby suggesting toxin specificity. Sera raised against veVLP-ATX_C and veVLP-ATX_F (Groups C and D) and veVLPs displaying 3FTX-strings (Core-string and Finger string, Groups K and L, respectively) showed strong and specific recognition of the purified muscarinic toxin (ATX representative), whilst sera raised against PLA_2_ (Groups H, I and J) displayed limited non-specific recognition of other toxins. Sera raised against sNTX veVLPs (Groups E, F and G) did not demonstrate recognition towards any of the toxins tested.

Dotblots using crude venoms (Supp. Fig. S9) showed specific recognition of venom by the veVLP sera, with no recognition by naïve sera, thus demonstrating that the generated antibodies were able to recognise venom components in their native conformation, as well as in reduced and denatured conditions (i.e. in immuno-blot). Furthermore, the dotblots demonstrated similar specific-venom recognition as observed in immuno-blots; Group D (ATX_F) sera recognised five of the six venoms tested in both immuno-blot and dotblot, with no recognition of *O. scutellatus* apparent. Furthermore, group D [ATX_F] sera from animals D1 and D5, which possessed the broadest and strongest binding to venom components in reduced states (Table 2, Supp. File S6) demonstrated greater recognition of the five venoms in their native state as determined by dotblot (Supp. Fig. S9).

### Comparison of pooled sera to SAIMR Polyvalent antivenom

**Figure 3:**
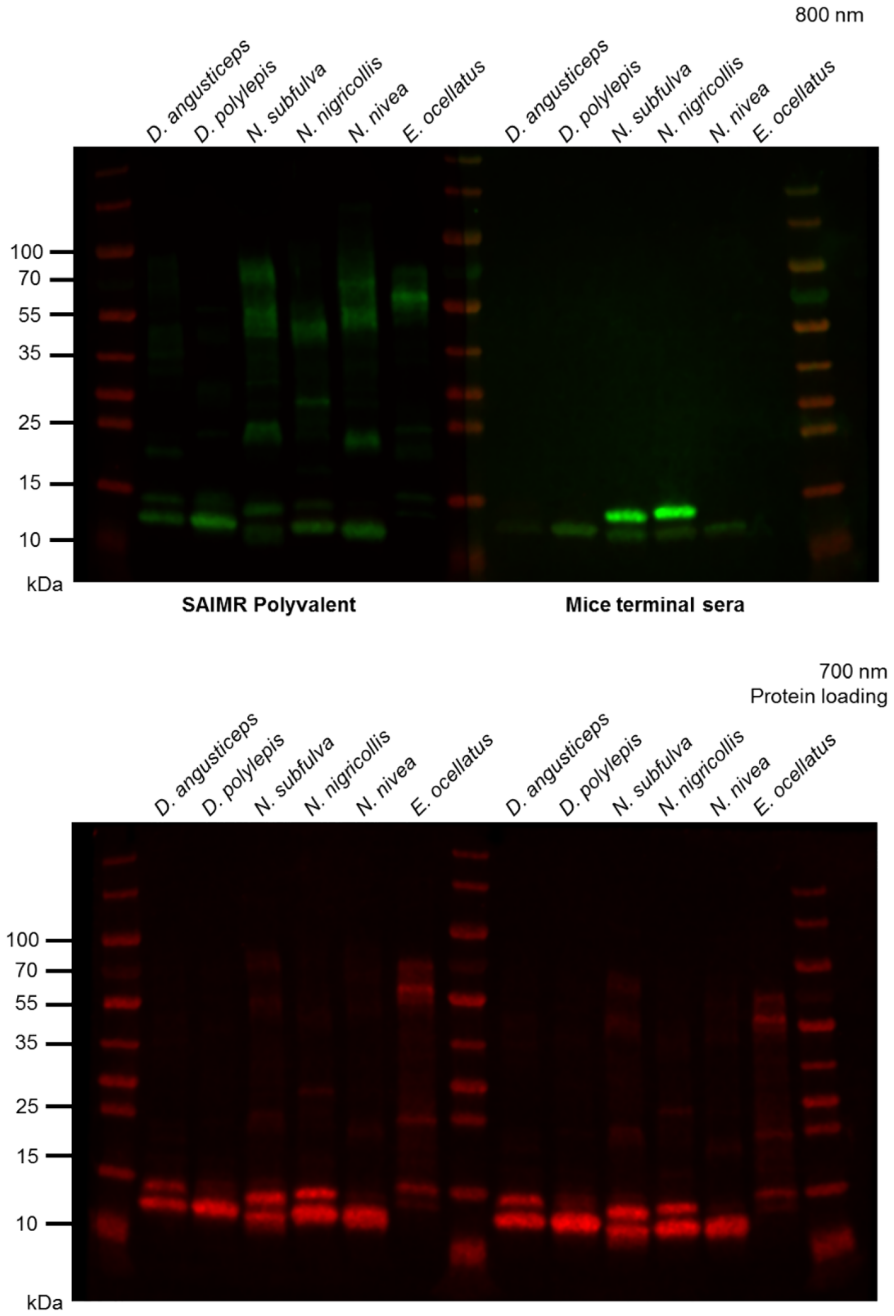
Immuno-blots for qualitative comparison of venom recognition by veVLP mice sera and SAIMR Polyvalent antivenom. Top: Immuno-blots of venoms probed with equal concentrations of SAIMR Polyvalent antivenom (left) or pooled veVLP (right), imaged at 800 nm for 2 min. Immuno-blots were performed and imaged concurrently. Venoms used from left to right were *D. angusticeps*, *D. polylepis*, *N. subfulva*, *N. nigricollis*, *N. nivea* and *E. ocellatus.* Bottom panel: Protein loading controls imaged in 700 nm channel for 2 min. Venoms were loaded onto one gel as shown in the bottom panel, which was cut in half for separate incubations with the different test sera or antivenom. The two halves were then imaged side-by-side for the final image.

The venom recognition profile of pooled veVLP raised sera (from all immunisation groups) was qualitatively compared against SAIMR Polyvalent antivenom by immuno-blot using a panel of sub-Saharan African elapid snake venoms (Fig. 3). The venoms selected represent venoms used (alongside others) in the manufacture of SAIMR Polyvalent antivenom (*D. angusticeps*, *D. polylepis*, *N. subfulva* and *N. nivea*) or are known to be preclinically neutralised by SAIMR Polyvalent (*N. nigricollis*^53^). Additionally, *Echis ocellatus* venom, which is not used in the immunisation mixture for SAIMR Polyvalent and does not contain group I PLA_2_ or 3FTX toxin families^54^ was included as a control. Whilst equivalent protein concentrations were used, the different formats of antibodies tested (murine whole IgG versus equine F(ab)′_2_) and the subsequent differences in recognition by the anti-murine and anti-equine secondary antibodies preclude a quantitative comparison, hence we have only compared the toxin recognition in qualitative terms.

Results demonstrate the generated veVLP mice sera and SAIMR Polyvalent both recognised components of all five elapid venoms. SAIMR Polyvalent additionally recognised *E. ocellatus* venom (likely the result of other related viper venoms being used in the immunisation process), while the veVLP sera did not, as expected. The veVLP sera only recognised lower molecular weight components of the elapid venoms, highlighting the utility of the 3FTX and PLA_2_-specific epitopes designed and implemented in this project, whilst SAIMR Polyvalent recognised both low and higher molecular weight components, the latter most likely corresponding to PIII-SVMPs commonly seen in elapid venoms in low abundances^29^.

### Neutralising ability of veVLP IgG

**Figure 4:**
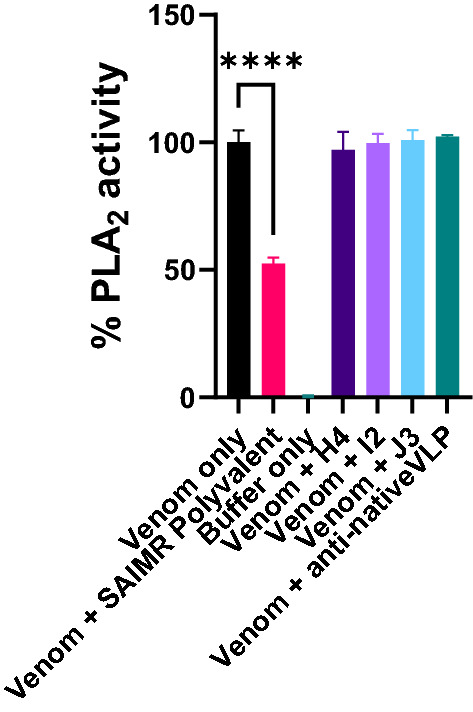
Assays to measure neutralisation of venom PLA_2_ activity by anti-veVLP IgG. The PLA_2_ activity of *Naja nigricollis* venom and inhibitory effects of pre-incubation with SAIMR Polyvalent antivenom or IgG raised against veVLP PLA_2_ epitopes on PLA_2_ activity was measured using the EnzCheck PLA_2_ Assay Kit. Data shows the mean of duplicate repeats and error bars represent standard deviation. The relative fluorescent units (RFU) were translated into % activity, where the mean RFU for venom only represents 100% activity, and Buffer only (no venom) represents 0% activity. The effect of antivenom or test IgG was determined by calculating the remaining activity as a percentage of the venom only controls. One-way ANOVA tests were performed to determine statistically significant results. **** indicates *p* < 0.0001.

We next purified IgG from the terminal sera samples and tested the ability of one animal per immunisation group to neutralise venom activity. The IgG raised against the three PLA_2_ veVLP groups each failed to demonstrate significant neutralisation of *N. nigricollis* PLA_2_ activity whereas the equivalent amount of SAIMR Polyvalent antivenom inhibited 52.4% inhibition of venom activity, as shown in Fig. 4.

### Immune response variation and antibody specificity in individual immunised mice

Immuno-blotting experiments using pooled sera are useful in providing a chronological overview of development of venom component recognition; however, they do not allow resolution of: (1) effectiveness of individual veVLPs to generate antibodies capable of recognising specific venom proteins, or (2) whether immunisation with individual veVLP leads to consistent seroconversion and antibody generation within groups. Furthermore, analysing the serological response of individual animals enabled the identification of the highest-responding mice, facilitating the downstream selection of splenocyte samples from individual mice to progress towards monoclonal antibody isolation in future experiments. Thus, we analysed terminal (week 14) sera by ELISA and immuno-blot, as previously, for all individual immunised animals.

Our results (Table 2, Supp. Files S4, S6) demonstrated that no individual sera in groups immunised with CTX_C with adjuvant, CTX_F, sNTX_C or PLA2_2 (immunisation groups A, B, E, and I respectively) was capable of binding to specific venom proteins found in our panel of elapid venoms (Table 2, Supp. Files S6, S4). This is in contrast to the ELISA results, whereby PLA2_2 veVLP sera demonstrated strong recognition of PLA2_2 veVLPs (Fig. 1) and the observation that group A CTX_C and group E sNTX_C have proportionately high levels of epitope-specific antibodies as determined through ELISA recognition of veVLP (Supp. Fig. S8). The inability of CTX_F (group B) sera to recognise venom components was unsurprising due to near-baseline levels of veVLP recognition in pooled ELISA results (Fig. 1). However, the results from these groups need to be interpreted in the context of several influencing factors. Firstly, ELISA results are demonstrative of veVLP recognition, not crude venom. Secondly, the low final N numbers (n = 1 [group B] or 2 [groups A, E]) due to animal attrition during immunisation (Table 2) in addition to variability within groups that did generate venom-specific antibodies (below), suggest it is possible that the lack of toxin recognition may be the result of individual variation in immune responses.

Sera from at least a single individual immunised with either ATX_C, ATX_F, sNTX_F1, sNTX_F2, PLA2_1, PLA2_3, Core-string, Finger-string and CTX_C (without adjuvant) veVLPs (groups C, D, F, G, H, J, K, L and M, respectively) contained antibodies capable of recognising specific venom proteins present in at least one venom (Table 2, Supp. File S6). The results demonstrate expected specific recognition of individual toxin families (e.g. 3FTX vs PLA_2_) based on the known approximate molecular weights of these toxin groups^48,55^. A notable observation was the high degree of variation in the antibody response towards both the immunogen and venom observed between individual animals in the same immunogen group. For example, within group C, recognition of the immunogen ATX_C veVLP at week 14, as measured by ELISA, was similar for all individuals (mean 1/500 OD_405_ = 1.92, range 1.82–1.98) although recognition of nativeVLP by group C sera was more variable (mean 1/500 OD_405_ = 1.69, range 1.29–1.92) (Table 2, Fig. 1. Supp. File S6). Despite these ELISA results suggesting overall similar recognition of ATX_C veVLPs by mice immunised with ATX_C veVLPs, only sera from animal C5, which is inferred to possess highest proportion of ATX_C antibodies, was able to recognise venom proteins by immuno-blot (Table 2, Supp. File S6), recognising expected 3FTX representative bands in *D. polylepis*, *N. subfulva*, and *N. kaouthia* venoms. Unexpectedly, C5 sera also demonstrated reactivity with an additional, slightly larger, protein in *N. subfulva* and *N. kaouthia* venoms (Supp. File S6). This may potentially be cross-reactivity with a type II long-chain α-neurotoxin, as the ATX_C epitope contains notable similarity (≥ 7 AA alignment length, 80% identity, 80% coverage, mismatches ≤ 1) with members of this toxin subclass (Table S3).

In veVLP immunisation groups where multiple individuals produced antibodies that could recognise venom proteins (groups D, H, K and M, immunised with ATX_F, PLA2_1, Core-string and CTX_F, respectively), the number of venoms recognised was either consistent across individuals (same number of venoms, similar intensity of recognition, e.g. Group D) or variable (recognising different numbers of venoms with differing intensity of recognition, e.g. Group H) (Table 2, Supp. File S6). Two of the three remaining animals in Group D (ATX_F), D1 and D5, possessed antibodies that could recognise 3FTX bands in *B. candidus*, *D*, *polylepis*, *N. subfulva*, *N. kaouthia* and *N. nigricollis* venom with similar intensities (Table 2, Supp. File S6). Conversely, all three remaining animals in Group H, immunised with PLA2_1 veVLP, possessed antibodies capable of recognising bands corresponding to PLA_2_, in different numbers of species (H1 & H5 = 4 venoms, H3 = 2 venoms), though *N. nigricollis* and *O. scutellatus* PLA_2_ was recognised by all three (Table 2, Supp. File S6). CTX_C without adjuvant (Group M) was unusual in that it appeared to show recognition of bands assumed to correspond to PLA_2_ (e.g. M2, Supp. File S6), suggesting potential non-specific binding.

## Discussion

This study demonstrates proof of principle that VLPs decorated with rationally selected conserved linear venom-epitopes can be used to stimulate the production of murine antibodies that are able to recognise a geographically and taxonomically diverse range of elapid venoms (Table 2, Fig. 2, File. S6). Additionally, the promising results and resources generated from this study enable the further progression of this research, as splenocytes isolated from the best-responding individual mice identified in this study are being investigated as a resource for therapeutic anti-toxin monoclonal antibody discovery.

Of the 10 individual epitopes designed and used in this study, six were shown to elicit antibodies capable of binding specific venom components (Table 2, Supp. File S6). veVLPs decorated with 3FTX epitopes corresponding to finger regions were more likely (38%, n = 13) to elicit venom binding sera than core epitope veVLPs (13%, n = 8) at the end of the immunisation period. Additionally, sera against PLA_2_ epitopes PLA2_1 and PLA2_2 (immunisation groups H and I, respectively) demonstrated similar abilities in recognising respective immunogens at the end of the immunisation period (Fig. 1), but provided considerable differences in their ability to recognise venom components, despite differing by only a single amino acid (KGTPVDLDD and KGTAVDDLD, respectively) (Table 1, Supp. File S6). Sera from all remaining animals immunised with PLA2_1 (n = 3) bound proteins in multiple venoms, whilst recognition of venom components was not demonstrated by any of the remaining PLA2_2 immunised animals (n = 4). Such stark difference in venom component recognition suggests that proline may have a key role in antibody recognition of this epitope. Given highly similar toxin epitope sequences can elicit drastically different antibody responses, our findings reiterate that antibodies induced by toxin epitopes need to be robustly assessed for venom recognition against a diverse panel of target venoms in conventional immuno-assays, and not simply based on their apparent frequency in toxin sequences.

Antivenoms have long been reported to have poor dose efficacy in neutralising small molecular weight venom toxins, including 3FTX and group I PLA_2_, which is frequently attributed to these toxins being poorly immunogenic^21,22^. Notably, in this study, a pool of experimental veVLP sera demonstrated recognition of small molecular weight compounds (Fig. 3). Due to the quantities of antibodies recoverable for each animal being very low, we were limited to performing preliminary neutralisation assays against venom PLA_2_ functional activity only. Disappointingly, the results showed no detectable neutralisation of venom PLA activity. Therefore, whilst we have not been able to demonstrate any neutralising ability of the antibodies raised in this study, we are unable to conclude with certainty at this point that is due to the inability of veVLPs to elicit such antibody responses. Further optimisation and study are required.

Despite the success in demonstrating the ability of veVLPs to elicit anti-toxin antibodies, this study was subject to several limitations. Notably, a large proportion of animals during immunisation were euthanised early due to presumed adverse reactions to adjuvant (20 out of 65). Notable local inflammation and irritation (redness and local swelling) were typically observed at all dosing locations in animals that received VLPs and adjuvant, which usually resolved 1–2 weeks post immunisation. Animals that received veVLP without adjuvant (Group M) generally did not develop any local reaction at any dose sites, which leads us to hypothesise the adjuvant, Alhydrogel (alum), was contributing to the observed adverse effects. This is surprising as alum based adjuvants are routinely considered as safe and are widely used in human vaccines^56^, and suggests that the combination of the self-adjuvating nature of VLPs^41^ and the adjuvant might adversely exacerbate local inflammatory responses. Unfortunately, acute local inflammation resulting in a non-resolving lump at the inoculation site was observed in 20 animals dosed at the rump on week 2 (2nd immunisation), which necessitated euthanasia of affected animals as per our institutional and national licence conditions. We suspect that the tighter skin around the rump, as compared to the scruff and flanks of the mice, in combination with irritation and local swelling may have exacerbated local conditions. Due to the substantial adverse reactions observed in this study, we advise against immunising mice in the rump area in similar immunisation experiments conducted in the future. Furthermore, the groups that ultimately were unable to recognise venom components were also the groups most affected by the losses in numbers at week 2 (Table 2). For example, groups A, B and E representing CTX_C_,_ CTX_F and sNTX_C, were the most severely affected, losing 60–80% of their representative animals. Based on the results obtained from groups less affected by animal loss, we currently cannot say if the inability of these epitopes to elicit an immune response is due to poor candidate epitopes or individual variation in response to immunisation. We speculate that the large amount of variation in venom reactivity observed between individuals within a group is due in part to immunological heterogeneity in the experimental animals, as the mouse strain selected, CD1, is outbred and thus reflective of antivenom manufacturing animals. Similar highly variable results in responses to immunisation have been observed in antivenom manufacturing animals^33^ and camels^57^. Finally, repeated attempts to enrich murine IgG using caprylic acid purification failed repeatedly. Control enrichments using caprylic acid and sheep sera performed as expected. We suggest avoidance of caprylic acid enrichment of murine IgG in future studies.

Our results demonstrate that venom epitopes fused to VLPs can induce anti-toxin antibody responses. However, difficulties in production of veVLPS in this specific VLP format (HBcAg), encountered by ourselves and others expressing heterologous antigens^49,58^, may prove challenging for application if this approach were to be applied to producing a rationally designed antivenom at commercial scale. However, research to circumvent production obstacles has been actively undertaken over the past decade. Developments include alternative methods of genetic-fusion for decorating VLPs with heterologous antigens, such as SpyCatcher-SpyTag ‘plug and play’ systems^59^, and the development of computationally designed hyper-stable and soluble synthetic VLPs^49,60^. Use of such particles to generate rationally designed antivenoms, or as a tool to rapidly discover monoclonal antibodies to specific venom targets, may be a more cost-effective approach that would also increase translational viability.

The use of linear snake venom epitopes for rationally targeted anti-toxin antibody generation now has substantial background^26^. Multiple different formats for delivery of linear epitopes have been used, including DNA^27,34^, peptide^34,35,61,62^, and now VLP. Such studies have demonstrated the ability to generate venom-specific antibodies, and several have further demonstrated the ability of epitope generated antibodies to neutralise local and systemic venom pathologies. Whilst considered to be inferior to conformational epitopes in terms of potency, linear epitopes have the advantage that they are easier to identify and cheaper to produce than conformational antigens—vitally important considerations when proposing improvements to a therapeutic which is already prohibitively expensive to the majority of people who need it most^2^. Furthermore, as snake venoms consist of multiple toxin families and sub-families, it is highly likely that such a strategy will require multiple epitopes to ensure adequate protection against all medically important venom components^1^. Recent publication of high-throughput antivenom-venom peptide arrays^47,63^, alongside transcriptomic and proteomic characterisation of venoms^29,64^, means there is now a wealth of resources available for informative venom epitope prediction.

However, substantial research questions remain when considering whether the approach of employing rationally designed linear epitope antigens to elicit anti-toxin antibodies is a genuinely practical method for producing more efficacious antivenoms. Questions include: will the results, all generated so far in mice and rabbits, be translatable in manufacturing antivenom-manufacturing animals? Will antisera produced in this manner possess potencies which at least match existing conventionally produced antivenoms? To date, demonstration of neutralising efficacies of anti-epitope antibodies have been performed against relatively low challenge doses of venom. Could alternative immunisation strategies, such as combined epitope and crude venom approaches, substantially increase efficacy, especially against lower molecular weight toxins? Additionally, through this work we have been able to preserve splenocytes from individual veVLP immunised animals displaying the most promising antibody responses. We hope to investigate this valuable resource with a view to developing mAbs, which have been raised against specific, rationally designed venom epitopes, into potential next generation antivenom therapies^65^.

## Materials and methods

### Animal ethics

Details and reporting of all animal experiments in this manuscript conform with ARRIVE guidelines to the best of our ability. Mouse immunisations were performed using protocols approved by the Animal Welfare and Ethical Review Boards of the Liverpool School of Tropical Medicine and the University of Liverpool. Experiments were conducted under licence approved by the UK Home Office (Project Licence P58464F90) and in accordance with the Animal (Scientific Procedures) Act 1986.

### Murine immunisations

Mice were immunised with venom-epitope displaying VLPs (veVLPs) to investigate their ability to elicit anti-toxin antibody responses. On the day of immunisation, aliquots of immunogen were either: (1) mixed with 2% aluminium hydroxide gel (Alhydrogel, InvivoGen) at a ratio of 1:1 (v/v) and shaken at 1500 rpm for 10 min at room temperature on a ThermoMixer (Eppendorf), or (2) diluted with equal volume PBS when adjuvant was not used. Female CD1 mice (18–20 g) were purchased from Charles River and allowed to acclimatise for one week before first immunisation. Mice were housed in groups of five with ad libitum access to certified reference materials irradiated food (Special Diet Services) and reverse osmosis water (in automatic water system), along with enrichment, and kept at approximately 22 °C at 40–50% humidity, with 12/12 h light cycles. Mice were housed in Techniplast GM500 cages with Lignocel bedding (JRS, Germany) and zigzag fibres nesting material (Sizzlenest, RAJA), and cages were changed fortnightly. Mice were kept in specific-pathogen-free facilities. All experiments were performed by mixed gender experimenters. Humane endpoints were weight loss (> 10% loss of body weight within one week, or > 20% within one month [despite remedial actions such as wet food]), or observation of the following animal behaviour or appearance signs—reduced activity, physiological impairments, pallor, or ulceration following immunisation.

Mice were briefly anaesthetised with 5% isofluorane to enable shaving at the injection site for subsequent monitoring of adverse reactions. Mice were subcutaneously immunised with 1 µg immunogen in a total volume of 40 µL at each immunisation, according to the following schedule; week 0: Injection at one site on the scruff (with adjuvant), week 2: Injection at one site on the rear, week 4: Injection over two sites (20 µL/site) on the right flank (without adjuvant), week 8: Injection over two sites (20 µL/site) on the left flank, week 12: Injection over two sites (20 µL/site) at the scruff. An additional group received GR7_c (Group M) immunisations which were always performed without adjuvant. A total of 12 veVLP immunogens were used for immunisation, with the specific epitope immunogens assigned to each group of five mice listed in Table 1.

Following the second immunisation at week 2, 20 animals developed large non-resolving lumps at the rear dose site and were euthanised on humane grounds to prevent pain, harm and distress. Subsequent immunisations were given over two dose sites in a refinement of the immunisation, from which all animals developed mild, small, self-resolving lumps at the injection sites. Animals were monitored twice per week throughout the course of immunisation for adverse reactions and general health, and no animals were culled due to weight loss or behavioural endpoints being met.

### Sera isolation

Approximately 50 µL venous blood samples were collected at week 3, 6 and 10 by tail bleed. Whole blood was allowed to clot for a minimum of 2 h at room temperature, and sera was obtained by centrifugation at 2000 × g for 10 min at 10 °C. Sera was immediately stored at − 20 °C. Remaining animals were euthanised by rising concentrations of carbon dioxide at week 14 (end of experiment). Following confirmation of death, cardiac punctures were performed to collect whole blood and sera was processed as above. ‘Naïve’ unimmunised mouse sera controls (strain matched) were sourced commercially from Charles River UK and Sigma. Additionally, splenocytes were collected and preserved for future work.

### Venoms, antivenoms and toxins

Venoms were obtained from specimens of *Bungarus candidus* (a historical venom stock collected from snakes of Thai origin) and from *Dendroaspis angusticeps* (Tanzania), *D. polylepis* (Tanzania), *Echis ocellatus* (Nigeria), *N. kaouthia* (captive bred), *N. subfulva* (Uganda), *N. nigricollis* (Nigeria) and *N. nivea* (South Africa), maintained in the herpetarium at the Liverpool School of Tropical Medicine. Crude venoms were immediately frozen, lyophilised and stored at 4 °C until reconstitution. *Oxyuranus scutellatus* venom was obtained from Venom Supplies Pty, Australia. Venoms were resuspended in PBS to 1 mg/mL and stored at − 20 °C. SAIMR Polyvalent Snake Antivenom (South African Vaccine Producers, Gauteng, South Africa; Batch BB01446, expiry date July 2015), which is an equine F(ab)′_2_ antivenom generated against venom immunogens from *Bitis arietans*, *B. gabonica*, *Hemachatus haemachatus*, *D. angusticeps*, *D. jamesoni*, *D. polylepis*, *N. nivea*, *N. melanoleuca*, *N. annulifera* and *N. mossambica*, was used as a control comparator to the generated murine samples. Representative toxins for short chain 3FTX, PLA2 and cytotoxic 3FTX were purified in-house as described in Supplemental Materials and Methods. A representative aminergic-type toxin (muscarinic toxin 3, from *D. angusticeps*) was bought from Alomone Labs (Israel).

### Immunoassays

To assess the toxin recognition and specificity of antibodies from the immunised mice we performed immunoassays comprising of immunoblotting (immuno-blots and dotblots) and end-point ELISAs.

For immuno-blot experiments, 2 µg of purified veVLP, venom or purified toxin was prepared in PBS with equal volume of 2 × denaturing buffer (100 mM Tris-Cl pH 6.8, 20% v/v glycerol, 4% SDS, 0.2% bromophenol blue, 100 mM dithiothreitol), incubated at 100 °C for 5 min and separated on a MiniPROTEAN TGX 4–20% gel for venoms and toxins, or a MiniPROTEAN Any kDa gel for veVLP. Proteins were transferred to nitrocellulose membrane using the TransBlot Turbo system mixed molecular weight programme. Protein loading was visualised using Revert 700 Total Protein Stain (LI-COR Biosciences) according to manufacturer’s instructions and imaged in the 700 nm channel for 2 min on an Odyssey Fc imaging system (LI-COR Biosciences). Membranes were blocked for 2 h at room temperature on an orbital shaker in 5% rabbit serum in tris-buffered saline (TBS) with 0.1% Tween-20 (TBS-T). Membranes were incubated with primary antibody overnight at 4 °C on an orbital shaker at the following dilutions: anti-His monoclonal (Invitrogen MA1-135) at 1 in 2500 in blocking solution, mouse anti-veVLP sera 1 in 500 in blocking solution or, for comparative blots, mouse anti-veVLP sera and equine SAIMR Polyvalent antivenom at 106 µg/mL in blocking solution. The following day, membranes were washed three times for 5 min in TBS-T. Membranes were then incubated in secondary antibody (IRDye 800 CW goat anti-mouse IgG [LI-COR Biosciences] or rabbit anti-horse IgG [H&L] DyLight 800 [Rockland Immunochemicals]) at 1 in 15,000 in Intercept TBS (LI-COR Biosciences) with 0.1% Tween-20 for 2 h at room temperature on an orbital shaker. Membranes were washed a further three times in TBS-T and once in TBS, prior to imaging for 2 min in the 700 and 800 nm channels on an Odyssey Fc Imaging System. All images were obtained using the Image Studio software (Version 5.2, LI-COR Biosciences).

To determine ELISA end-point titres of veVLP immunised mice, antigens, either veVLPs or control VLP displaying no heterologous epitopes (nativeVLP), were coated at 100 ng per well on Nunc MaxiSorp plates (ThermoScientific) in 50 mM carbonate-bicarbonate coating buffer (pH 9.6) and allowed to bind overnight at 4 °C. Plates were washed six times with TBS-1% Tween20, and then blocked with 5% rabbit serum in TBS-1% Tween20 for 8 h at room temperature. Pooled mouse sera was diluted 1 in 100 in blocking solution, added to the plate and five-fold serial diluted to 1 in 500 and 1 in 2500, and incubated overnight at 4 °C. The following day, plates were washed as above and secondary antibody (anti-mouse IgG-HRP, Abcam) at 1 in 2000 in TBS was added for 2 h at room temperature. Plates were washed as above and developed with 3% ABTS in citrate buffer pH 4.0 with 0.1% hydrogen peroxide. Developing solution was added to each well (100 µL per well) and developed for 10 min at room temperature. Reactions were stopped with 100 µL 1% SDS and immediately read at an optical density of 405 nm (OD_405_) on a plate reader (Labtech LT-4500).

All measurements were performed in triplicate, except where indicated (due to limited amounts of sera). Control wells of no protein (with mouse sera and secondary antibody), and no mouse sera (immunogen and secondary antibody) were included. Raw data is available in Supp. File S4.

### Toxin neutralisation assays

IgG was purified from terminal sera using Protein G magnetic beads (PureProteome) as per manufacturers instructions, eluted twice in 0.2 M glycine pH 2.5 and the pH neutralised with 10% v/v 1 M Tris pH 8.5. The IgG were then tested for neutralisation of PLA_2_ activity using the EnzCheck Phospholipase A2 assay kit (ThermoFisher) as per manufacturers instructions (384-well plate format). Assays were performed using 37.5 ng venom (determined as within the linear range for this venom in this assay) and IgG (6.25 µg) per well, after a pre-incubation at 37 °C for 30 min. Samples were cooled to room temperature then transferred to test plate in duplicate. Substrate was added and the plate was incubated in the dark for 30 min at room temperature, then fluorescence read at excitation 485–12 nm emission 515 nm on a FLUOstar Omega (BMG Labtech), with gain set to 80% of the venom only control. Controls included venom only, Tris-glycine buffer only, IgG raised against native VLP and SAIMR Polyvalent antivenom. PLA_2_ activity was determined by subtracting buffer only (background) from all values, then each test condition was expressed as a percentage of the venom only control (100%). Data were analysed by one-way ANOVA using Prism 9.0 (GraphPad).

## Supplementary Information

Supplementary Information 1.

Supplementary Information 2.

Supplementary Information 3.

Supplementary Information 4.

Supplementary Information 5.

Supplementary Information 6.

Supplementary Information 7.

Supplementary Information 8.

Supplementary Information 9.

## Data Availability

All data generated or analysed during this study are either included in this published article (and its Supplementary Information files) or, in the case of raw data files for fluorescent immunoblots, available from the corresponding author upon request.
