# Supplementary material for: Virus-like particles displaying conserved toxin epitopes stimulate polyspecific, murine antibody responses capable of snake venom recognition
Source: Sci Rep. 2022 Jul 5;12:11328. doi: 10.1038/s41598-022-13376-x (PMC9256628; doi:10.1038/s41598-022-13376-x)
Supplement: Supplementary file 1 — Supplementary Information 1. [file 41598_2022_13376_MOESM1_ESM.pdf]

FILE S1 – 3ftx sequences used in this study.

>gr1_N.nubiae_T1865_R_0.0136_L_400_3FTX
LTCVKEKSIFGDTMEICSDGQNLCFKRWHMVVPGRYKKTRGCAATCPIAENRDVIECCSTDKCND
>gr1_N.mossambica.VG_T0824_R_0.1336_L_594_3FTX
LTCVKEKSIFSDTMEICSDGQNLCFKRWHMVVPGRYKKTRGCAATCPIAENRDVIECCSTDKCNN
>gr1_N.ni>griollis.Togo_T582_R_0.1186_L_246_3FTX
LKCVKEKSIFGDTMEICSDGQNLCFKRWHMVVPGRYKKTRGCAATCPIAENRDVIECCSTDKCNN
>gr1_N.ni>gricollis.Tanzania_T0592_R_0.0609_L_467_3FTX
LTCVKEKSIFGDTMEICSDGQNLCFKRWHMVVPGRYEKTRGCAATCPIAQNRDVIECCSTDKCNN
>gr1_N.melanoleuca.VG_T4030_R_0.086_L_448_3FTX
LTCVKEKSIFSDTMETCSDGQNLCFKRWHMVVPGRYKKTRGCAATCPIAENHDVIECCSTDKCND
>gr1_N.kaouthia_T2188_R_0.0172_L_448_3FTX
LTCVKEKSIFGVTTEDCPVGQNLCFKRWHMTVPGRYQKTRGCAATCPIAENRDVIECCSTDKCND
>gr1_N.siamensis_T1083_R_0.031_L_446_3FTX
LTCVKEKSIFGDTTEDCPVGQNLCFKRWHMTVPGRYQKTRGCAATCPIAENRDVIECCSTDKCND
>gr1_N.naja_T1289_R_0.0934_L_413_3FTX
LTCVKEKSIFGVTTEDCPDGQNLCFKRWHMIVPGRYKKTRGCAATCPIAENRDVIECCSTDKCND
>gr2_W.aegyptia_T2204_T2558_R_5.9896_L_450_3FTX
LTCVKERSILGVTTEDCPDGQKLCFKRWHMMAPGRYDITRGCAASCPKAENHDFIKCCSTDKCNL
>gr2_N.pallida_T0906_R_0.1133_L_449_3FTX
LTCVKYYTIFGVTPVDCPDGQNLCFKRWHMMAPGRYDITRGCAATCPKAENHDSIECCSTDKCNL
>gr2_N.nubiae_T1547_R_0.0757_L_448_3FTX
LTCVKYYTIFGVTPVDCPDGQNLCFKRWHMMAPGRYDITRGCAATCPKAENHDFIKCCSTDKCNL
>gr2_N.melanoleuca.VG_T4067_R_0.0265_L_446_3FTX
LTCVKERSILGLTTVDCPDGQNVCFKRWHMMAPGGHDITRGCVATCPKAENHDFIKCCSTDKCNL
>gr2_N.nivea_T0896_R_0.0101_L_445_3FTX
LTCVKYYTIFGVTPVDCPDGQNLCFKRWHMMAPGRYDITRGCAATCPKAQNHDSIECCSTDKCNL
>gr2_N.ni>gricollis.Tanzania_T0657_R_0.0732_L_446_3FTX
LTCVKYYTIFGVTPVDCPDGQNLCFKRWHMMAPGRYDITRGCAATCPKAENHDSIKCCSTDKCNL
>gr3_W.aegyptia_T2393_R_0.7061_L_429_3FTX
LTCVKYYTIFGVTPVDCPDGQNLCFKRWHMMVPGRYDIIRGCAATCPKPENHDPIECCSTDKCNL
>gr4_Aspidelaps.scutatus.intermedius_T0869_R_1.644_L_550
LTCVKERSIYGDTFETCSAGQNLCFRRWHLVYPRKYDITRGCAATCPTAENNDTIDCCSADKCND
>gr4_N.kaouthia_T0184_R_0.0634_L_1398_3FTX
LICVKEKFLFSETTETCPDGQNVCFNQAHLIYPGKYKRTRGCAATCPKLQNRDVIFCCSTDKCNL
>gr4_N.haje_T1223_R_0.2448_L_519_3FTX
LICVKERFLFSETTETCPDGQNLCFNQGHLIYPGKYERTRGCAATCPKLQNRDTIYCCSTDKCNR
>gr4_Aspidelaps.scutatus.intermedius_T1197_R_0.1245_L_450
LICVKERFLFSETTETCPDGQNICFNQGHLIYPGKYERTRGCAATCPILKTRDVIYCCSTDKCNR
>gr4_N.philippensis_T0104_R_0.1459_L_1200_3FTX
LICVKEKFLFSETTETCPDGQNVCFNKAHLIYPGKYKRTRGCAVTCPKLQNCDVIFCCSTDKCNL
>gr4_N.nubiae_T0879_R_0.4457_L_596_3FTX
LICVKERFLFSETTETCPEGQNLCFNQGHLIYPGKYERTRGCAATCPKLQNRDTIYCCSTDKCNR
>gr4_Hemachatus_hemachatus_T1337_R_0.1413_L_467_3FTX
LICVKERFLFSETTVTCTDGQNICFNQGHLIYPGKYERTRGCAATCPKLQNRDVITCCSTDKCNR
>gr5_Dendroaspis.viridis_T0762_0.6051_603
LTCVKTKSIGGVTTEDCPDGQNLCFKRWHYVTPRNYDIIRGCAAACPKADNYDPIRCCGTDKCNE
>gr5_Dendroaspis.jamesoni.kaimosae_T1433_0.615_465
LTCVKTKSIGGVTTEDCPDGQNLCFKRWHYVTPKNYDIIRGCAAACPKADNHDPIRCCGTDKCSE
>gr5_1FF4:A|PDBID|CHAIN|SEQUENCE
LTCVTTKSIGGVTTEDCPAGQNVCFKRWHYVTPKNYDIIKGCAATCPKVDNNDPIRCCGTDKCND
>gr6_3NEQ

LTCVKSNSIWFPTSEDCPDGQNLCFKRWQYISPRMYDFTRGCAATCPKPTNVRETIRCCGTDKCNK
>gr6_2VLW
LTCVKSNSIWFPTSEDCPDGQNLCFKRWQYISPRMYDFTRGCAATCPKAEYRDVINCCGTDKCNK
>gr6_3FEV
LTCVTSKSIFGITTENCPDGQNLCFKRWQYISPRMYDFTRGCAATCPKAEYRDVINCCGTDKCNK
>gr6_Dendroaspis.viridis_T0777_0.1708_598
LTCVTKDTIFGITTQNCPAGQNLCFIRWHYINHRYTEITRGCVATCPKPTNVRETIHCCNTDKCNE
>gr6_4DO84DO8
LTCVTSKSIFGITTENCPDGQNLCFKKWYYIVPRYSDITWGCAATCPKPTNVRETIRCCETDKCNE
>gr6_Dendroaspis.polylepis_T1997_0.212_489
LTCVTKDTIFGITTQNCPAGQNLCFIRQHYVNHRYTEITRGCAATCPKPTNVRETIHCCNTNKCNL
>gr7_Dendroaspis.viridis_T2662_0.09_31
LTCVTSKSIFGITTEDCPDGQNLCFKRRHYVVPKIYDITRGCVATCPKPENYDSIHCCKTDKCNE
>gr7_4IYE
LTCVTSKSIFGITTEDCPDGQNLCFKRRHYVVPAIYDSTRGCAATCPIPENYDSIHCCKTDKCNE
>gr7_Dendroaspis.polylepis_T0423_0.1865_997
LTCVTSKSIFGITTEDCPDGQNLCFKRRHYVAPRIYDITRGCVATCPIPENYDSIHCCKTDKCNE
>gr7_Dendroaspis.viridis_T1215_0.2632_485
LTCVTSKSIFGITTENCPDWQNLCFKRRHYVVPKIYDITRGCVATCPKPENYDSIHCCKTDKCNE
>gr7_Dendroaspis.angusticeps_T1632_0.4372_493
LTCVTSKSIFGITTEDCPDGQNLCFKRRHYVVPKIYDITRGCAATCPIPQNYDSIHCCKTDKCNE
>gr7_Dendroaspis.polylepis_T2320_0.1538_450
LTCVTSKSIFGITTENCPAGQNLCFKRWHYVIPRYTEITRGCVATCPKPENYDSIHCCKTDKCNE
>gr7_5MG9
LTCVKSKSIFGVTTEDCPDGQNLCFKRRHYIVPKMYDSTRGCAATCPIAENRDVIHCCGTDKCNE
>gr8_Dendroaspis.viridis_T0454(2)_T3272_3.7376
LTCVTDKSFGGKITEECAAGQKICFKNWKKMGPKLYDVKRGCTATCPKADDDGCVKCCNTDKCNK
>gr8_Dendroaspis.jamesoni.jamesoni_T3431_T3422_18.0771
LTCVTDKSFGGVITEECAAGQKICFKNWKKMGPKLYDVKRGCTATCPKADDNGCVKCCNTDKCNK
>gr8_Dendroaspis.jamesoni.jamesoni_T1949_0.1841_448
LTCVTRKSLLGISTEVCAAGQKICFKNWKKMGPKLYDVKRGCTATCPKADDDGCVKCCNTDKCNK
>gr8_Dendroaspis.angusticeps_T1269_0.4719_557
LTCVTGKSIGGISTEECAAGQKICFKKWTKMGPKLYDVSRGCTGTCPKADEYGCVKCCNTDRCNK
>gr8_Dendroaspis.viridis_T2812_0.0348_299
LTCVTGKSIGGISTEECAAGQKICFKKWTKMGPKLYDVSRGCTGTCPKADEYGCVKCCKTDRCNK
>gr8_Dendroaspis.angusticeps_T1318_0.1197_548
LTCVTGKSIGGISTEECASGQKICFKKWTKMGPKLYDVSRGCTATCPKADEYGCVKCCKTDRCNK
>gr8_Dendroaspis.viridis_T1088_0.4132_51
LTCVTGKSIGGISTEECAAGQKICFKKWTKMGPKLYDVKRGCTATCPKADEYGCVKCCNTDKCNK
>gr9_Ophiophagus_hannah_3200
LTCLTHESLFFETTETCSDGQNLCYAKWFAVFPGARRPDGGCAATCPDKVPLEIVNCCTTDKCNL
>gr10_N.melanoleuca.VG_T3139_R_0.6369_L_504_3FTX
RKCITKYSVGSETSQTCLAGQNLCFKKWKKGKKVLRGCTAACPKPKKDETIQCCAKDKCNL
>gr10_Aspidelaps.scutatus.intermedius_T0707_R_1.7976_L_632
RKCITKYSLGKETSQTCLAGQTLCFKKWKKGKKILRGCTAACPKPKKHESIQCCAKNKCNV
>gr10_GAEP01001969.1_TSA:_Micrurus_fulvius_Mfulv_3FTx4a.seq_mRNA_sequence
TKCLTKFSPGLQTSQTCPAGQKICFKKWKKGKKVSRGCAVTCPKPKKHETIQCCTENNCNR
>gr10_Bungarus_multicintus_bm032_3FTX
RKCLIKYSQANESSKTCPSGQLLCLKKWEIGNPSGKEVKRGCVATCPKPKKNEIIQCCAKDKCNK
>gr10_N.siamensis_T0963_R_0.0659_L_475_3FTX
RLCLSDYSIFSETIEICPDGHNFCFKKFPKGISRLPRVIRGCAATCPKAEARVYYVDCCARDKCNR
>gr10_N.nivea_T0783_R_0.4022_L_493_3FTX
RLCLSDYSIFSETIEICPDGHNFCFKKFPKGITRLPWVIRGCAATCPKAEAQVYYVDCCARDKCNR

>gr10_N.ni>griollis.Togo_T005_T628_R_0.0187_L_2811_3FTX
RLCLSDYSIFSETIEICPDGHNFCFKKFPKGITPVPWVVRGCAATCPKAEAQVYVDCCARDKCNR
>gr11_Aspidelaps.scutatus.intermedius_T1481_R_0.0156_L_390
RLCLTDYSIFSETIEICPDGHNFCFKKFPKGITRLPWVIRGCAATCPKAEAKVYVDCCARDKCNR
>gr11_N.haje_T1255_R_0.3048_L_512_3FTX
RLCLSDYSIFSETIEICPDGHNFCFKKFPKGITRLPWVIRGCAATCPKAEAQVIVECCTTDKCNR
>gr11_Ophiophagus_hannah_1266
RICLTDYSKVSETIEICPDGQNFCFKKFPKGIPFLPWVNRGCAATCPKPEPKVYVDCCARDKCNQ
>gr12_N.sumatrana_T0584_R_0.3515_L_1003_3FTX
LTCLNCPEMFCGKFQTCRNGEKICFKKLQQRRPFSLRYIRGCAATCPGTKPRDMVECCSTDRCNR
>gr12_N.atra_T2120_R_0.0055_L_295_3FTX
LTCLNCPEMFCGKFQICRNGEKICFKKFTNVDPFSLRYIRGCAATCPETKPRDMVECCSTDRCNR
>gr12_N.kaouthia_T2473_R_2.1679_L_421_3FTX
LTCLNCPEMFCGKFQICRNGEKICFKKLHQRRPLSWRFIRGCADTCPVGKPYEMIECCSTDKCNR
>gr12_N.ni>griollis.Togo_T315_R_0.1316_L_450_3FTX
LTCLICPEKYCNKIHTCRNGENQCFKRFYEGNLLGKRYIRGCAATCPVAKPREIVECCSTDKCNR
>gr12_N.ni>gricollis.Tanzania_T1031_R_0.0117_L_340_3FTX
LTCLICPEKYCNKVHTCRNGENQCFKRFYEGNLLGKRYIRGCAATCPVAKPREIVECCSTDKCNR
>gr12_N.ni>gricollis.Nigeria_T1372_R_0.2279_L_454_3FTX
LTCLICPEKYCNKVHTCRNGENQCFKRFYEGNLLGKRYTRGCAATCPVAKPREIVECCSTDKCNR
>gr12_N.siamensis_T1672_R_0.0933_L_350_3FTX
LTCLICPEKYCNKVHTCRNGENQCFKRFYEGNQLGKRYIRGCAATCPVGKPREIVECCSTDKCNH
>gr12_N.nubiae_T1150_R_0.8525_L_518_3FTX
LTCLICPEKYCNKIHTCRNGENQCFKRFYEGNLLGKRYTRGCAATCPEAKPREIVECCSTDKCNH
>gr12_N.melanoleuca.VG_T4089_R_0.0791_L_445_3FTX
LTCLICPEKYCNKVHTCRNGENICFKRFYEGNLLGKRYPRGCAATCPEAKPREIVECCSTDKCNH
>gr13_N.naja_T2679_R_0.4111_L_200_3FTX
LTCLICPEKYCNKVHTCLNGEKICFKKYDQRKLLGKRYIRGCADTCPVRKPREIVECCSTDKCNH
>gr13_N.ni>griollis.Togo_T313_R_0.0611_L_450_3FTX
LTCLNCPEKYCNKVHTCRKGENICFKKFDQRKLLGKRYTRGCAATCPVAKPREIVECCSKDICNH
>gr13_N.atra_T1370_T2175_R_1.269_L_398_3FTX
LTCLICAEKYCNKVHTCRNGEKICFKKFDQRKLLALRYIRGCAATCPETKPRDMVECCSTDRCNR
>gr13_N.ni>gricollis.Tanzania_T0698_R_0.454_L_433_3FTX
LTCLNCPEKYCNKVHTCRKGENICFKKFDQRKLLGKRYTRGCAATCPVAKPREIVECCSTDRCNH
>gr13_N.nubiae_T1828_R_0.604_L_408_3FTX
LTCLNCPEKYCNKVHTCRRGENICFKKFDQRKLLGKRYTRGCAATCPEAKPREIVECCSKDVCNH
>gr13_N.haje_T1738_T0494_R_0.2772_L_436_3FTX
LTCFNCPEVYCGNFHTCRNGEKICFKRFDQRKLLGKRYTRGCAATCPVAKPREIVECCSTDRCNH
>gr13_N.nivea_T0849_R_3.9037_L_463_3FTX
LRCLNCPEVFCRNFQTCRNGEKICFKRFDQRKLLGKRYTRGCAVTCPVAKPREIVECCSTDGCNR
>gr13_N.melanoleuca.VG_T4313_T6039_R_2.4187_L_434_3FTX
LTCLNCPEVYCRKFHTCRNGENICFKRFDQRKLLGKRYTRGCAATCPEAKPRETVECCSTDKCNH
>gr13_N.sumatrana_T2005_R_1.1289_L_550_3FTX
LTCLNCPEVFCKKFQTCRNGEKICFKKFDERKLFGKRYIRGCAATCPEAKPREIVQCCSTNKCNH
>gr14_1NTN:A|PDBID|CHAIN|SEQUENCE
ITCYKTPIITSETCAPGQNLCYTKTWCDAWCGSRGKVIELGCAATCPTVESYQDIKCCSTDNCNPHPKQKRP
>gr14_4LFT:A|PDBID|CHAIN|SEQUENCE
RTCNKTFSDQSKICPPGENICYTKTWCDAFCSQRGKRVELGCAATCPKVKAGVEIKCCSTDNCNKFQFGKPRX
>gr15_N.ni>griollis.Togo_T116_R_0.697_L_915_3FTX
RQCTQQKPPFYMNCPEGMNVCYTMFVFESPFKFYTKRGCAATCPKSRVRAKIECCEKDRCN
>gr15_N.ni>gricollis.Tanzania_T1076_R_2.3294_L_328_3FTX
RQCTQQKPPFYMNCPEGMNVCYTMFVFESPFKFYTKRGCAATCPKSRVRAKIECCEKDRCNS
>gr15_N.ni>gricollis.Tanzania_T1595_R_1.4595_L_200_3FTX

RQCTQQKPPFYMNCPEGMNNCYTKRGCAATCPKSRVRAKIECCEKDRCNS
>gr15_N.ni>griollis.Togo_T321_R_0.2758_L_448_3FTX
RQCTQKKPPFYMTCPEGMNICYTMFETRFSFYTKRGCAATCPKSPGRGKVQCCDTDRCNS
>gr15_N.ni>gricollis.Nigeria_T1190_R_1.2224_L_497_3FTX
RQCTQKKPPFYMNCPEGMNICYTMFETRFSFYTKRGCAATCPKSPGRGKVQCCDTDRCNS
>gr16_Bungarus_multicintus_bm002_3FTX
IICTRDTYQIPITFTNCEEGHVCYKYSTTETPNRILIHRGCAAKCPKRLRVICCSTDKCNK
>gr16_N.nivea_T0596_R_1.5026_L_587_3FTX
LKCHNTQLPFIYKTCPEGKNLCFKTTLKKLPLKIPIKRGCAATCPKSSALLKVVCCNTDKCN
>gr16_N.annulifera_T0255_R_1.6488_L_726_3FTX
LKCHNTQLPFIYKTCPEGKNLCFKTTLKKLPLKIPIKRGCAATCPKSSALLKVVCCSTDKCN
>gr16_N.philippensis_T0527_R_4.7338_L_500_3FTX
LKCHNTQLPFIYKTCPEGKNLCFKATLKKFPLKIPIKRGCADNCPKNSALLKYVCCSTDKCN
>gr16_N.naja_T1609_T0706_T2091_R_0.5652_L_365_3FTX
LKCHNTQLPIIYKTCPEGKNLCFKMTLKRFPLKFPVKRGCAATCPKSNALLKVVCCSTDKCN
>gr16_N.melanoleuca.VG_T3195_R_0.9751_L_499_3FTX
IKCHNTLLPFIYKTCPEGQNICFKGTLKFPKKTTYKRGCAATCPKSSLLVKYVCCNTNKCN
>gr16_N.nubiae_T1219_R_0.6171_L_503_3FTX
LKCNQLIPPFFKTCPEGKNLCYKITMRMGPRVPIKRGCAPTCPKSNALMKVVCCNTDRCN
>gr16_N.pallida_T1011_T2149_T1010_T0889_R_14.9971_L_422_3FTX
LKCNQLIPPFWKTCPKGKNLCYKMTMRAAPMVPVKRGCIDVCPKSSLLIKYMCCNTDKCN
>gr16_N.ni>gricollis.Tanzania_T0988_R_0.0557_L_350_3FTX
LKCNQLIPPFFKTCPEGKNLCYKMTMRVGPRVPVKRGCASTCPKSNALMKVVCCNTDRCN
>gr16_N.ni>griollis.Togo_T434_T477_R_8.1222_L_350_3FTX
LKCNQLIPPFWKTCPKGKNLCYKMTMRAAPMVPVKRGCIDVCPKSSLLIKYMCCNTDKCN?
>gr16_1TGX:A|PDBID|CHAIN|SEQUENCE
LKCNQLIPPFWKTCPKGKNLCYKMTMRAAPMVPVKRGCIDVCPKSSLLIKYMCCNTDKCN
>gr16_2CCX:A|PDBID|CHAIN|SEQUENCE
LKCNQLIPPFWKTCPKGKNLCYKMTMRAAPMVPVKRGCIDVCPKSSLLIKYMCCNTNKCN
>gr16_N.pallida_T0273_R_7.1252_L_800_3FTX
LKCNKLIPIA?KTCPKGKNLCYKMTMRAAPMVPVKRGCIDVCPKSSLLIKYMCCNTDKCN
>gr16_N.nubiae_T1399_R_5.2232_L_473_3FTX
LKCNRLIPPFWKTCPEGKNLCYKMTMRVAPKVPVKRGCIDVCPKSSLLIKYMCCTNDKCN
>gr16_N.ni>griollis.Togo_T316_T568_R_4.8895_L_450_3FTX
LKCNQLIPPFWKTCPKGKNLCYRITMRGASKVPVKRGCIDVCPKSSLLIKYMCCTNDKCN?
>gr16_N.pallida_T1038_T1323_R_4.1034_L_417_3FTX
LKCNRLIPPFWKTCPEGKNLCYKMTMRLAPKVPVKRGCIDVCPKSSLLIKYMCCTNDKCN
>gr16_N.ni>griollis.Togo_T519_T439_T440_T525_R_21.6264_L_300_3FTX
LKCNQLIPPFWKTCPKGKNLCYRMTMRGASKVPVKRGCIDVCPKSSLLIKYMCCNTDKCN
>gr17_N.ni>gricollis.Nigeria_T1077_T0448_R_1.9408_L_521_3FTX
LKCKRLIPPFWKTCPEGKNLCYKMTMRVAPRVPVKRGCIDVCHKSSLLIKYVCCNTDKCN
>gr17_N.nubiae_T0014_R_0.6882_L_2500_3FTX
LKCNLHIPPFLETCPKGKNLCYKMRVPMTPMVPAKRGCIDVCPKSSLLVKYMCCNTDRCNR
>gr17_N.ni>gricollis.Nigeria_T1631_R_1.0852_L_407_3FTX
LKCNLHIPPFWETCPEGKNLCYKMRVPMAPMVPVKRGCIDVCPKSSLLMKVVCCNTDRCNR
>gr17_N.pallida_T0738_R_1.3375_L_510_3FTX
LKCNLHIPPFLETCPKGKNLCYKMRVPMTPMVPAKRGCIDVCPKSSLLIKYMCCNTDKCN
>gr17_N.ni>griollis.Togo_T144_R_1.0836_L_800_3FTX
LKCNLHIPPFWETCPEGKNLCYKMRVPMAPMVPVKRGCIDVCPKSSLLVRYMCCNTDRCNR
>gr18_N.pallida_T2041_T2204_T1569_R_4.7156_L_250_3FTX
LKCNKLIPIAYKTCPEGKNLCYKMMIASKKMVPVKRGCIDVCPKNSALVKYVCCDTDRCN?
>gr18_N.nubiae_T0146_R_2.5076_L_1341_3FTX
LKCNKLIPIAYKTCPEGKNLCYKMMIASKKMVPVKRGCIDVCTKNSALVKYVCCDTDRCN?

>gr18_1CDT:A|PDBID|CHAIN|SEQUENCE
LKCNKLIPIAYKTCPEGKNLCYKMMLASKKMVPVKRGCINVCPKNSALVKYVCCSTDRCN
>gr18_N.ni>gricollis.Tanzania_T0110_R_2.6244_L_981_3FTX
LKCNKLIPIAYKTCPEGKNLCYKMMMVSKKMVPVKRGCIDVCPKDSALVKYVCCSTDRCN
>gr18_N.ni>griollis.Togo_T401_R_4.7195_L_381_3FTX
LKCKKLIPIAYKTCPEGKNLCYKMMMVSKKMVPVKRGCIDVCPKDSALVKYVCCSTDRCN?
>gr19_N.ni>gricollis.Tanzania_T1476_R_1.5859_L_248_3FTX
LKCNQLIPPFWKTCPEGKNLCYKMMLASKKMVPVKRGCIDVCPKDSALVKYVCCSTDRCN
>gr19_N.ni>griollis.Togo_T405_T545_T557_R_1.6943_L_378_3FTX
LKCKKLIPLFSKTCPDWKNLCYKMMIGSKKMVPVKRGCIDVCPKSSFLVKYECCDTDRCN?
>gr19_N.nubiae_T2328_R_4.3868_L_300_3FTX
LKCKKLIPLFSKTCPEGKNLCYKMMIGSKKMVPVKRGCIDVCPKSSFLVKYECCDTDRCN
>gr19_N.pallida_T1076_R_1.7093_L_409_3FTX
LKCKKLIPLFSKTCPEGKNLCYKMMIGSKKMVSVKRGCIDVCPKSSFLVKYECCNTDKCN
>gr19_N.nubiae_T0724_R_4.068_L_650_3FTX
LKCNQLIPPFWKTCPEGKNLCYKMMIGSKKMVPVKRGCIDVCPKSSFLVKYECCDTDRCN
>gr20_N.ni>gricollis.Nigeria_T0802_R_6.4763_L_609_3FTX
LKCNKLIPIAYKTCPEGKNLCYKMMIASKKMVPVKRGCIDVCPKSSLLIKYMCCNTDKCN
>gr20_N.nivea_T1208_T1206_T1209_T0759_T0854_R_26.8046_L_300_3FTX
LKCHKLVPPFWKTCPEGKNLCYKMYMVATPMLPVKRGCIDVCPKDSALVKYMCCNTDKCN
>gr20_N.nivea_T0758_R_31.6904_L_500_3FTX
LKCNQLIPPFWKTCPKGKNLCYKMFMVSTSTVPVKRGCIDVCPKDSALVKYVCCSTDKCN
>gr20_N.annulifera_T0304_T0460_T0852_R_16.2239_L_664_3FTX
LKCHKLVPPVWKTCPEGKNLCYKMYMVATPMMLPVKRGCIDVCPKDSALVKYMCCNTDKCN
>gr20_N.annulifera_T0616_T1228_T0679_T1138_R_20.1164_L_445_3FTX
LKCHKLVPPFWKTCPEGKNLCYKMFMVSTSTVPVKRGCIDVCPKDSALVKYVCCSTDKCN
>gr21_1CB9:A|PDBID|CHAIN|SEQUENCE
LKCKKLVPLFSKTCPAGKNLCYKMFMVAAPHVPVKRGCIDVCPKSSLLVKYVCCNTDKCN
>gr21_N.philippensis_T1257_T0499_R_0.9916_L_249_3FTX
LKCKKLVPLFSKTCPAGKNLCYKMFMVAAPHVPVKRGCIDVCPKSSLLVKYVCCNTDKCN?
>gr21_N.sumatrana_T2718_R_4.1445_L_478_3FTX
LKCNKLVPLFYKTCPAGKNLCYKMYMVATPKVPVKRGCIDVCPKSSLLVKYVCCNTDRCN
>gr21_N.siamensis_T1084_T1737_T1673_R_15.5235_L_446_3FTX
LKCNKLVPLFYKTCPAGKNLCYKMFMVATPKVPVKRGCIDVCPKSSLLVKYVCCNTDRCN
>gr22_N.atra_T1125_T2283_R_4.769_L_450_3FTX
LKCNKLVPLFYKTCPAGKNLCYKMFMVSNIMVPVKRGCIDVCPKSSLLVKYVCCNTDRCN?
>gr23_N.philippensis_T0602_T0925_R_10.0231_L_474_3FTX
LKCNKLIPLAYKTCPAGKNLCYKMFMVSNLTIPVKRGCIDVCPKNSLLVKYVCCNTDRCN
>gr23_N.naja_T1211_T1304_T0704_R_21.0887_L_427_3FTX
LKCNKLIHLAYKTCPAGKNLCYKMDMVSNKTVPVKRGCIDVCPKNSLLVKYECCNTDRCN
>gr23_1RL5:A|PDBID|CHAIN|SEQUENCE
LKCNKLVPIAYKTCPEGKNLCYKMFMMSDLTIPVKRGCIDVCPKNSLLVKYVCCNTDRCN
>gr23_N.kaouthia_T5505_T3463_R_3.5052_L_200_3FTX
LKCNKLIPIASKTCPAGKNLCYKMFMMSDLTIPVKRGCIDVCPKNSLLVKYVCCNTDRCN
>gr23_N.naja_T2420_T2418_T2687_T1672_R_9.469_L_250_3FTX
LQCNKLVPIASKTCPPGKNLCYKMFMVSDLTIPVKRGCIDVCPKNSLLVKYVCCNTDRCN
>gr23_N.melanoleuca.VG_T8396_T3487_R_10.8994_L_270_3FTX
IECNKLVPIAHKTCPAGKNLCYQMYMVSKSTIPVKRGCIDVCPKSSLLVKYVCCNTDRCN?
>gr24_N.siamensis_T1081_R_24.4653_L_446_3FTX
RKCNKLVPLFYKTCPAGKNLCYKMFMVSDLTVPVKRGCIDVCPKNSALVKYVCCNTDRCN?
>gr25_Hemachatus_hemachatus_T1274_T1588_T1866_T1092_T1440_T1175_R_27.3468_L_490_3FTX
LKCHNKLVPYLSKTCPNGKNLCYKMSMQVTPMIPIKRGCTDTCPKSSLLVKVVCCKTDKCN
>gr26_Dendroaspis.viridis_T3493_T3274_6.1922

MICYSHKTPQDSATITCEEKTCYKKFVTKLPGVILARGCGCPKKEVFRSIHCCRSDKCNE
>gr26_Dendroaspis.jamesoni.jamesoni_T1637_3.4044_494
MICYSHKTPQDSATITCEEKTCYKKFVTKLPGVILARGCGCPKKEIFRSIDCCRSDKCNE
>gr26_Dendroaspis.jamesoni.jamesoni_T3920_T3924_T3915_10.1585
MICYSHKTPQHSATITCEEKTCYKKFMTKVPVFIIARGCGCPKKEVFRSIDCCRSDKCNE
>gr26_Dendroaspis.viridis_T1420_0.5978_448
MICYSHKTPQPSATITCEEKTCYKKSVRKLPAIVAGRGCGCPSKEMLVAIHCCRSDKCNE
>gr26_Dendroaspis.viridis_T1449_0.0285_445
MICYSHNTIMPERLKTCEENSCYREYLRRIAGITNRRGCGCPSAEPLFEIDCCTSDKCNV
>gr26_Dendroaspis.jamesoni.jamesoni_T0135_0.2225_1651
RICYSHKLLQAKATKTCAENSCYKWTLFKIPLFIIARGCGCPLTLPFLQVQCCTSDKCN
>gr26_Dendroaspis.jamesoni.kaimosae_T1803_0.4791_410
RICYSHKLLQAKATKTCAENSCYKRTLFKIPLFIIARGCGCPLTLPFLQVQCCTSDKCN
>gr26_Dendroaspis.viridis_T1500_1.0715_437
RICYSHKLLQAKTTKTCAENSCYKRTLFKIPLFIIARGCGCPLTLPFLQVQCCTSDKCN
>gr27_Dendroaspis.viridis_T1773_0.3513_400
RICYTHKSLQAKTTKTCEENSCYKMFIRTSREYISERGCGCPTAMWPYQTECCKGDRCNK
>gr27_1TFS:A|PDBID|CHAIN|SEQUENCE
RICYSHKASLPRATKTCVENTCYKMFIRTHREYISERGCGCPTAMWPYQTECCKGDRCNK
>gr27_Dendroaspis.viridis_T0121_0.3559_1270
RICYSHKASLPRATKTCVENSCYKMFIRTSPDYISDRGCGCPTAMWPYQTACCKGDRCNK
>gr27_Dendroaspis.jamesoni.jamesoni_T2286_0.2665_410
RICYTHKSLQAKTTKTCEGNSCYKMFIRTSREYISERGCGCPTAMWPYQTECCKGDRCNK
>gr27_Dendroaspis.polylepis_T0010_0.5529_2974
RICYIHKASLPRATKTCVENTCYKMFIRTQREYISERGCGCPTAMWPYQTECCKGDRCNK
>gr28_Dendroaspis.viridis_T1453_0.1637_444
LECYEHDKVVTCKSWEKFCSKRMIVDEPNFLVITRSCATGCLEERNHVCCRSDKCNF
>gr28_Dendroaspis.jamesoni.kaimosae_T1276_0.4093_494
LECYEHDKVVTCKSWEKFCSKRMIVDEPNFLVITRSCATECLEERNHVCCRSDKCNF
>gr28_Dendroaspis.jamesoni.jamesoni_T2404_0.009_397
LECYQHDKVVTCQSWEKFCSKQFIVDHPNFRVITRSCATKCTEERNHVCCKSDKCNY
>gr28_Dendroaspis.viridis_T0298_0.0825_892
LECYHQHTVVTCQPWEKFCTKRCIVAGPRFVVFIRDCATECEQEGNRVCCQTDRCNK
>gr28_2MFA:A|PDBID|CHAIN|SEQUENCE
LKCFQHGKVVTCHRDMKFCYHNTGMPFRNLKLILQGCSSSCSETENNKCCSTDRCNK
>gr28_2MJY:A|PDBID|CHAIN|SEQUENCE
LKCYQHGKVVTCHRDMKFCYHNTGMPFRNLKLILQGCSSSCSETENNKCCSTDRCNK
>gr28_Dendroaspis.viridis_T1572_T0845_0.7219
LKCYQHGKVVTCHRDMKFCYHNIGMPFRNLKLILQGCSSSCSETENNKCCSTDRCNK
>gr28_Hemachatus_hemachatus_T1290_R_3.1744_L_483_3FTX
LECYQKSKVVTCQPEQKFCYSDTTMFFPNHPVYLSGCTFSCTEEGNRRCCTTDKCNR
>gr28_N.melanoleuca.VG_T3680_R_0.0064_L_470_3FTX
LVCNGMDKPVTCEPTQKFCYRKTVMYIRNHPMLTRGCANKCPEKPYSACCTTDKCN
>gr29_N.ni>griollis.Togo_T517_R_0.7318_L_300_3FTX
LRCYSCGRNGCRDIVACSEKQEFCFNRFGKRIQRRTSGCAVKCNPRDSLNVHIQCCTTDLCN
>gr29_N.mossambica.VG_T1809_R_1.0478_L_400_3FTX
LRCYSCGRNGCRDIVTCSEKQEFCFNRFGKRIQRRTSGCAVKCNPRDSLNAHIQCCTTDLCN
>gr29_N.ni>gricollis.Tanzania_T1461_R_0.1858_L_250_3FTX
?SCGRNGCRDIVTCSEKQEFCFNRFGKRIQRRASGCAVKCNPRDSLNAHIQCCTTDLCN
>gr29_N.nubiae_T1549_R_0.6286_L_448_3FTX
LRCYSCGRNGCRDIVTCSEKQEFCFNRFGKRIQSRKSGCAVKCNPRDSLNVQVQCCTTDLCN
>gr29_N.melanoleuca.VG_T4448_R_0.704_L_427_3FTX
LQCYSCGRNGCRDIVTCSEKQEFCFNRFGKRNQLRKSGCAVKCNPRSSSNFRVECCTTDLCN

>gr29_Dendroaspis.viridis_T1196_0.1131_488
LQCYKCRIYPCPLKTTCSPEEKFCFRLLYITRILSRSGCAKTCTEEKSWIFYRTCCTTNFCNR
>gr29_Dendroaspis.polylepis_T0104_0.2056_1789
LECYRCGVSGCHLKITCSAEEKFCCKQHNRISTLWWRGCVKTCTEEDTWKFYRKCCTTNLCNI
>gr29_Dendroaspis.jamesoni.jamesoni_T3918_1.232_250
LQCYRCAIYPCHFKTTCSPEEKFCFKFLDVKYTMARTGCAKTCTEEKSWIFYKKCCTTNLCNS
>gr29_Dendroaspis.angusticeps_T2327_1.1601_404
LECYRCGVSSCHLKITCSAEEKFCCKQHNRISTLWWRGCVKTCTEEDTWTFYRKCCTTDLCNI
>gr29_Dendroaspis.viridis_T2124_0.3175_360
LECYRCGVSGCPLRTTCSAKEKFCAKQHNRISTLWWYGCVETCTEEDTWKFYQKCCTTNLCNI
>gr29_Dendroaspis.angusticeps_T2931_T4404_0.242
LECYTCGVFGCYHKTTCSTEQKFCCKQHNRILGVDWYGCVQTCTEEDNWKVYRKCCTTDLCNV
>gr29_Dendroaspis.polylepis_T0322_0.2892_1117
LECYTCGVFGCYHKTTCSTEQKFCCKQHNRILGVDWYGCVQTCTEEDNWKVYRKCCTTNLCNI
>gr30_Dendroaspis.viridis_T2055_0.0538_369
LECYKCGASGCRLKITCSTEGKFCSKRLNRISKLKWRGCAKTCIEEDNRWFYRKCCTTDLCNT
>gr30_Dendroaspis.jamesoni.kaimosae_T1487_0.5682_456
LECYRTNKYGAHLKTTCSAEETFCFKFRSEITKLKWRGCTDTCFEEENQWFYRKCCPTNLCNA
>gr30_Dendroaspis.jamesoni.jamesoni_T0964_0.1455_662
LECYKCGASGCRLKITCSAEEKFCSKWLDRISKLKWRGCAKTCIEEDNGWFYRKCCTTNLCNT
>gr31_Dendroaspis.polylepis_T2720_0.0252_414
LQCYRCGVSGCHLKTTCSTEETFCSNYASFPVGLNWYGCAKTCTEKNTWSGYNKCCTTNLCNA
>gr31_Dendroaspis.viridis_T2201_0.0485_350
LECYTCDDSGCNLKITCSAEETFCYKWLFKKTNEQWLACAKTCTEEHSWMHYMKCCTTDLCNT
>gr31_Dendroaspis.angusticeps_T1932_0.0778_450
LQCYRCGVSGCHLKTTCSAEETFCSNYASFPVGLNWYGCAKTCTEKNTWSGYNKCCTTNLCNA
>gr31_Dendroaspis.jamesoni.kaimosae_T1903_0.0663_398
LECYTCDDSGCNLKITCSAEETFCYKWLFKKTNEQWLACAKPCTEEHSWMHYMKCCTTDLCNT
>gr31_Dendroaspis.polylepis_T3700_0.0268_348
LECYTCDESGCNLKITCSAEEKFCYKWLFKKTNEQWLECAKTCTEEHSWMHYVKCCTTDLCNT
>gr31_Dendroaspis.jamesoni.jamesoni_T2574_0.0338_383
YTCDDSGCNLKITCSAEETFCYKWLFKKTNEQWLACAKTCTEEHSWMNYMKCCTTVLCNT
>gr31_1IJC
MECYRCGVSGCHLKITCSAEETFCYKWLNKISNERWLGCAKTCTEIDTWNVYNKCCTTNLCNT
>gr31_1F94
MECYRCGVSGCHLKITCSAEETFCYKWLNKISNERWLGCAKTCTEIDTWNVYNKCCTTNLCNT
>gr31_Bungarus_multicintus_bm047_3FTX
MECYRCGVSGCHLKITCSAEETFCYKWLNKISNERWLGCAKTCTEKDTWNVYNKCCTTNLCNT
>gr31_N.melanoleuca.VG_T4455_R_0.0618_L_427_3FTX
MECYKCGVSGCHLKITCSAEETFCYKWHNKILNLRWHGCAKTCTEEDSWKAYIKCCTTNLCNT
>gr31_N.nivea_T0879_R_0.0627_L_449_3FTX
MECYKCGVSGCHLKITCSAEETFCYKWHNKILNLRWHGCAKTCTEEDTWKAYIKCCTTNLCNT
>gr31_Aspidelaps.scutatus.intermedius_T0893_R_0.1778_L_543
MECYRCGVSGCHLTIICSAEETFCYKWHNKISNERWHGCAKTCTEEDTWKAYIKCCTTNLCNT
>gr31_N.nubiae_T1068_R_0.1356_L_537_3FTX
MECYKCGASGCHLKITCSAEEKFCYKWKNKISKLRWHGCAKTCTEEDSWKAYIKCCTTNLCNI
>gr32_GAEP01001975.1_TSA:_Micrurus_fulvius_Mfulv_3FTx8.seq_mRNA_sequence
MECYRCGVSGCHLKITCAADEKFCYKWYNKISSVRTYGCAETCTEENSWTAWVRCCTTNLCNT
>gr33_GAEP01001955.1_TSA:_Micrurus_fulvius_Mfulv_3FTx14.seq_mRNA_sequence
RQCYVGYDVSKFVTCPEGETYCYTTAITARPTHVIIRGCISSCAWYYIKCCATDKCND
>gr34_N.haje_T0731_R_0.231_L_658_3FTX
TKCYNHLSRTPETTEICPYSWHFCYKISWVDGHEGRIERGCTFTCHELRPNGKYVYCCRKDKCNQ
>gr34_N.annulifera_T0412_T0015_R_0.109_L_552_3FTX

TKCYNPLSRTPETTEICPYSWHFCYKISWVDGHEGRIERGCTFTCPELRPNGKYVYCCRKDKCNQ
>gr34_N.pallida_T1133_R_0.1578_L_398_3FTX
TKCYNHLSRTPETTEICPYSWHFCYKMSWVDGHEGRIERGCTFTCPELRPNGKYVYCCRRDKCNQ
>gr34_N.melanoleuca.VG_T0922_T6993_R_0.1491_L_891_3FTX
TKCYNHLSRTPETTEICPDSWYFCYKISWVDGHEGLIERGCTFTCPELRPNGKYVYCCRKDKCNQ
>gr34_Aspidelaps.scutatus.intermedius_T0787_T2093_R_0.2595_L_589
TKCYNHLSRTPETTEICPDSDYFCYKISWIDGGEGRIERGCTFTCPELRPNGKYVYCCKRDKCNQ
>gr34_Hemachatus_hemachatus_T0492_R_0.0918_L_857_3FTX
TKCYNHLSRNPETTEICPDSWYFCYKVSWIDGGEGRIERGCTFTCPELRPNGKYVYCCRRDKCNQ
>gr35_3HH7
TKCYNHQSTTPETTEICPDSGYFCYKSSWIDGREGRIERGCTFTCPELTPNGKYVYCCRRDKCNQ
>gr35_Dendroaspis.polylepis_T2101_0.041_476
TTCYNQLSTTPETTEICPDSQYFCYKKSSIDGDEVRIERGCTFSCPELRPNGKYVYCCRRDKCNQ
>gr35_Dendroaspis.jamesoni.kaimosae_T2168_0.3141_366
TTCYNHLSTTPETTEICPDSQYFCYKKSSIDGDEVRIERGCTFSCPELRPNGKYVYCCRRDKCNQ
>gr35_Dendroaspis.viridis_T1282_0.0361_47
TTCYNHLSTTPETTEICPDSQYFCYKKSGDGNHEVRIERGCTFSCPELRPNGKYVYCCRRDKCNQ
>gr35_Dendroaspis.jamesoni.kaimosae_T1720_0.3509_422
MTCYNHLSTTPETTEICPYTWYFCYKSSSINGDEVRIERGCTFSCPELIPNRNYIYCCRRDKCNQ
>gr35_Dendroaspis.polylepis_T2377_0.0443_446
MTCYNHLSTTPETTEICPYTWYFCYKSSSINGDEVRIERGCTFSCPELIPNRNYVYCCRRDKCNQ
>gr35_Dendroaspis.angusticeps_T0095_0.0254_1754
TTCYNHLSTTPETTEICPYTWYFCYKSSLINGDEVRIERGCTFSCPELIPNRNYVYCCRRDKCNQ
>gr36_N.sumatrana_T1405_R_0.0214_L_650_3FTX
TICYNHLSRTPETIEICPDSRYFCYKISLADGNDVRIKRGCTFTCPELRPTGKYVYCCRRDKCNQ
>gr36_N.kaouthia_T0551_R_0.0427_L_847_3FTX
TICYNHLTRTSETTEICPDSWYFCYKISLADGNDVRIKRGCTFTCPELRPTGIYVYCCRRDKCNQ
>gr36_N.philippensis_T0375_R_0.4121_L_600_3FTX
TICYNHLSRTPETTEICPDS?KFCYKISLADGNDVRIERGCTFTCPELRPTGIYVYCCRRDKCNQ
>gr37_Bungarus_multicintus_bm077_3FTX
KTCFNDDLTNPKTTELCRHSMYFCFKNSWIAGGVERIERGCSLTCPDIKYNGKYIYCCTRDNCNA
>gr37_N.melanoleuca.VG_T2728_R_0.0456_L_542_3FTX
TKCYNHLSGTPETTEICPDSQYFCYKSSWIDGGGMRIKRGCVASCPEFSSHYKSLVCCRTENCNK
>gr37_Aspidelaps.scutatus.intermedius_T0374_T1715_R_0.1329_L_881
FKCFTTSSGKSETCPNGQNICYEVGWIEHQGMRIKKGCDASCPEFSSHFQSLLCCRIENCNQ
>gr38_1DRS:A|PDBID|CHAIN|SEQUENCE
RICYNHLGTKPPTTETCQEDSCYKNIWTFDNIIRRGCGCFTPRGDMPGPYCCESDKCNL
>gr38_Dendroaspis.viridis_T2758_0.1932_302
RICYNHLGTKPPTTETCQEDSCYKNIWTFDNIIRRGCGCFTPRGDMPGPYCCESDKCNL
>gr38_2LA1
RICYNHLGTKPPTTETCQEDSCYKNIWTFDNIIRRGCGCFTPRGDMPGPYCCESDKCNL
>gr38_Dendroaspis.jamesoni.kaimosae_T1905_1.4663_397
RICYNHLGTKPTTTETCQEDSCYKNIWTFYNIIRRGCGCFTPRGDMPGPYCCESDKCNL
>gr38_Dendroaspis.viridis_T0454_2.1034_747
LICYNQLGTKPPTTETCGDDSCYKMIWTYDGVIRRGCGCFTPRGDMPGPYCCESDKCNL
>gr39_1NTX:A|PDBID|CHAIN|SEQUENCE
RICYNHQSTTRATTKSCEENSCYKKYWRDHRGTIIERGCGCPKVKPGVGIHCCQSDKCNY
>gr39_Dendroaspis.polylepis_T1284_4.084_601
RICYNHQSTTRATTKSCEENSCYKKYWRDHRGTIIERGCGCPKVKPGVGIHCCQSDKCNY
>gr39_Dendroaspis.viridis_T1801_0.0989_398
RICYNHQSTTRATTKSCEENSCYKKYWRDHRGTIIERGCGCPKVKPGVGIRCCQSDKCNY
>gr39_Dendroaspis.jamesoni.jamesoni_T1225_1.9355_579
RICYNHQSTTPATTKSCGENSCYKKTLSDHRGTIIERGCGCPNVKQGIHLHCCQSDKCNN

>gr39_Dendroaspis.jamesoni.kaimosae_T0532_T2409_15.659
RICYNHQSTTPATTKSCGENSCYKKTWSDHRGTIIERGCGCPKVKQGIHLHCCQSDKCNK
>gr39_Dendroaspis.viridis_T1436_1.7317_446
RICYNHQSTTPATTKSCGENSCYKKTWSDHRGTIIERGCGCPKVKRGVHLHCCQSDKCNN
>gr40_Dendroaspis.viridis_T0913_1.1303_552
RICYNHQSDTPATTKSCVENSCYKSIWADHRGTIIKRGCGCPRVKSKIKIKCCKSDNCNL
>gr40_Dendroaspis.angusticeps_T1380_T2642_1.2989
MICYTHQSTAPETTTSCGDYFCYKKSWDNHHGHQIIRGCGCPIGKPGIKTQCCKIDKCNY
>gr40_Dendroaspis.polylepis_T0156_0.1393_1493
?ICYTHQSTAPETTTNCGDYFCYKKSWDNHHGHQIIRGCGCPIGKPGIKTQCCKIDKCNY
>gr40_Dendroaspis.jamesoni.jamesoni_T0569_0.2439_866
TICYTHQSTAPETTTSCGDYFCYKKSWDNHHGHQIIRGCGCPIGKPGIKTQCCKIDKCNA
>gr40_Dendroaspis.viridis_T0410_0.1088_786
TICYTHQSTAPETTTSCGDYFCYKKSWDNHHGHQIIRGCGCPTGKPGIKTQCCKINKCNG
>gr41_Dendroaspis.viridis_T1815_0.0119_396
RICHSQMSSQPPTTTFCRVNSCYRRTLRDPHDHRGTIIVRGCGCPRMKPGTKLECCTSDKCNV
>gr41_Dendroaspis.jamesoni.kaimosae_T0935_0.8994_575
RICHSQMSSQPPTTTFCRVNSCYRRTLRDPHDPRGTIIVRGCGCPRMKPGTKLECCTSDKCNV
>gr41_Dendroaspis.polylepis_T2411_0.1747_443
RICHSQMSSQPPTTTFCRVNSCYRRTLRDPHDPSGTIVVRGCGCPRMKPGTKLKCCTSDKCNV
>gr41_Dendroaspis.angusticeps_T2301_0.167_406
RICHNQMSSQPPTTITCRVNSCYRKSWSNNPGTIIVRGCGCPKMKPGAKLNCCTSDKCNV
>gr41_Dendroaspis.viridis_T1757_0.067_403
RICHNQMSSQPPTTITCRVNSCYRKSWSNNPGTIIVRGCGCPKMKPGTKLNCCTSDKCNV
>gr41_Dendroaspis.jamesoni.jamesoni_T3991_0.027_242
RICHNQMSSQPPTTITCRVNSCYKKSWSNNPGTIIVRGCGCPKMKPGTKLNCCTSDKCNV
>gr42_Aspidelaps.scutatus.intermedius_T1194_T1195_T2313_T0508_R_0.3648_L_450
IICHNQMSSQNPTTQDCPRESNCYKMRWSNIHGSITERGCGCPTVKPGISLICCKTDKCNN
>gr42_N.ni>griollis.Togo_T212_R_0.1473_L_593_3FTX
KICYKQQALQIPIPTVCIGEKYCYKMQWSGNRGTIIKRGCGCPSVKKGIKINCCTTDKCNR
>gr42_N.ni>gricollis.Tanzania_T0638_R_0.1703_L_449_3FTX
KICYKQQALQFPIPTVCIGEKYCYKMQWSGNRGTIIKRGCGCPSVKKGIKINCCTTDKCNR
>gr42_N.nivea_T0742_R_0.1614_L_512_3FTX
MICYKQRSLQFPITTVCPGEKNCYKKQWSGHRGTIIERGCGCPSVKKGIEINCCTTDKCNR
>gr42_N.haje_T2831_T1704_T3906_T3905_T0971_R_16.6986_L_337_3FTX
MICHNQQSSQPPTIKTCPGETNCYKKQWRDHRGTIIERGCGCPSVKKGVGIYCCKTDKCNR
>gr42_N.nivea_T0481_T1201_R_5.3063_L_693_3FTX
TICHNQQSSQRPTIKTCPGETNCYKKRWRDHRGTIIERGCGCPSVKKGVGIYCCKTNKCNR
>gr43_1ONJ
LECHNQQSSQTPTTKTCSGETNCYKKWWSDHRGTIIERGCGCPKVKPGVNLNCCTTDRCNN
>gr43_1VB
LECHNQQSSQTPTTKTCSGETNCYKKWWSDHRGTIIERGCGCPKVKPGVNLNCCTTDRCNN
>gr43_N.atra_T0611_R_7.475_L_626_3FTX
LECHNQQSSQAPTTKTCSGETNCYKKWWSDHRGTIIERGCGCPKVKPGVNLNCCTTDRCNN
>gr43_N.siamensis_T2470_T2140_R_0.5824_L_230_3FTX
LECHNQQSSQAPTTKTCSGETNCYKKWWSDHRGTIIERGCGCPKVKPGVKLNCCTTDRCNN
>gr43_N.naja_T2381_T2098_T2382_T2412_T2627_T2517_R_2.4938_L_253_3FTX
LECHNQQSSQPPTTKTCSGETNCYKKWWSDHRGTIIERGCGCPKVKPGVNLNCCRTDRCNN
>gr43_1NOR:A|PDBID|CHAIN|SEQUENCE
LECHNQQSSQPPTTKTCSGETNCYKKWWSDHRGTIIERGCGCPKVKPGVNLNCCRTDRCNN
>gr43_N.philippensis_T0670_T1332_T1255_T1331_R_32.5637_L_446_3FTX
LECHNQQSSQPPTTKTCSVETNCYKKWWSDHRGTIIERGCGCPKVKPCVKLNCCRTDRCNN
>gr44_N.philippensis_T1247_T0775_T1071_R_10.6019_L_250_3FTX

LECHNQQSSQAPTTTCCPGGETNCYKKWWSGHCGTVIERGCGCPKVKPGVKLNCCRTDRCNN
>gr44_1NEA:A|PDBID|CHAIN|SEQUENCE(2)
LECHNQQSSQPPTTKTCPGETNCYKKVWRDHRGTIIERGCGCPTVKPGIKLNCCTTDKCNN
>gr44_3NDS
LECHNQQSSQPPTTKTCSPGETNCYKKVWRDHRGTIIERGCGCPTVKPGIKLNCCTTDKCNN
>gr44_N.pallida_T0954_R_4.0814_L_438_3FTX
MICHNQQSSQPPTTKTCPGETNCYKKVWRDHRGTIIERGCGCPTVKPGIKLNCCTTDKCNN
>gr44_Hemachatus_hemachatus_T1315_T1868_R_2.0847_L_475_3FTX
LECHNQQSSQPPTTKSCPGETNCYNKRWRDHRGTIIERGCGCPTVKPGIKLKCCTTDRCNN
>gr45_Aspidelaps.scutatus.intermedius_T0042_T1559_R_0.2565_L_2150
MQCHNQQSSQPPTTTCCSGGESNCYKKQWSDTRGIIIERGCGCPRVKKGIKLHCCTTEKCNN
>gr45_N.kaouthia_T4638_R_1.1816_L_280_3FTX
LECHNQQSSQTPTTTGCSGGETNCYKKRWRDHRGYRTERGCGCPSVRNGIEINCCTTDRCNN
>gr45_Hemachatus_hemachatus_T0992_R_1.4158_L_576_3FTX
LECHNQQSSETPTTQTCPGETNCYKKQWSDHRGSRTERGCGCPTVKPVIKLKCCTTDRCNK
>gr45_W.aegyptia_T1024_T3273_R_7.7193_L_659_3FTX
FVCHNQQSSQPPTTTNCSGGENKCYKKRWSTHRGTITERGCGCPTVKKGIELHCCTTDQCNL
>gr45_N.atra_T0366_R_5.3042_L_811_3FTX
LECHNQQSSQTPTTTGCSGGETNCYKKRWRDHRGYRTERGCGCPSVKNGIEINCCTTDRCNN
>gr45_N.siamensis_T1032_R_1.3924_L_459_3FTX
LECHDQQSSQTPTTTGCSGGETNCYKKRWRDHRGYRTERGCGCPSVKNGIEINCCTTDRCNN
>gr45_N.naja_T0917_R_0.5252_L_490_3FTX
LECHNQQSSQTPTTTDCSGGETNCYKKRWRDHRGYRSERGCGCPTVKKGIEINCCTTDRCNN
>gr45_N.naja_T0481_R_0.2118_L_647_3FTX
LECHNQQSSQPPTTTCCSGETNCYKKRWSDHRGYRTERGCGCPSVGNGIEINCCTTDRCNN
>gr45_Hemachatus_hemachatus_T1235_R_0.6644_L_498_3FTX
LECHNQQSSQPPTTTCCSGDTNCYKKRWRDHRGSITERGCGCPTVKKGIEINCCTTDRCNN
>gr45_N.pallida_T1763_T2211_R_1.1365_L_300_3FTX
LECHNQQSSQPPTTTCCSGAETNCYKKRWRDHRGTIIERGCGCPTVKKGIELNCCTTDRCNN
>gr46_N.ni>griollis.Togo_T172_R_0.1064_L_697_3FTX
LECHNQQSSEPPTTTRCSGGETNCYKKRWRDHRGYRTERGCGCPTVKKGIELNCCTTDRCNN
>gr46_N.ni>gricollis.Tanzania_T0024_R_0.2678_L_1873_3FTX
LDCQNQQSSEPPTTTRCSRWETNCYKKRWRDHRGYRTERGCGCPTVKKGIQLHCCTTDNCNN
>gr46_N.nubiae_T1520_T0243_R_1.7056_L_450_3FTX
LDCHNQMSAQPPTTTRCSRWETNCYKKRWRDHRGYKTERGCGCPTVKKGIQLHCCTTDNCNN
>gr46_N.ni>griollis.Togo_T267_R_2.0189_L_499_3FTX
LNCHNQMSAQPPTTTRCSSGETNCYKKRWRDHRGYKTERGCGCPTVKKGIQLHCCTSDNCNN
>gr46_N.ni>gricollis.Nigeria_T0050_R_0.1266_L_2041_3FTX
LECHNKQSSEPPTTTRCSGGETNCYKKRWRDHRGYRTERGCGCTTVKKGIELNCCTTDRCNK
>gr46_N.ni>gricollis.Nigeria_T0400_R_0.1835_L_844_3FTX
LDCHNQQSSEPPTTTRCSSGETNCYKKRWRDHRGYRTERGCGCPTVKKGIQLHCCTTDNCNN
>gr46_N.mossambica.VG_T0206_R_0.2685_L_1095_3FTX
LNCHNQMSAQPPTTTRCSRWETNCYKKRWRDHRGYKTERGCGCPTVKKGIQLHCCTSDNCNN
>gr47_N.siamensis_T1064_R_0.0074_L_450_3FTX
LECHNQQSSQPPTTKTCPGETNCYKKRWRDHRGSITERGCGCPSVKKGIEINCCTTDKCNN
>gr48_Ophiophagus_hannah_4431
RICLKQEPFQPEPTTTCPEGEDACYNLFWSDHSEIKIEMGCGCPKTEPYTNLYCCKIDSCNK
>gr49_Dendroaspis.jamesoni.kaimosae_T1484_0.3161_456
LICYNDQSVITSTTKTCRTKFCFKATWPIHKGGQVKRGCGCPKVKDAKLTCCTSDKCNY
>gr49_Dendroaspis.viridis_T1138_0.0577_500
LICYNDQSVIHPTTKTCRTKFCFKATWPIHKGGQVKRGCGCPKVKDAKLTCCTSDKCNY
>gr49_GAEP01001952.1_TSA:_Micrurus_fulvius_Mfulv_3FTx11.seq_mRNA_sequence
LMCDNSNVPSIRTPKRCLKNQKLCYKITFFTTGYGWTQKKGCIHRCHESKPDKKVQCCAKKNCR

>gr49_GAEP01001954.1_TSA:_Micrurus_fulvius_Mfulv_3FTx13.seq_mRNA_sequence
ITCYKRHASDSQTTTCLSGICYKKITRGRYQPEMGCGCPESRRGVKVDCCMSDKCNA
