# Supplementary material for: Virus-like particles displaying conserved toxin epitopes stimulate polyspecific, murine antibody responses capable of snake venom recognition
Source: Sci Rep. 2022 Jul 5;12:11328. doi: 10.1038/s41598-022-13376-x (PMC9256628; doi:10.1038/s41598-022-13376-x)
Supplement: Supplementary file 2 — Supplementary Information 2. [file 41598_2022_13376_MOESM2_ESM.pdf]

FILE S2 – 3FTX tree in Newick format:

To view tree – copy/paste below into a suitable Newick tree visualisation tool, e.g.: http://etetoolkit.org/treeview/

(((((((((((((((((N.nubiae_T1865_R_0.0136_L_400_3FTX,N.mossambica.VG_T0824_R_0.1336_L_594_3FTX)0.2480,N.nig
riollis.Togo_T582_R_0.1186_L_246_3FTX)0.2700,N.nigricollis.Tanzania_T0592_R_0.0609_L_467_3FTX)0.4400,N.mel
anoleuca.VG_T4030_R_0.086_L_448_3FTX)0.7920,(N.naja_T1289_R_0.0934_L_413_3FTX,(N.siamensis_T1083_R_0.
031_L_446_3FTX,N.kaouthia_T2188_R_0.0172_L_448_3FTX)0.8940)0.8620)0.8780,((W.aegyptia_T2204_T2558_R_5.
9896_L_450_3FTX,N.melanoleuca.VG_T4067_R_0.0265_L_446_3FTX)0.7180,(W.aegyptia_T2393_R_0.7061_L_429_3
FTX,((N.pallida_T0906_R_0.1133_L_449_3FTX,N.nivea_T0896_R_0.0101_L_445_3FTX)0.5560,(N.nubiae_T1547_R_0.
0757_L_448_3FTX,N.nigricollis.Tanzania_T0657_R_0.0732_L_446_3FTX)0.5520)0.7860)0.9240)0.8760)0.3320,Aspide
laps.scutatus.intermedius_T0869_R_1.644_L_550)0.3820,((N.philippensis_T0104_R_0.1459_L_1200_3FTX,N.kaouthi
a_T0184_R_0.0634_L_1398_3FTX)0.9340,((N.nubiae_T0879_R_0.4457_L_596_3FTX,N.haje_T1223_R_0.2448_L_519
_3FTX)0.8920,(Hemachatus_hemachatus_T1337_R_0.1413_L_467_3FTX,Aspidelaps.scutatus.intermedius_T1197_R_
0.1245_L_450)0.4020)0.8480)1.0000)0.1420,(((Dendroaspis.viridis_T0762_0.6051_603,Dendroaspis.jamesoni.kaimo
sae_T1433_0.615_465)0.9780,'1FF4:A|PDBID|CHAIN|SEQUENCE')0.9560,((((3NEQ,2VLW)0.6620,3FEV)0.6120,(4DO8
4DO8,(Dendroaspis.viridis_T0777_0.1708_598,Dendroaspis.polylepis_T1997_0.212_489)0.9980)0.4100)0.1420,(5M
G9,(Dendroaspis.polylepis_T2320_0.1538_450,((Dendroaspis.viridis_T2662_0.09_31,Dendroaspis.viridis_T1215_0.26
32_485)0.6620,(Dendroaspis.polylepis_T0423_0.1865_997,(4IYE,Dendroaspis.angusticeps_T1632_0.4372_493)0.502
0)0.2880)0.8580)0.6660)0.1960)0.1020)0.1860)0.7540,((('Dendroaspis.viridis T0454(2) T3272
3.7376',Dendroaspis.jamesoni.jamesoni_T3431_T3422_18.0771)0.9540,Dendroaspis.jamesoni.jamesoni_T1949_0.18
41_448)0.8500,(Dendroaspis.viridis_T1088_0.4132_51,(Dendroaspis.angusticeps_T1318_0.1197_548,(Dendroaspis.v
iridis_T2812_0.0348_299,Dendroaspis.angusticeps_T1269_0.4719_557)0.7240)0.6800)0.9160)1.0000)0.3880,Ophio
phagus_hannah_3200)0.1980,((((N.melanoleuca.VG_T3139_R_0.6369_L_504_3FTX,Aspidelaps.scutatus.intermedius
_T0707_R_1.7976_L_632)0.9800,'GAEP01001969.1 TSA: Micrurus fulvius Mfulv 3FTx-4a.seq mRNA
sequence')0.5460,Bungarus_multicintus_bm032_3FTX)0.9460,(Ophiophagus_hannah_1266,(N.haje_T1255_R_0.304
8_L_512_3FTX,(Aspidelaps.scutatus.intermedius_T1481_R_0.0156_L_390,((N.siamensis_T0963_R_0.0659_L_475_3F
TX,N.kaouthia_T2210_R_0.0286_L_446_3FTX)0.2260,(N.nigriollis.Togo_T005_T628_R_0.0187_L_2811_3FTX,(N.nivea
_T0783_R_0.4022_L_493_3FTX,N.nigricollis.Nigeria_T0126_R_0.1155_L_1451_3FTX)0.3900)0.2320)0.1400)0.7080)0.
8820)1.0000)0.2040)0.1920,(((N.sumatrana_T0584_R_0.3515_L_1003_3FTX,N.atra_T2120_R_0.0055_L_295_3FTX)0.
8940,N.kaouthia_T2473_R_2.1679_L_421_3FTX)0.6700,(((((N.nigriollis.Togo_T315_R_0.1316_L_450_3FTX,N.nigricoll
is.Tanzania_T1031_R_0.0117_L_340_3FTX)0.4520,N.nigricollis.Nigeria_T1372_R_0.2279_L_454_3FTX)0.5700,N.siam
ensis_T1672_R_0.0933_L_350_3FTX)0.3180,(N.nubiae_T1150_R_0.8525_L_518_3FTX,N.melanoleuca.VG_T4089_R_
0.0791_L_445_3FTX)0.4560)0.9540,((N.naja_T2679_R_0.4111_L_200_3FTX,N.atra_T1370_T2175_R_1.269_L_398_3
FTX)0.2540,(((N.nigriollis.Togo_T313_R_0.0611_L_450_3FTX,N.nigricollis.Tanzania_T0698_R_0.454_L_433_3FTX)0.5
660,N.nubiae_T1828_R_0.604_L_408_3FTX)0.9680,(N.sumatrana_T2005_R_1.1289_L_550_3FTX,(N.melanoleuca.VG
_T4313_T6039_R_2.4187_L_434_3FTX,(N.nivea_T0849_R_3.9037_L_463_3FTX,N.haje_T1738_T0494_R_0.2772_L_4
36_3FTX)0.4840)0.3860)0.3340)0.1920)0.1340)0.5040)1.0000)0.1720,('1NTN:A|PDBID|CHAIN|SEQUENCE','4LFT:A|P
DBID|CHAIN|SEQUENCE')0.6780)0.1180,(((((N.nigriollis.Togo_T116_R_0.697_L_915_3FTX,N.nigricollis.Tanzania_T10
76_R_2.3294_L_328_3FTX)1.0000,N.nigricollis.Tanzania_T1595_R_1.4595_L_200_3FTX)0.8400,(N.nigriollis.Togo_T3
21_R_0.2758_L_448_3FTX,N.nigricollis.Nigeria_T1190_R_1.2224_L_497_3FTX)0.9740)0.9980,Bungarus_multicintus_
bm002_3FTX)0.5200,(((((N.nivea_T0596_R_1.5026_L_587_3FTX,N.annulifera_T0255_R_1.6488_L_726_3FTX)0.9520,
N.philippensis_T0527_R_4.7338_L_500_3FTX)0.6900,N.naja_T1609_T0706_T2091_R_0.5652_L_365_3FTX)0.8500,N.
melanoleuca.VG_T3195_R_0.9751_L_499_3FTX)0.9620,((N.nubiae_T1219_R_0.6171_L_503_3FTX,N.nigricollis.Tanza
nia_T0988_R_0.0557_L_350_3FTX)0.9980,(Hemachatus_hemachatus_T1274_T1588_T1866_T1092_T1440_T1175_R
_27.3468_L_490_3FTX,(((((((N.pallida_T1011_T2149_T1010_T0889_R_14.9971_L_422_3FTX,N.nigriollis.Togo_T434
_T477_R_8.1222_L_350_3FTX)0.2900,'1TGX:A|PDBID|CHAIN|SEQUENCE')0.6780,'2CCX:A|PDBID|CHAIN|SEQUENCE
')0.9540,N.pallida_T0273_R_7.1252_L_800_3FTX)0.4280,((N.pallida_T1038_T1323_R_4.1034_L_417_3FTX,N.nubiae
_T1399_R_5.2232_L_473_3FTX)0.9660,(N.nigriollis.Togo_T519_T439_T440_T525_R_21.6264_L_300_3FTX,N.nigriolli
s.Togo_T316_T568_R_4.8895_L_450_3FTX)0.7640)0.4160)0.2780,N.nigricollis.Nigeria_T1077_T0448_R_1.9408_L_5
21_3FTX)0.6280,((N.pallida_T0738_R_1.3375_L_510_3FTX,N.nubiae_T0014_R_0.6882_L_2500_3FTX)0.8780,(N.nigri
ollis.Togo_T144_R_1.0836_L_800_3FTX,N.nigricollis.Nigeria_T1631_R_1.0852_L_407_3FTX)0.7300)0.9600)0.3940,(((
((((N.pallida_T2041_T2204_T1569_R_4.7156_L_250_3FTX,N.nubiae_T0146_R_2.5076_L_1341_3FTX)0.9460,'1CDT:A
|PDBID|CHAIN|SEQUENCE')0.6940,(N.nigriollis.Togo_T401_R_4.7195_L_381_3FTX,N.nigricollis.Tanzania_T0110_R_2

.6244_L_981_3FTX)0.9300)0.5520,N.nigricollis.Tanzania_T1476_R_1.5859_L_248_3FTX)0.3940,(N.nubiae_T0724_R_
4.068_L_650_3FTX,(N.pallida_T1076_R_1.7093_L_409_3FTX,(N.nubiae_T2328_R_4.3868_L_300_3FTX,N.nigriollis.To
go_T405_T545_T557_R_1.6943_L_378_3FTX)0.8100)0.6900)0.6400)0.1520,N.nigricollis.Nigeria_T0802_R_6.4763_L_
609_3FTX)0.5100,(((N.nivea_T1208_T1206_T1209_T0759_T0854_R_26.8046_L_300_3FTX,N.annulifera_T0304_T046
0_T0852_R_16.2239_L_664_3FTX)0.9020,(N.nivea_T0758_R_31.6904_L_500_3FTX,N.annulifera_T0616_T1228_T067
9_T1138_R_20.1164_L_445_3FTX)0.7500)0.4360,(((('1CB9:A|PDBID|CHAIN|SEQUENCE',N.philippensis_T1257_T049
9_R_0.9916_L_249_3FTX)1.0000,(N.sumatrana_T2718_R_4.1445_L_478_3FTX,N.siamensis_T1084_T1737_T1673_R_
15.5235_L_446_3FTX)0.9300)0.3880,N.atra_T1125_T2283_R_4.769_L_450_3FTX)0.3140,(N.siamensis_T1081_R_24.
4653_L_446_3FTX,((N.philippensis_T0602_T0925_R_10.0231_L_474_3FTX,N.naja_T1211_T1304_T0704_R_21.0887_
L_427_3FTX)0.4020,(N.melanoleuca.VG_T8396_T3487_R_10.8994_L_270_3FTX,(N.naja_T2420_T2418_T2687_T167
2_R_9.469_L_250_3FTX,('1RL5:A|PDBID|CHAIN|SEQUENCE',N.kaouthia_T5505_T3463_R_3.5052_L_200_3FTX)0.416
0)0.6000)0.2380)0.1820)0.1900)0.4160)0.1300)0.2900)0.5780)0.5040)0.7860)0.9000)0.3480)0.0500,(((((((Dendroasp
is.viridis_T3493_T3274_6.1922,Dendroaspis.jamesoni.jamesoni_T1637_3.4044_494)0.9680,Dendroaspis.jamesoni.ja
mesoni_T3920_T3924_T3915_10.1585)0.9800,Dendroaspis.viridis_T1420_0.5978_448)0.9940,Dendroaspis.viridis_T
1449_0.0285_445)0.4340,(Dendroaspis.viridis_T1500_1.0715_437,(Dendroaspis.jamesoni.kaimosae_T1803_0.4791_
410,Dendroaspis.jamesoni.jamesoni_T0135_0.2225_1651)0.5620)1.0000)0.3760,((Dendroaspis.viridis_T1773_0.351
3_400,Dendroaspis.jamesoni.jamesoni_T2286_0.2665_410)1.0000,(Dendroaspis.viridis_T0121_0.3559_1270,('1TFS:
A|PDBID|CHAIN|SEQUENCE',Dendroaspis.polylepis_T0010_0.5529_2974)0.9520)0.9120)1.0000)0.4400,('GAEP0100
1955.1 TSA: Micrurus fulvius Mfulv 3FTx-14.seq mRNA
sequence',((((Dendroaspis.viridis_T1453_0.1637_444,Dendroaspis.jamesoni.kaimosae_T1276_0.4093_494)1.0000,D
endroaspis.jamesoni.jamesoni_T2404_0.009_397)1.0000,Dendroaspis.viridis_T0298_0.0825_892)0.8700,(((('2MFA:A
|PDBID|CHAIN|SEQUENCE','2MJY:A|PDBID|CHAIN|SEQUENCE')0.5460,Dendroaspis.viridis_T1572_T0845_0.7219)1.
0000,(N.melanoleuca.VG_T3680_R_0.0064_L_470_3FTX,Hemachatus_hemachatus_T1290_R_3.1744_L_483_3FTX)0.
4740)0.1220,(((((N.nigriollis.Togo_T517_R_0.7318_L_300_3FTX,N.mossambica.VG_T1809_R_1.0478_L_400_3FTX)0.
8120,N.nigricollis.Tanzania_T1461_R_0.1858_L_250_3FTX)0.4960,N.nubiae_T1549_R_0.6286_L_448_3FTX)0.7480,N
.melanoleuca.VG_T4448_R_0.704_L_427_3FTX)1.0000,((Dendroaspis.viridis_T1196_0.1131_488,Dendroaspis.james
oni.jamesoni_T3918_1.232_250)0.9680,(((((Dendroaspis.polylepis_T0104_0.2056_1789,Dendroaspis.angusticeps_T2
327_1.1601_404)0.8420,Dendroaspis.viridis_T2124_0.3175_360)0.8720,(Dendroaspis.polylepis_T0322_0.2892_111
7,Dendroaspis.angusticeps_T2931_T4404_0.242)0.9980)0.5340,(Dendroaspis.jamesoni.kaimosae_T1487_0.5682_45
6,(Dendroaspis.viridis_T2055_0.0538_369,Dendroaspis.jamesoni.jamesoni_T0964_0.1455_662)0.9560)0.3580)0.114
0,((Dendroaspis.polylepis_T2720_0.0252_414,Dendroaspis.angusticeps_T1932_0.0778_450)1.0000,((((Dendroaspis.
viridis_T2201_0.0485_350,Dendroaspis.jamesoni.kaimosae_T1903_0.0663_398)0.9320,Dendroaspis.polylepis_T370
0_0.0268_348)0.5160,Dendroaspis.jamesoni.jamesoni_T2574_0.0338_383)1.0000,('GAEP01001975.1 TSA: Micrurus
fulvius Mfulv 3FTx-8.seq mRNA
sequence',(((1F94,1IJC)0.7540,Bungarus_multicintus_bm047_3FTX)1.0000,(N.nubiae_T1068_R_0.1356_L_537_3FTX,
(Aspidelaps.scutatus.intermedius_T0893_R_0.1778_L_543,(N.nivea_T0879_R_0.0627_L_449_3FTX,N.melanoleuca.V
G_T4455_R_0.0618_L_427_3FTX)0.9280)0.5460)0.4120)0.7980)0.3020)0.2160)0.1620)0.1640)0.9480)0.3160)0.3540
)0.3640)0.1060)0.0520)0.0140,((((((((((N.haje_T0731_R_0.231_L_658_3FTX,N.annulifera_T0412_T0015_R_0.109_L_
552_3FTX)0.7420,N.pallida_T1133_R_0.1578_L_398_3FTX)0.6140,N.melanoleuca.VG_T0922_T6993_R_0.1491_L_89
1_3FTX)0.6960,(Hemachatus_hemachatus_T0492_R_0.0918_L_857_3FTX,Aspidelaps.scutatus.intermedius_T0787_T
2093_R_0.2595_L_589)0.6580)0.5540,3HH7)0.6280,(((Dendroaspis.polylepis_T2101_0.041_476,Dendroaspis.jameso
ni.kaimosae_T2168_0.3141_366)0.9360,Dendroaspis.viridis_T1282_0.0361_47)0.8100,(Dendroaspis.angusticeps_T0
095_0.0254_1754,(Dendroaspis.polylepis_T2377_0.0443_446,Dendroaspis.jamesoni.kaimosae_T1720_0.3509_422)
0.6500)0.9820)0.5320)0.3960,(N.philippensis_T0375_R_0.4121_L_600_3FTX,(N.kaouthia_T0551_R_0.0427_L_847_3
FTX,(N.sumatrana_T1405_R_0.0214_L_650_3FTX,N.atra_T0325_R_0.1385_L_850_3FTX)0.6820)0.6440)0.9860)0.998
0,Bungarus_multicintus_bm077_3FTX)0.4160,(N.melanoleuca.VG_T2728_R_0.0456_L_542_3FTX,Aspidelaps.scutatu
s.intermedius_T0374_T1715_R_0.1329_L_881)0.4340)0.2660,(Dendroaspis.viridis_T0454_2.1034_747,(Dendroaspis.
jamesoni.kaimosae_T1905_1.4663_397,(2LA1,('1DRS:A|PDBID|CHAIN|SEQUENCE',Dendroaspis.viridis_T2758_0.193
2_302)0.3540)0.8880)0.9840)0.9980)0.0560,(Ophiophagus_hannah_4431,((((('1NTX:A|PDBID|CHAIN|SEQUENCE',D
endroaspis.polylepis_T1284_4.084_601)0.7500,Dendroaspis.viridis_T1801_0.0989_398)0.9980,(Dendroaspis.viridis_
T1436_1.7317_446,(Dendroaspis.jamesoni.kaimosae_T0532_T2409_15.659,Dendroaspis.jamesoni.jamesoni_T1225_
1.9355_579)0.6300)0.9700)0.9420,Dendroaspis.viridis_T0913_1.1303_552)0.9180,(Dendroaspis.viridis_T0410_0.108
8_786,(Dendroaspis.jamesoni.jamesoni_T0569_0.2439_866,(Dendroaspis.polylepis_T0156_0.1393_1493,Dendroaspi

s.angusticeps_T1380_T2642_1.2989)0.5340)0.4480)1.0000)0.2500,(((((Dendroaspis.viridis_T1815_0.0119_396,Dendroaspis.jamesoni.kaimosae_T0935_0.8994_575)0.7740,Dendroaspis.polylepis_T2411_0.1747_443)1.0000,(Dendroaspis.jamesoni.jamesoni_T3991_0.027_242,(Dendroaspis.viridis_T1757_0.067_403,Dendroaspis.angusticeps_T2301_0.167_406)0.5680)0.9980)0.9800,Aspidelaps.scutatus.intermedius_T1194_T1195_T2313_T0508_R_0.3648_L_450)0.1560,((((N.nigriollis.Togo_T212_R_0.1473_L_593_3FTX,N.nigricollis.Tanzania_T0638_R_0.1703_L_449_3FTX)1.0000,N.nivea_T0742_R_0.1614_L_512_3FTX)0.9520,(N.nivea_T0481_T1201_R_5.3063_L_693_3FTX,N.haje_T2831_T1704_T3906_T3905_T0971_R_16.6986_L_337_3FTX)0.9980)0.1700,((((((((1ONJ,1VB)0.7360,N.atra_T0611_R_7.475_L_626_3FTX)0.4740,N.siamensis_T2470_T2140_R_0.5824_L_230_3FTX)0.5240,(N.philippensis_T0670_T1332_T1255_T1331_R_32.5637_L_446_3FTX,('1NOR:A|PDBID|CHAIN|SEQUENCE',N.naja_T2381_T2098_T2382_T2412_T2627_T2517_R_2.4938_L_253_3FTX)0.8700)0.4180)0.6740,N.philippensis_T1247_T0775_T1071_R_10.6019_L_250_3FTX)0.8080,(Hemachatus_hemachatus_T1315_T1868_R_2.0847_L_475_3FTX,(N.pallida_T0954_R_4.0814_L_438_3FTX,(3NDS,'1NEA:A|PDBID|CHAIN|SEQUENCE(2)')0.8120)0.7380)0.5160)0.1780,Hemachatus_hemachatus_T0992_R_1.4158_L_576_3FTX)0.1120,(N.siamensis_T1064_R_0.0074_L_450_3FTX,((Aspidelaps.scutatus.intermedius_T0042_T1559_R_0.2565_L_2150,W.aegyptia_T1024_T3273_R_7.7193_L_659_3FTX)0.3840,((((((N.kaouthia_T4638_R_1.1816_L_280_3FTX,N.atra_T0366_R_5.3042_L_811_3FTX)0.4760,N.siamensis_T1032_R_1.3924_L_459_3FTX)0.9000,N.naja_T0917_R_0.5252_L_490_3FTX)0.4700,N.naja_T0481_R_0.2118_L_647_3FTX)0.3420,(N.pallida_T1763_T2211_R_1.1365_L_300_3FTX,Hemachatus_hemachatus_T1235_R_0.6644_L_498_3FTX)0.4520)0.1200,((N.nigriollis.Togo_T172_R_0.1064_L_697_3FTX,N.nigricollis.Nigeria_T0050_R_0.1266_L_2041_3FTX)0.7920,((N.nigricollis.Tanzania_T0024_R_0.2678_L_1873_3FTX,N.nigricollis.Nigeria_T0400_R_0.1835_L_844_3FTX)0.7320,(N.nigriollis.Togo_T267_R_2.0189_L_499_3FTX,(N.nubiae_T1520_T0243_R_1.7056_L_450_3FTX,N.mossambica.VG_T0206_R_0.2685_L_1095_3FTX)0.5140)0.7880)0.7780)0.2120)0.2700)0.2120)0.1340)0.2800)0.1240)0.3160)0.2500)0.1360)0.0300,('GAEP01001954.1 TSA: Micrurus fulvius Mfulv 3FTx-13.seq mRNA sequence',('GAEP01001952.1 TSA: Micrurus fulvius Mfulv 3FTx-11.seq mRNA sequence',(Dendroaspis.viridis_T1138_0.0577_500,Dendroaspis.jamesoni.kaimosae_T1484_0.3161_456)1.0000)0.2120)0.0340);
