# Supplementary material for: Virus-like particles displaying conserved toxin epitopes stimulate polyspecific, murine antibody responses capable of snake venom recognition
Source: Sci Rep. 2022 Jul 5;12:11328. doi: 10.1038/s41598-022-13376-x (PMC9256628; doi:10.1038/s41598-022-13376-x)
Supplement: Supplementary file 3 — Supplementary Information 3. [file 41598_2022_13376_MOESM3_ESM.pdf]

FILE S3: PLA2 sequences used in this study

>sp|P07037|PA2A2_ASPSC_Acidic_phospholipase_A2_CMII_OS_Aspidelaps_scutatus_OX_8607_PE_1_SV_1
NLYQFKNMIQCTVPNRSWWHFADYGCFCGYGGSGTPVDELDRCCQTHDNCYSEAEKLSGCKPYIKTYSYDCSQGKLTCSGNDDKCA
AFVCNCDRVAAICFAGAPYIDDNYNVDLNERCQ
>Bungarus_multicintus_bm008_PLA2
LNLYQFKNMIVCAGTRPWIGYVNYGCYCGAGGSGTPVDELDRCCYVHDNCYGEAEKIPGCNPKTKTYSYTCTKPNLTCTDAAGTCARI
VCDCDRTAAICFAAAPYNINNFMISSSTHCQ
>H.haemachatus_1668_1780_2004_1701_1692_3.1101_PLA2
NLYQFKNMIHCTVPSRPWWHFADYGCYCGRGGKGTPIDDLDRCCQVHDNCYEKAGKMGCWPYFTLYKYKCSKGTLTCNGRNGKC
AAAVCNCDLVAANCFAGAPYINANYNIDFKKRCQ
>H.haemachatus_1740_3.01868863137008_PLA2
NLYQFKNMIHCTVPSRPWWHFADYGCYCGRGGKGTPVDDLDRCCQVHDNCYEKAGKMGCWPYFTLYKYKCSQGKLTCSGGNSKC
GAAVCNCDLVAANCFAGARYIDANYNINFKKRCQ
>H.haemachatus_1749_3.01868863137008_PLA2
NLYQFKNMIHCTVPSRPWWHFADYGCYCGRGGKGTPVDDLDRCCQVHDNCYEKAGKMGCWPYLTLYKYKCSQGKLTCSGGNSKC
GAAVCNCDLVAANCFAGARYIDANYNINFKKRCQ
>H.haemachatus_1863_1839_3.5358_PLA2
LNLYQFKNMIHCTVPSRPWWHFADYGCYCGRGGSGKPVDDLDRCCQVHDNCYGKAEKLGCWPYLTLYKYECSQGKLTCSGGNNKC
QAAVCNCDLVAANCFAGAPYIDANYNVNLKERC
>H.haemachatus_1874_2.2335996692157_PLA2
LSTSIFKNMIKCTVPSRSWWHFADYGCYCGRGGSGTPVDDLDRCCQTHDNCYTEAEKISGCRPYFKTYSYDCTKGKLTCKEGNNECAA
FVCKCDRLAAICFAGAHYNDDIYNIDLARHCQ
>H.haemachatus_1937_2.2335996692157_PLA2
NLYQFKNMIHCTVPNRSWWHFANYGCYCGRGGSGTPVDDLDRCCQIHDNCYGEAEKISGCWPYIKTYTYDSCQGTLTSCGAANNC
AASVCDCDRVAANCFARAPYIDKNYNIDFNARCQ
>H.haemachatus_2135_0.203742840146347_PLA2
NLYQFKNMIQCTVPSRSWCDFADYGCYCGKGGSGTPVDDLDRCCQVHDNCYNEAEKISGCWPYFKTYSYECSQGTLTCKGGNNACA
AAVCDCDRLAAICFAGAPYTDANYNIDLKARCQ
>H.haemachatus_2138_0.203742840146347_PLA2
LNLYQFKNMIQCTVPSRSWWDFADYGCYCGRGGSGTPVDDLDRCCQVHDHCYNEAEKISGCWPYSKTYSYECSQGTLTCKGGNNA
CAAAVCDCDRLAAICFAGAPYNNNNYNIDLKARCQ
>H.haemachatus_2149_0.203742840146347_PLA2
NLYQFKNMIQCTVPNRSWWDFADYGCYCGRGGSGTPVDDLDRCCQVHDNCYDEAEKISRCWPYFKTYSYECSQGTLTCKNGNNAC
AAAVCDCDRLAAICFAGAPYNNNNYNIDLKARCQ
>H.haemachatus_2152_0.203742840146347_PLA2
NLYQFKNMIQCTVPSRSWWDFADYGCYCGKGGSGTPVDDLDRCCQVHDNCYNEAEKISGCWPYFKTYSYECSQGTLTCKGGNNAC
AAAVCDCDRLAAICFAGAPYTDANYNIDLKARCQ
>H.haemachatus_3070_2465_3348_4.5595_PLA2
LNLYQFKNMIHCTVPSRPWWHFADYGCYCGRGGKGTPVDDLDRCCQVHDNCYEKAGKMGCWPYFTLYKYKCSQGKLPCSGGNNK
CQAAVCNCDLVAANCFAGAPYIDANYNVNLKERCQ
>H.haemachatus_3233_2128_1909_3151_7.3928_PLA2
LNLYQFKNMIHCTVPSRPWWHFADYGCYCGRGGTGTAVDDLDRCCQVHDNCYGEAEKLGCWPYLTLYKYECSQGKLTCSGGNNKC
QAAVCNCDLVAANCFAGAPYIDANYNVNLKERCQ
>H.haemachatus_3512_2306_2111_0.1206_PLA2
LNLYQFKNCHCTVPSRPWWHFADYGCYCGRGGKGTPIDDLDRCCQVHDNCYEKAGKMGCWPYFTLYKYKCSKGTLTCNGRNGKCA
AAVCNCDLVAANCFAGAPYINANYNIDFKKRCQ
>H.haemachatus_3647_2173_1868_3199_2690_7.4683_PLA2
NLYQFKNMIHCTVPSRPWWHFADYGCYCGRGGKGTAVDDLDRCCQVHDNCYGEAEKLGCWPYLTLYKYECSQGKLTCSGGNNKCE
AAVCNCDLVAANCFAGAPYIDANYNVNLKERCQ
>H.haemachatus_3822_0.0698727473440407_PLA2
NLYQFKNMIKCTVPSRSWLDFANYGCYCGRGGSGTPVDDLDRCCQIHDNCYNEAGKISGCWPYFKTYSYECSQGTLTCKGDNNSCA
ASVCDCDRLAAICFAGAPYNNDNYNINLKARCQ

>N.atra_1792_0.00135074896071917_PLA2
NTYQFKNMIQCTVPKRSWWDFADYGCYCGRGGSGTPIDDLDRCCQVHDNCYNSAREQGGCRPKQKTYSYECKAGTLSCSGSNNSC
AATVCDCDRLAAICFAGAPYNDNNYNIDLKARCQ
>N.atra_1809_0.0178352843700573_PLA2
LNLYQFKNMVQCTVPNRSWWDFADYGCYCGRGGSGTPVDDLDRCCQVHDNCYGEAEKISRCWPYFKTYSYECSQGTLTCKGGNN
ACAAAVCDCDRLAAICFAGAPYNDNNYNIDLKARCQ
>N.atra_1813_1.03394919476848_PLA2
LNLYQFKNMIQCTVPSRSWWDFADYGCYCGRGGSGTPVDDLDRCCQVHDHCYNEAEKISGCWPYSKTYSYECSQGTLTCKGGNNA
CAAAVCDCDRLAAICFAGAPYNNNNYNIDLKARCQ
>N.atra_1819_1797_1837_1853_1856_1808_1880_2.2202_PLA2
LNLYQFKNMIQCTVPSRSWWDFADYGCYCGRGGSGTPVDDLDRCCQVHDNCYNEAEKISGCWPYFKTYSYECSQGTLTCKGGNNA
CAAAVCDCDRLAAICFAGAPYNNNNYNIDLKARCQ
>N.atra_1844_0.0265031908885762_PLA2
LNLYQFKNMIQCTVPSRSWWDFADYGCYCGRGGSGTPVDDLDRCCQVHDNCYNEAEKISGCWPYFKTYSYECSQGTLTCKGDNDA
CAAAVCDCDRLAAICFAGAPYNNNNYNIDLKARCQ
>N.atra_2221_1826_2267_2614_1861_2.2406_PLA2
MNLYQFKNMIKCTVPSRSWWDFADYGCYCGRGGSGTPVDDLDRCCQVHDNCYNEAEKISGCWPYFKTYSYECSQGTLTCKGDNNA
CAASVCDCDRLAAICFAGAPYNDNNYNIDLKARCQ
>N.atra_2291_1.03394919476848_PLA2
NLYQFKNMIKCTVPSRSWWDFADYGCYCGRGGSGTPVDDLDRCCQVHDNCYDEAGKISGCWPYFKTYSYECSQGTLTCKGDNNAC
AASVCDCDRLAAICFAGAPYNDNNYNIDLKARCQ
>N.atra_2365_0.000209486766863178_PLA2
NLYQFKNMIQCTVPSRSWWDFADYGCYCGKGGSGTPVDDLDRCCQVHDNCYNEAEKISGCWPYFKTYSYECSQGTLTCKGGNNAC
AAAVCDCDRLAAICFAGAPYTDANYNIDLKARCQ
>N.atra_2501_2738_1849_1846_1812_1779_0.2338_PLA2
NLYQFKNMIQCTVPSRSWWNFADYGCYCGRGGSGTPVDDLDRCCQVHDNCYDEAEKISGCWPYFKTYSYECSQGTLTCKGGNNAC
AAAVCDCDRLAAICFAGAPYNNNNYNIDLKARCQ
>N.atra_2748_2581_2660_1773_1774_0.2230_PLA2
NLYQFKNMIQCTVPNRSWWDFADYGCYCGRGGSGTPVDDLDRCCQVHDNCYDEAEKISRCWPYFKTYSYECSQGTLTCKNGNNAC
AAAVCDCDRLAAICFAGAPYNNNNYNIDLKARCQ
>N.atra_2804_0.106163089123156_PLA2
NLYQFKNMIQCTVPSRSWCDFADYGCYCGKGGSGTPVDDLDRCCQVHDNCYNEAEKISGCWPYFKTYSYECSQGTLTCKGGNNACA
AAVCDCDRLAAICFAGAPYTDANYNIDLKARCQ
>N.atra_2935_2606_1814_0.1266_PLA2
NLYQFKNMIKCTVPSRSWWDFADYGCYCGRGGSGTPVDDLDRCCQVHDNCYNEAEKISKCWPFFKTYSYKCSQGTLTCKGGNNAC
AASVCDCDRLAAICFAGAPYNDNNYNIDLKARCQ
>N.haje.UGA_1903_0.0439098483642701_PLA2
LNLYQFKNMIHCTVPSRPWWHFADYGCYCGRGGKGTPVDDLDRCCQIHDNCYDEAGKISGCWPYFKVYAYECSQGALSCRGGKNK
CAASVCDCDRLAAICFARATYNDNNYNIDFNARCQ
>N.haje.UGA_1906_0.0439098483642701_PLA2
NLYQFKNMIHCTVPNRPWWHFANYGCYCGRGGKGTPVDDLDRCCQIHDKCYDEAEKISGCWPYIKTYTYESCQGTLTCKDGGKCAA
SVCDCDRVAANCFARATYNDKNYNIDFNARCQ
>N.kaouthia_1936_1935_2.3199_PLA2
LNLYQFKNMIQCTVPNRSWWDFADYGCYCGRGGSGTPVDDLDRCCQVHDNCYNEAEKISRCWPYFKTYSYECSQGTLTCKGDNDA
CAAAVCDCDRLAAICFAGAPYNNNNYNIDLKARCQ
>N.kaouthia_1938_1917_1943_1931_1.3280_PLA2
NLYQFKNMIQCTVPNRSWWDFADYGCYCGRGGSGTPVDDLDRCCQVHDNCYDEAEKISRCWPYFKTYSYECSQGTLTCKNGNNAC
AAAVCDCDRLAAICFAGAPYNNNNYNIDLKARCQ
>N.kaouthia_1962_0.395958174512529_PLA2
NLYQFKNMIQCTVPSRSWWNFADYGCYCGRGGSGTPVDDLDRCCQVHDNCYDEAEKISGCWPYFKTYSYECSQGTLTCKGGNNAC
AAAVCDCDRLAAICFAGAPYNNNNYNIDLKARCQ
>N.kaouthia_1992_0.395958174512529_PLA2

LNLYQFKNMIQCTVPSRSWWDFADYGCYCGRGGSGTPVDDLDRCCQVHDNCYNEAEKISGCWPYFKTYSYECSQGTLTCKGDNDA
CAAAVCDCDRLAAICFAGAPYNNNNYNIDLKARCQ
>N.kaouthia_2028_1985_0.4050_PLA2
LNLYQFKNMIQCTVPSRSWWDFADYGCYCGRGGSGTPVDDLDRCCQVHDNCYNEAEKISGCWPYFKTYSYECSQGTLTCKGGNNA
CAAAVCDCDRLAAICFAGAPYNNNNYNIDLKARCQ
>N.melanoleuca_1875_1710_1608_7.7707_PLA2
NLYQFKNMIQCTVPNRSWWHFANYGCYCGRGGSGTPVDDLDRCCQIHDNCYGEAEKISGCWPYIKTYTYESCQGTLTSCGANNKCA
ASVCDCDRVAANCFARATYNDKNYNIDFNARCQ
>N.melanoleuca_1933_1786_1808_2025_2511_1820_1826_2497_7.3519_PLA2
NLYQFKNMIHCTVPNRSWWHFANYGCYCGRGGSGTPVDDLDRCCQIHDNCYGEAEKISGCWPYIKTYTYDSCQGTLTSCGAANNC
AASVCDCDRVAANCFARAPYIDKNYNIDFNARCQ
>N.melanoleuca_2641_1713_1779_2166_1748_2417_1706_2264_2895_2976_2.0218_PLA2
LNLYQFKNMIQCTVPNRSWWHFADYGCYCGRGGSGTPVDDLDRCCQIHDNCYGEAEKISGCWPYIKTYTYECSQGTLTCSGANDKC
AASVCDCDRVAANCFARAPYIDKNYNIDFNARCQ
>N.melanoleuca_2841_1526_1716_0.7434_PLA2
NLYQFKNMIHCTVPNRPWWHFANYGCYCGRGGKGTPVDDLDRCCQIHDKCYDEAEKISGCWPYIKTYTYESCQGTLTCKDGGKCAA
SVCDCDRVAANCFARATYNDKNYNIDFNARCQ
>N.mossambica_1210_1102_1125_1292_10.3747_PLA2
LNLYQFKNMIHCTVPSRPWWHFADYGCYCGRGGKGTPVDDLDRCCQVHDNCYEKAGKMGCWPYLTLYKYKCSQGKLTCSGGNSK
CGAAVCNCDLVAANCFAGARYIDANYNINFKKRCQ
>N.mossambica_1223_1093_0.0180_PLA2
NLYQFKNMIHCTVPSRPWWHFADYGCYCGRGGKGTPVDDLDRCCQVHDNCYEKAGKMGCWPYFTLYKYKCSQGKLTCSGGNSKC
GAAVCNCDLVAANCFAGARYIDANYNINFKKRCQ
>N.mossambica_1296_1371_1364_1434_1322_1339_1043_1.7546_PLA2
LNLYQFKNMIHCTVPSRPWWHFADYGCYCGRGGTGTPVDDLDRCCQVHDNCYGEAEKLGCWPYLTLYKYECSQGKLTCSGGNNKC
QAAVCNCDLVAANCFAGARYIDANYNVNLKERCQ
>N.mossambica_1842_2249_1387_2278_1462_1062_1333_1413_1420_2090_4.4331_PLA2
LNLYQFKNMIHCTVPSRPWWHFADYGCYCGRGGTGTAVDDLDRCCQVHDNCYGEAEKLGCWPYLTLYKYECSQGKLTCSGGNNKC
QAAVCNCDLVAANCFAGAPYIDANYNVNLKERCQ
>N.mossambica_1851_2222_931_1483_1321_2417_1416_974_1057_0.4912_PLA2
NLYQFKNMIHCTVPSRPWWHFADYGCYCGRGGKGTAVDDLDRCCQVHDNCYGEAEKLGCWPYLTLYKYECSQGKLTCSGGNNKCE
AAVCNCDLVAANCFAGAPYIDANYNVNLKERCQ
>N.mossambica_2269_0.324915476505541_PLA2
NLYQFKNMIQCTVPNRSWWDFADYGCYCGRGGSGTPVDDLDRCCQVHDNCYDEAEKISRCWPYFKTYSYECSQGTLTCKNGNNAC
AAAVCDCDRLAAICFAGAPYNNNNYNIDLKARCQ
>N.mossambica_956_2283_1048_0.5086_PLA2
NLYQFKNMIHCTVPSRPWWHFADYGCYCGRGGKGTPVDDLDRCCQVHDNCYEKAGKMGCWPYFTLYKYKCSQGKLPCSGGNNKC
QAAVCNCDLVAANCFAGAPYIDANYNVNLKERCQ
>N.mossambica_976_0.0051078127118414_PLA2
NLYQFKNMIHCTVPSRPWWHFADYGCYCGRGGKGTAVDDLDRCCQVHDNCYGEAEKLGCWPYLTLYKYECSQGKLTCSGGNNKCA
AAVCNCDLVAANCFAGARYIDANYNINLKERCQ
>N.naja_1810_1899_0.2332_PLA2
NLYQFKNMIKCTVPSRSWWDFADYGCYCGRGGSGTPVDDLDRCCQVHDNCYDEAGKISGCWPYFKTYSYECSQGTLTCKGDNNAC
AASVCDCDRLAAICFAGAPYNDNNYNIDLKARCQ
>N.naja_1915_1922_1929_1973_1988_1954_1860_1834_1934_1935_1993_3003_1968_1971_2640_0.8417_PLA2
NLYQFKNMIKCTVPSRSWWDFADYGCYCGRGGSGTPVDDLDRCCQVHDNCYNEAEKISGCWPYFKTYSYECSQGTLTCKGDNNAC
AASVCDCDRLAAICFAGAPYNDNNYNIDLKARCQ
>N.naja_1932_1965_1974_1874_1933_0.3783_PLA2
NLYQFKNMIKCTVPSRSWWDFADYGCYCGRGGSGTPVDDLDRCCQVHDNCYNEAEKISKCWPFFKTYSYKCSQGTLTCKGGNNAC
AASVCDCDRLAAICFAGAPYNDNNYNIDLKARCQ
>N.naja_1936_0.0128141515563217_PLA2
NLYQFKNMIQCTVPSRSWWDFADYGCYCGRGGSGTPVDDLDRCCQVHDNCYNEAEKISGCWPYFKTYSYECSQGTLTCKGGNNAC
AAAVCDCDRLAAICFAGAPYNDNDYNINLKARC

>N.naja_1983_1878_0.0615_PLA2
NLYQFKNMIECTVPARSWWDFADYGCYCGGGGSGTPTDDLDRCCQVHDNCYNQAQEITGCRPKWKTYTYQCTQGTLTCKGRNNS
CAATTCDCDRLAAICFAGAPYNDTNYNIDLKARCQ
>N.naja_2013_0.0111412575811514_PLA2
NLYQFKNMIQCTVPSRSWWDFADYGCYCGKGGSGTPVDDLDRCCQVHDNCYNEAEKISGCWPYFKTYSYECSQGTLTCKGGNNAC
AAAVCDCDRLAAICFAGAPYTDANYNIDLKARCQ
>N.naja_2107_1914_0.0078_PLA2
NLYQFKNMIQCTVPNRSWWDFADYGCYCGRGGSGTPVDDLDRCCQVHDNCYDEAEKISRCWPYFKTYSYECSQGTLTCKNGNNAC
AAAVCDCDRLAAICFAGAPYNNNNYNIDLKARCQ
>N.naja_2188_0.00112695067314748_PLA2
LNLYQFKNMIQCTVPNRSWWDFADYGCYCGRGGSGTPVDDLDRCCQVHDNCYGEAEKISRCWPYFKTYSYECSQGTLTCKGGNDA
CAAAVCDCDRLAAICFAGAPYNDNNYNIDLKARCQ
>N.naja_2418_0.00735031660517128_PLA2
NIYQFKNMIECTVPARSWWDFADYGCYCGGGGSGTPTDDLDRCCQVHDNCYNQAQEITGCRPKWKTYTYQCTQGTLTCKGRNNA
CAATTCDCDRLAAICFAGAPYNDTNYNIDLKARCQ
>N.naja_3006_0.00847873475785663_PLA2
NTYQFKNMIQCTVPKRSWWDFADYGCYCGRGGSGTPIDDLDRCCQVHDNCYNSAREQGGCRPKQKTYSYECKAGTLSCSGSNNSC
AATVCDCDRLAAICFAGAPYNDNNYNIDLKARCQ
>N.nigricollis.NGA_1024_1008_PLA2_0.9623
NLYQFKNMIHCTVPSRPWWHFADYGCYCGRGGKGTPVDDLDRCCQVHDNCYEKAGKMGCWPYFTLYKYKCSQGKLTCSGGNSKC
GAAVCNCDLVAANCFAGARYIDANYNINFKKRCQ
>N.nigricollis.NGA_1065_1063_995_PLA2_1.5168
NLYQFKNMIHCTVPSRPWWHFADYGCYCGRGGKGTPVDDLDRCCQVHDNCYEKAGKMGCWPYLTLYKYKCSQGKLTCSGGNSKC
GAAVCNCDLVAANCFAGARYIDANYNINFKKRCQ
>N.nigricollis.NGA_1067_1021_PLA2_1.6333
LNLYQFKNMIHCTVPSRPWWHFADYGCYCGRGGKGTPVVDLDRCCQVHDNCYEKAGKMGCWPYLTLYKYKCSKGKLTCNGGNSK
CGAAVCNCDLVAANCFAGAPYINANYNINFKKRCQ
>N.nigricollis.NGA_1245_1324_1351_1349_1284_1292_1328_1330_1334_1313_1310_1312_1300_1244_PLA2_6.34
34
LNLYQFKNMIHCTVPSRPWWHFADYGCYCGRGGTGTPVDDLDRCCQVHDNCYGEAEKLGCWPYLTLYKYECSQGKLTCSGGNNKC
QAAVCNCDLVAANCFAGARYIDANYNVNLKERCQ
>N.nigricollis.NGA_1353_1345_PLA2_0.7817
NLYQFKNMIHCTVPSRPWWHFADYGCYCGRGGKGTAVDDLDRCCQVHDNCYGEAEKLGCWPYLTLYKYECSQGKLTCSGGNNKCA
AAVCNCDLVAANCFAGARYIDANYNINLKERCQ
>N.nigricollis.NGA_1357_1343_PLA2_0.8166
LNLYQFKNMIHCTVPSRPWWHFADYGCYCGRGGSGTPVDDLDRCCQVHDNCYGEAEKLGCWPYLTLYKYECSEGKLTCSGGNNKC
QAAVCNCDLVAANCFAGAPYIDSNYNVNLKERCQ
>N.nigricollis.NGA_1362_1420_1346_PLA2_1.9714
NLYQFKNMIHCTVPSRPWWHFADYGCYCGRGGKGTAVDDLDRCCQVHDNCYGEAEKLGCWPYLTLYKYECSQGKLTCSGGNNKCE
AAVCNCDLVAANCFAGAPYIDANYNVNLKERCQ
>N.nigricollis.NGA_1403_PLA2_2.9209285085977
NLYQFKNMIHCTVPSRPWWHFADYGCYCGRGGTGTAVDDLDRCCQVHDNCYGEAEKLGCWPYLTLYKYECSQGKLTCSGGNNKC
QAAVCNCDLVAANCFAGAPYIDANYNVNLKERCQ
>N.nigricollis.TGO_1082_1116_1331_1165_1102_1114_1128_1155_1164_1153_1096_1204_1140_1159_1566_966
_1455_1.2798_PLA2
NLYQFKNMIHCTVPSRPWWHFADYGCYCGRGGKGTAVDDLDRCCQVHDNCYGEAEKLGCWPYLTLYKYECSQGKLTCSGGNNKCE
AAVCNCDLVAANCFAGAPYIDANYNVNLKERCQ
>N.nigricollis.TGO_1137_4.19587863926498_PLA2
LYQFKNMIHCTVPSRPWWHFADYGCYCGRGGTGTPVDDLDRCCQVHDNCYGEAEKLGCWPYLTLYKYECSQGKLTCSGGNNKCQA
AVCNCDLVAANCFAGARYIDANYNVNLKERCQ
>N.nigricollis.TGO_1179_1287_1351_1127_1176_2.1814_PLA2
LNLYQFKNMIHCTVPSRPWWHFADYGCYCGRGGKGTPVDDLDRCCQVHDNCYEKAGKMGCWPYFTLYKYKCSQGKLPCSGGNNK
CQAAVCNCDLVAANCFAGAPYIDANYNVNLKERCQ

>N.nigricollis.TGO_1448_0.0721230424026042_PLA2
NLYQFKNMIKCTVPSRSWLDFANYGCYCGRGGSGTPVDDLDRCCQIHDNCYNEAGKISGCWPYFKTYSYECSQGTLTCKGDNNSCA
ASVCDCDRLAAICFAGAPYNNDNYNINLKARCQ
>N.nigricollis.TGO_1527_1217_1235_1513_1761_1611_2.1127_PLA2
LNLYQFKNMIHCTVPSRPWWHFADYGCYCGRGGTGTAVDDLDRCCQVHDNCYGEAEKLGCWPYLTLYKYECSQGKLTCSGGNNKC
QAAVCNCDLVAANCFAGAPYIDANYNVNLKERCQ
>N.nigricollis.TGO_818_1029_888_973_859_2.0691_PLA2
LNLYQFKNMIHCTVPSRPWWHFADYGCYCGRGGKGTPVVDLDRCCQVHDNCYEKAGKMGCWPYLTLYKYKCSKGKLTCNGGNSK
CGAAVCNCDLVAANCFAGAPYINANYNINFKKRCQ
>N.nigricollis.TGO_910_0.0815836842973994_PLA2
NLYQFKNMIHCTVPSRPWWHFADYGCYCGRGGKGTPVDDLDRCCQVHDNCYEKAGKMGCWPYFTLYKYKCSQGKLTCSGGNSKC
GAAVCNCDLVAANCFAGARYIDANYNINFKKRCQ
>N.nigricollis.TGO_912_0.1621942822401_PLA2
NLYQFKNMIHCTVPSRPWWHFADYGCYCGRGGKGTPVDDLDRCCQVHDNCYEKAGKMGCWPYLTLYKYKCSQGKLTCSGGNSKC
GAAVCNCDLVAANCFAGARYIDANYNINFKKRCQ
>N.nigricollis.TZA_1240_1654_5.3454_PLA2
LNLYQFKNMIHCTVPSRPWWHFADYGCYCGRGGTGTAVDDLDRCCQVHDNCYGEAEKLGCWPYLTLYKYECSQGKLTCSGGNNKC
QAAVCNCDLVAANCFAGAPYIDANYNVNLKERCQ
>N.nigricollis.TZA_1242_1180_2.2749_PLA2
LNLYQFKNMIHCTVPSRPWWHFADYGCYCGRGGKGTPVVDLDRCCQVHDNCYEKAGKMGCWPYLTLYKYKCSKGKLTCNGGNSK
CGAAVCNCDLVAANCFAGAPYINANYNINFKKRCQ
>N.nigricollis.TZA_1248_0.518541049538747_PLA2
NLYQFKNMIHCTVPSRPWWHFADYGCYCGRGGKGTPVDDLDRCCQVHDNCYEKAGKMGCWPYFTLYKYKCSQGKLTCSGGNSKC
GAAVCNCDLVAANCFAGARYIDANYNINFKKRCQ
>N.nigricollis.TZA_1270_1209_0.9197_PLA2
NLYQFKNMIHCTVPSRPWWHFADYGCYCGRGGKGTPVDDLDRCCQVHDNCYEKAGKMGCWPYLTLYKYKCSQGKLTCSGGNSKC
GAAVCNCDLVAANCFAGARYIDANYNINFKKRCQ
>N.nigricollis.TZA_1558_1618_2.2967_PLA2
NLYQFKNMIHCTVPSRPWWHFADYGCYCGRGGKGTAVDDLDRCCQVHDNCYGEAEKLGCWPYLTLYKYECSQGKLTCSGGNNKCE
AAVCNCDLVAANCFAGAPYIDANYNVNLKERCQ
>N.nigricollis.TZA_2943_1563_1827_1602_1887_1536_1579_2304_1.7267_PLA2
LNLYQFKNMIHCTVPSRPWWHFADYGCYCGRGGTGTPVDDLDRCCQVHDNCYGEAEKLGCWPYLTLYKYECSQGKLTCSGGNNKC
QAAVCNCDLVAANCFAGARYIDANYNVNLKERCQ
>N.nivea_1685_0.000355849507647815_PLA2
NLYQFKNMIHCTVPNRPWWHFANYGCYCGRGGKGTPVDDLDRCCQIHDKCYDEAEKISGCWPYIKTYTYESCQGTLTCKDGGKCAA
SVCDCDRVAANCFARATYNDKNYNIDFNARCQ
>N.nivea_1718_0.00947725791444073_PLA2
LNLYQFKNMIHCTVPSRPWWHFADYGCYCGRGGKGTPVDDLDRCCQIHDNCYDEAGKISGCWPYFKVYAYECSQGALSCRGGKNK
CAASVCDCDRLAAICFARATYNDNNYNIDFNARCQ
>N.nubiae_1045_0.0181937927532329_PLA2
NLYQFKNMIHCTVPSRPWWHFADYGCYCGRGGKGTAVDDLDRCCQVHDNCYGEAEKLGCWPYLTLYKYECSQGKLTCSGGNNKCE
AAVCNCDLVAANCFAGAPYIDANYNVNLKERCQ
>N.nubiae_1061_1102_1149_1076_1069_1310_6.0035_PLA2
NLYQFKNMIHCTVPSRPWWHFADYGCYCGRGGKGTPIDDLDRCCQVHDNCYEKAGKMGCWPYFTLYKYKCSKGTLTCNGRNGKC
AAAVCNCDLVAANCFAGAPYINANYNIDFKKRCQ
>N.nubiae_1207_3.48328260147831_PLA2
LNLYQFKNCHCTVPSRPWWHFADYGCYCGRGGKGTPIDDLDRCCQVHDNCYEKAGKMGCWPYFTLYKYKCSKGTLTCNGRNGKCA
AAVCNCDLVAANCFAGAPYINANYNIDFKKRCQ
>N.nubiae_1329_1344_1369_2119_1347_1385_1343_1476_1056_1350_2349_1327_1596_1916_2023_4.1215_PLA
2
LNLYQFKNMIHCTVPSRPWWHFADYGCYCGRGGSGTPVDDLDRCCQVHDNCYGEAEKLGCWPYLTLYKYECSEGKLTCSGGNNKC
QAAVCNCDLVAANCFAGAPYIDSNYNVNLKERCQ
>N.pallida_1032_1581_1016_1455_2.4544_PLA2

LNLYQFKNCHCTVPSRPWWHFADYGCYCGRGGKGTPIDDLDRCCQVHDNCYEKAGKMGCWPYFTLYKYKCSKGTLTCNGRNGKCA
AAVCNCDLVAANCFAGAPYINANYNIDFKKRCQ
>N.pallida_1069_2.31075864460376_PLA2
NLYQFKNMIHCTVPSRPWWHFADYGCYCGRGGKGTPVDDLDRCCQVHDNCYEKAGKMGCWPYFTLYKYKCSQGKLTCSGGNSKC
GAAVCNCDLVAANCFAGARYIDANYNINFKKRCQ
>N.pallida_1161_1153_2.1797_PLA2
LNLYQFKNMIHCTVPSRPWWHFADYGCYCGRGGSGKPVDDLDRCCQVHDNCYGKAEKLGCWPYLTLYKYECSQGKLTCSGGNNKC
QAAVCNCDLVAANCFAGAPYIDANYNVNLKERC
>N.pallida_1175_2061_1212_2111_1269_1157_2.5120_PLA2
LNLYQFKNMIHCTVPSRPWWHFADYGCYCGRGGKGTPVDDLDRCCQVHDNCYEKAGKMGCWPYFTLYKYKCSQGKLPCSGGNNK
CQAAVCNCDLVAANCFAGAPYIDANYNVNLKERCQ
>N.pallida_1179_1856_2078_1261_2.4713_PLA2
LNLYQFKNMIHCTVPSRPWWHFADYGCYCGRGGTGTAVDDLDRCCQVHDNCYGEAEKLGCWPYLTLYKYECSQGKLTCSGGNNKC
QAAVCNCDLVAANCFAGAPYIDANYNVNLKERCQ
>N.pallida_1603_1061_1289_1403_1058_1031_1115_1028_2.5363_PLA2
NLYQFKNMIHCTVPSRPWWHFADYGCYCGRGGKGTPIDDLDRCCQVHDNCYEKAGKMGCWPYFTLYKYKCSKGTLTCNGRNGKC
AAAVCNCDLVAANCFAGAPYINANYNIDFKKRCQ
>N.pallida_1675_1159_1249_888_981_1186_1174_2201_1042_883_886_1714_3.7065_PLA2
NLYQFKNMIHCTVPSRPWWHFADYGCYCGRGGKGTAVDDLDRCCQVHDNCYGEAEKLGCWPYLTLYKYECSQGKLTCSGGNNKCE
AAVCNCDLVAANCFAGAPYIDANYNVNLKERCQ
>N.pallida_1720_0.063486821041223_PLA2
YQFKNMIQCTVPNRSWWDFADYGCYCGRGGSGTPVDDLDRCCQVHDNCYNEAEKISRCWPYFKTYSYECSQGTLTCKGDNDACAA
AVCDCDRLAAICFAGAPYNNNNYNIDLKARCQ
>N.pallida_1913_0.00111799930196328_PLA2
LNLYQFKNMIQCTVPSRSWWDFADYGCYCGRGGSGTPVDDLDRCCQVHDNCYNEAEKISGCWPYFKTYSYECSQGTLTCKGGNNA
CAAAVCDCDRLAAICFAGAPYNNNNYNIDLKARCQ
>N.pallida_2059_0.260628946185751_PLA2
NLYQFKNMIQCTVPSRSWWNFADYGCYCGRGGSGTPVDDLDRCCQVHDNCYDEAEKISGCWPYFKTYSYECSQGTLTCKGGNNAC
AAAVCDCDRLAAICFAGAPYNNNNYNIDLKARCQ
>N.pallida_2098_1538_1668_2073_9.3543_PLA2
NLYQFKNMIKCTVPSRSWLDFANYGCYCGRGGSGTPVDDLDRCCQIHDNCYNEAGKISGCWPYFKTYSYECSQGTLTCKGDNNSCA
ASVCDCDRLAAICFAGAPYNNDNYNINLKARCQ
>N.pallida_2121_0.260628946185751_PLA2
NLYQFKNMIQCTVPNRSWWDFADYGCYCGRGGSGTPVDDLDRCCQVHDNCYDEAEKISRCWPYFKTYSYECSQGTLTCKNGNNAC
AAAVCDCDRLAAICFAGAPYNNNNYNIDLKARCQ
>N.philippinensis_1827_2028_0.0282_PLA2
NLYQFKNMIQCTVPSRSWWNFADYGCYCGRGGSGTPVDDLDRCCQVHDNCYDEAEKISGCWPYFKTYSYECSQGTLTCKGGNNAC
AAAVCDCDRLAAICFAGAPYNNNNYNIDLKARCQ
>N.philippinensis_1848_0.553018447724255_PLA2
NTYQFKNMIQCTVPKRSWWDFADYGCYCGRGGSGTPIDDLDRCCQVHDNCYNSAREQGGCRPKQKTYSYECKAGTLSCSGSNNSC
AATVCDCDRLAAICFAGAPYNDNNYNIDLKARCQ
>N.philippinensis_1887_1908_2109_1.1061_PLA2
LNLYQFKNMIQCTVPNRSWWHFADYGCYCGRGGSGTPVDDLDRCCQIHDNCYNEAEKISRCWPYFKTYSYECSQGTLTCKGGNNAC
AAAVCDCDRLAAICFAGAPYNDNNYNIDLKARCQ
>N.philippinensis_1894_1868_1.1060_PLA2
LNLYQFKNMIQCTVPNRSWWDFADYGCYCGRGGSGTPVDDLDRCCQVHDNCYGEAEKISRCWPYFKTYSYECSQGTLTCKGGNDA
CAAAVCDCDRLAAICFAGAPYNDNNYNIDLKARCQ
>N.philippinensis_1899_1890_1.1060_PLA2
LNLYQFKNMVQCTVPNRSWWHFADYGCYCGRGGSGTPVDDLDRCCQIHDNCYNEAEKISRCWPYFKTYSYECSQGTLTCKGGNNA
CAAAVCDCDRLAAICFAGAPYNDNNYNIDLKARCQ
>N.philippinensis_1900_1914_0.5549_PLA2
NLYQFKNMIKCTVPSRSWWDFADYGCYCGRGGSGTPVDDLDRCCQVHDNCYNEAEKISKCWPFFKTYSYKCSQGTLTCKGGNNAC
AASVCDCDRLAAICFAGAPYNDNNYNIDLKARCQ

>N.philippinensis_1936_2022_0.5768_PLA2
LNLYQFKNMIQCTVPNRSWWDFADYGCYCGRGGSGTPVDDLDRCCQVHDNCYGEAEKISKCWPYFKTYSYECSQGTLTCKGGNNA
CAAAVCDCYRLAAICFAGAPYNNNNYNIDLKARCQ
>N.philippinensis_1937_1811_1912_1952_1959_1892_1883_2064_2.2426_PLA2
MNLYQFKNMIKCTVPSRSWWDFADYGCYCGRGGSGTPVDDLDRCCQVHDNCYNEAEKISGCWPYFKTYSYECSQGTLTCKGDNNA
CAASVCDCDRLAAICFAGAPYNDNNYNIDLKARCQ
>N.philippinensis_1988_0.553018447724255_PLA2
LNIYQFKNMIQCTVPSRSWWDFADYGCYCGRGGSGTPVDDLDRCCQVHDNCYNQAQEITGCRPKWKTYTYECSQGTLTCKGRNNA
CAATVCDCDRLAAICFAGAPYNDNNYNIDLKARCQ
>N.philippinensis_2029_1945_0.5768_PLA2
LNLYQFKNMIQCTVPSRSWWDFADYGCYCGRGGSGTPVDDLDRCCQVHDNCYNEAEKISGCWPYFKTYSYECSQGTLTCKGGNNA
CAAAVCDCDRLAAICFAGAPYNNNNYNIDLKARCQ
>N.philippinensis_2038_1850_2023_0.5898_PLA2
NLYQFKNMIQCTVPNRSWWDFADYGCYCGRGGSGTPVDDLDRCCQVHDNCYDEAEKISRCWPYFKTYSYECSQGTLTCKNGNNAC
AAAVCDCDRLAAICFAGAPYNNNNYNIDLKARCQ
>N.philippinensis_2061_1889_1944_1816_1.1108_PLA2
LNLYQFKNMVQCTVPNRSWWDFADYGCYCGRGGSGTPVDDLDRCCQVHDNCYGEAEKISRCWPYFKTYSYECSQGTLTCKGGNN
ACAAAVCDCDRLAAICFAGAPYNDNNYNIDLKARCQ
>N.philippinensis_2201_1907_0.5531_PLA2
NTYQFQNMIQCTVPKRSWRDFADYGCYCGRGGSGTPIDDLDSCCQVHDNCYNSAREQGGCRPKQKTYTYQCKAGGLSCSGANNSC
AATTCDCDRLAAICFAGAPYNDNNYNIDLKARCQ
>N.siamensis_1897_1842_1.6250_PLA2
LNLYQFKNMIQCTVPSRSWWDFADYGCYCGRGGSGTPVDDLDRCCQVHDNCYNEAEKISGCWPYFKTYSYECSQGTLTCKGDNDA
CAAAVCDCDRLAAICFAGAPYNNNNYNIDLKARCQ
>N.siamensis_1920_1.51719392624015_PLA2
NTYQFKNMIQCTVPKRSWWDFADYGCYCGRGGSGTPIDDLDRCCQVHDNCYNSAREQGGCRPKQKTYSYECKAGTLSCSGSNNSC
AATVCDCDRLAAICFAGAPYNDNNYNIDLKARCQ
>N.siamensis_2037_1703_1925_1939_1983_1986_1.5736_PLA2
NLYQFKNMIQCTVPSRSWWNFADYGCYCGRGGSGTPVDDLDRCCQVHDNCYDEAEKISGCWPYFKTYSYECSQGTLTCKGGNNAC
AAAVCDCDRLAAICFAGAPYNNNNYNIDLKARCQ
>N.siamensis_2039_1.51719392624015_PLA2
LNLYQFKNMIQCTVPSRSWWDFADYGCYCGRGGSGTPVDDLDRCCQVHDHCYNEAEKISGCWPYSKTYSYECSQGTLTCKGGNNA
CAAAVCDCDRLAAICFAGAPYNNNNYNIDLKARCQ
>N.siamensis_2090_1980_1.5245_PLA2
NLYQFKNMIQCTVPSRSWWDFADYGCYCGKGGSGTPVDDLDRCCQVHDNCYNEAEKISGCWPYFKTYSYECSQGTLTCKGGNNAC
AAAVCDCDRLAAICFAGAPYTDANYNIDLKARCQ
>N.siamensis_2198_1.51719392624015_PLA2
NLYQFKNMIECTVPARSWWDFADYGCYCGGGGSGTPTDDLDRCCQVHDNCYNQAQEITGCRPKWKTYTYQCTQGTLTCKGRNNS
CAATTCDCDRLAAICFAGAPYNDTNYNIDLKARCQ
>N.siamensis_2213_2040_1.5232_PLA2
NLYQFKNMIQCTVPSRSWCDFADYGCYCGKGGSGTPVDDLDRCCQVHDNCYNEAEKISGCWPYFKTYSYECSQGTLTCKGGNNACA
AAVCDCDRLAAICFAGAPYTDANYNIDLKARCQ
>N.siamensis_2481_1.51719392624015_PLA2
LNLYQFKNMIQCTVPNRSWWDFADYGCYCGRGGSGTPVDDLDRCCQVHDNCYGEAEKISRCWPYFKTYSYECSQGTLTCKGGNDA
CAAAVCDCDRLAAICFAGAPYNDNNYNIDLKARCQ
>N.siamensis_2617_2030_2462_1.5307_PLA2
LNLYQFKNMIQCTVPNRSWWDFADYGCYCGRGGSGTPVDDLDRCCQVHDNCYNEAEKISRCWPYFKTYSYECSQGTLTCKGDNDA
CAAAVCDCDRLAAICFAGAPYNNNNYNIDLKARCQ
>N.siamensis_2625_1.51719392624015_PLA2
NIYQFKNMIQCTVPSRSWWDFADYGCYCGRGGSGTPVDDLDRCCQVHDNCYNQAQEITGCRPKWKTYTYECSQGTLTCKGRNNA
CAATVCDCDRLAAICFAGAPYNDNNYNIDLKARCQ
>N.siamensis_2642_1.51719392624015_PLA2

NIYQFKNMIECTVPARSWWDFADYGCYCGGGGSGTPTDDLDRCCQVHDNCYNQAQEITGCRPKWKTYTYQCTQGTLTCKGRNNA
CAATTCDCDRLAAICFAGAPYNDTNYNIDLKARCQ
>N.siamensis_2667_2022_1.5239_PLA2
NLYQFKNMIKCTVPSRSWWDFADYGCYCGRGGSGTPVDDLDRCCQVHDNCYNEAEKISKCWPFFKTYSYKCSQGTLTCKGGNNAC
AASVCDCDRLAAICFAGAPYNDNNYNIDLKARCQ
>N.siamensis_2668_1.51719392624015_PLA2
NTYQFQNMIQCTVPKRSWRDFADYGCYCGRGGSGTPIDDLDSCCQVHDNCYNSAREQGGCRPKQKTYTYQCKAGGLSCSGANNSC
AATTCDCDRLAAICFAGAPYNDNNYNIDLKARCQ
>N.siamensis_2773_1.51719392624015_PLA2
LNLYQFKNMIQCTVPNRSWWHFADYGCYCGRGGSGTPVDDLDRCCQIHDNCYNEAEKISRCWPYFKTYSYECSQGTLTCKGGNNAC
AAAVCDCDRLAAICFAGAPYNDNNYNIDLKARCQ
>N.siamensis_2832_2711_3.0344_PLA2
LNLYQFKNMIQCTVPSRSWWDFADYGCYCGRGGSGTPVDDLDRCCQVHDNCYNEAEKISGCWPYFKTYSYECSQGTLTCKGGNNA
CAAAVCDCDRLAAICFAGAPYNNNNYNIDLKARCQ
>N.siamensis_2838_1.51719392624015_PLA2
YQFKNMVQCTVPNRSWWHFADYGCYCGRGGSGTPVDDLDRCCQIHDNCYNEAEKISRCWPYFKTYSYECSQGTLTCKGGNNACAA
AVCDCDRLAAICFAGAPYNDNNYNIDLKARCQ
>N.siamensis_2879_3115_1901_1904_2452_2592_2722_7.5919_PLA2
MNLYQFKNMIKCTVPSRSWWDFADYGCYCGRGGSGTPVDDLDRCCQVHDNCYNEAEKISGCWPYFKTYSYECSQGTLTCKGDNNA
CAASVCDCDRLAAICFAGAPYNDNNYNIDLKARCQ
>N.siamensis_2988_1.51719392624015_PLA2
LNLYQFKNMVQCTVPNRSWWDFADYGCYCGRGGSGTPVDDLDRCCQVHDNCYGEAEKISRCWPYFKTYSYECSQGTLTCKGGNN
ACAAAVCDCDRLAAICFAGAPYNDNNYNIDLKARCQ
>N.siamensis_2990_1931_1947_1955_1956_1.6460_PLA2
LNLYQFKNMIQCTVPNRSWWDFADYGCYCGRGGSGTPVDDLDRCCQVHDNCYGEAEKISKCWPYFKTYSYECSQGTLTCKGGNNA
CAAAVCDCYRLAAICFAGAPYNNNNYNIDLKARCQ
>N.siamensis_3030_2828_3.0344_PLA2
NLYQFKNMIKCTVPSRSWLDFANYGCYCGRGGSGTPVDDLDRCCQIHDNCYNEAGKISGCWPYFKTYSYECSQGTLTCKGDNNSCA
ASVCDCDRLAAICFAGAPYNNDNYNINLKARCQ
>N.siamensis_3325_1902_1907_1914_1915_1973_1976_1982_2031_2659_2771_1.7250_PLA2
NLYQFKNMIQCTVPNRSWWDFADYGCYCGRGGSGTPVDDLDRCCQVHDNCYDEAEKISRCWPYFKTYSYECSQGTLTCKNGNNAC
AAAVCDCDRLAAICFAGAPYNNNNYNIDLKARCQ
>N.sumatrana_1775_2225_1902_1984_3.2937_PLA2
LNLYQFKNMIQCTVPSRSWWDFADYGCYCGRGGSGTPVDDLDRCCQVHDHCYNEAEKISGCWPYSKTYSYECSQGTLTCKGGNNA
CAAAVCDCDRLAAICFAGAPYNNNNYNIDLKARCQ
>N.sumatrana_1884_1.62608206810383_PLA2
NTYQFQNMIQCTVPKRSWRDFADYGCYCGRGGSGTPIDDLDSCCQVHDNCYNSAREQGGCRPKQKTYTYQCKAGGLSCSGANNSC
AATTCDCDRLAAICFAGAPYNDNNYNIDLKARCQ
>N.sumatrana_1911_2119_3.2522_PLA2
LNLYQFKNMIQCTVPNRSWWDFADYGCYCGRGGSGTPVDDLDRCCQVHDNCYNEAEKISRCWPYFKTYSYECSQGTLTCKGDNDA
CAAAVCDCDRLAAICFAGAPYNNNNYNIDLKARCQ
>N.sumatrana_2043_2962_2128_2937_6.5043_PLA2
LNLYQFKNMIQCTVPSRSWWDFADYGCYCGRGGSGTPVDDLDRCCQVHDNCYNEAEKISGCWPYFKTYSYECSQGTLTCKGGNNA
CAAAVCDCDRLAAICFAGAPYNNNNYNIDLKARCQ
>N.sumatrana_2065_1.62608206810383_PLA2
NLYQFKNMIQCTVPSRSWCDFADYGCYCGKGGSGTPVDDLDRCCQVHDNCYNEAEKISGCWPYFKTYSYECSQGTLTCKGGNNACA
AAVCDCDRLAAICFAGAPYTDANYNIDLKARCQ
>N.sumatrana_2071_1.62608206810383_PLA2
NIYQFKNMIECTVPARSWWDFADYGCYCGGGGSGTPTDDLDRCCQVHDNCYNQAQEITGCRPKWKTYTYQCTQGTLTCKGRNNA
CAATTCDCDRLAAICFAGAPYNDTNYNIDLKARCQ
>N.sumatrana_2326_1.62608206810383_PLA2
NLYQFKNMIKCTVPSRSWWDFADYGCYCGRGGSGTPVDDLDRCCQVHDNCYNEAEKISKCWPFFKTYSYKCSQGTLTCKGGNNAC
AASVCDCDRLAAICFAGAPYNDNNYNIDLKARCQ

>N.sumatrana_2451_1.62608206810383_PLA2
NTYQFKNMIQCTVPKRSWWDFADYGCYCGRGGSGTPIDDLDRCCQVHDNCYNSAREQGGCRPKQKTYSYECKAGTLSCSGSNNSC
AATVCDCDRLAAICFAGAPYNDNNYNIDLKARCQ
>N.sumatrana_2752_2919_2498_2786_6.5043_PLA2
MNLYQFKNMIKCTVPSRSWWDFADYGCYCGRGGSGTPVDDLDRCCQVHDNCYNEAEKISGCWPYFKTYSYECSQGTLTCKGDNNA
CAASVCDCDRLAAICFAGAPYNDNNYNIDLKARCQ
>N.sumatrana_2863_1.62608206810383_PLA2
LNLYQFKNMIQCTVPSRSWWDFADYGCYCGRGGSGTPVDDLDRCCQVHDNCYNEAEKISGCWPYFKTYSYECSQGTLTCKGDNDA
CAAAVCDCDRLAAICFAGAPYNNNNYNIDLKARCQ
>N.sumatrana_2917_1.62608206810383_PLA2
NLYQFKNMIQCTVPSRSWWDFADYGCYCGKGGSGTPVDDLDRCCQVHDNCYNEAEKISGCWPYFKTYSYECSQGTLTCKGGNNAC
AAAVCDCDRLAAICFAGAPYTDANYNIDLKARCQ
>N.sumatrana_3031_1.62608206810383_PLA2
LNLYQFKNMIQCTVPNRSWWDFADYGCYCGRGGSGTPVDDLDRCCQVHDNCYGEAEKISRCWPYFKTYSYECSQGTLTCKGGNDA
CAAAVCDCDRLAAICFAGAPYNDNNYNIDLKARCQ
>N.sumatrana_3143_1.62608206810383_PLA2
NLYQFKNMVQCTVPNRSWWDFADYGCYCGRGGSGTPVDDLDRCCQVHDNCYGEAEKISRCWPYFKTYSYECSQGTLTCKGGNNA
CAAAVCDCDRLAAICFAGAPYNDNNYNIDLKARCQ
>N.sumatrana_3146_1.62608206810383_PLA2
NLYQFKNMIECTVPARSWWDFADYGCYCGGGGSGTPTDDLDRCCQVHDNCYNQAQEITGCRPKWKTYTYQCTQGTLTCKGRNNS
CAATTCDCDRLAAICFAGAPYNDTNYNIDLKARCQ
>N.sumatrana_3147_2381_3.2522_PLA2
NLYQFKNMIKCTVPSRSWLDFANYGCYCGRGGSGTPVDDLDRCCQIHDNCYNEAGKISGCWPYFKTYSYECSQGTLTCKGDNNSCA
ASVCDCDRLAAICFAGAPYNNDNYNINLKARCQ
>N.sumatrana_3212_1805_1857_1913_1919_1927_1985_2024_2747_1.7433_PLA2
NLYQFKNMIQCTVPNRSWWDFADYGCYCGRGGSGTPVDDLDRCCQVHDNCYDEAEKISRCWPYFKTYSYECSQGTLTCKNGNNAC
AAAVCDCDRLAAICFAGAPYNNNNYNIDLKARCQ
>N.sumatrana_3343_1810_1932_1979_3105_1775_1.7292_PLA2
NLYQFKNMIQCTVPSRSWWNFADYGCYCGRGGSGTPVDDLDRCCQVHDNCYDEAEKISGCWPYFKTYSYECSQGTLTCKGGNNAC
AAAVCDCDRLAAICFAGAPYNNNNYNIDLKARCQ
>N.sumatrana_3366_1915_1964_1978_1980_1987_1.7252_PLA2
LNLYQFKNMIQCTVPNRSWWDFADYGCYCGRGGSGTPVDDLDRCCQVHDNCYGEAEKISKCWPYFKTYSYECSQGTLTCKGGNNA
CAAAVCDCYRLAAICFAGAPYNNNNYNIDLKARCQ
>W.aegyptia_1685_0.00432440013949654_PLA2
NLYQFKNMIHCTVPSRPWWHFADYGCYCGRGGKGTPIDDLDRCCQVHDNCYEKAGKMGCWPYFTLYKYKCSKGTLTCNGRNGKC
AAAVCNCDLVAANCFAGAPYINANYNIDFKKRCQ
>W.aegyptia_1750_1754_0.0137_PLA2
NLYQFKNMIHCTVPSRPWWHFADYGCYCGRGGKGTAVDDLDRCCQVHDNCYGEAEKLGCWPYLTLYKYECSQGKLTCSGGNNKCE
AAVCNCDLVAANCFAGAPYIDANYNVNLKERCQ
>W.aegyptia_1753_0.0132511349568496_PLA2
LYQFKNMIHCTVPSRPWWHFADYGCYCGRGGTGTAVDDLDRCCQVHDNCYGEAEKLGCWPYLTLYKYECSQGKLTCSGGNNKCQA
AVCNCDLVAANCFAGAPYIDANYNVNLKERCQ
>W.aegyptia_2223_0.00707907316161679_PLA2
NLYQFKNMIQCTVPSRSWCDFADYGCYCGKGGSGTPVDDLDRCCQVHDNCYNEAEKISGCWPYFKTYSYECSQGTLTCKGGNNACA
AAVCDCDRLAAICFAGAPYTDANYNIDLKARCQ
>W.aegyptia_2227_0.00707907316161679_PLA2
LNIYQFKNMIQCTVPSRSWWDFADYGCYCGRGGSGTPVDDLDRCCQVHDNCYNQAQEITGCRPKWKTYTYECSQGTLTCKGRNNA
CAATVCDCDRLAAICFAGAPYNDNNYNIDLKARCQ
>W.aegyptia_2235_0.0114155398074315_PLA2
NLYQFKNMIQCTVPNRSWWHFANYGCYCGRGGSGTPVDDLDRCCQIHDNCYGEAEKISGCWPYIKTYTYESCQGTLTSCGANNKCA
ASVCDCDRVAANCFARATYNDKNYNIDFNARCQ
