# Supplementary material for: Virus-like particles displaying conserved toxin epitopes stimulate polyspecific, murine antibody responses capable of snake venom recognition
Source: Sci Rep. 2022 Jul 5;12:11328. doi: 10.1038/s41598-022-13376-x (PMC9256628; doi:10.1038/s41598-022-13376-x)

Supplementary File S6 – Raw western blot images for individual mice sera, to detect recognition of elapid venoms. Venoms used are: 1 *B. candidus,* 2 *D. polylepis,* 3 *N. kaouthia,* 4 *N. subfulva,* 5 *N. nigricollis* and 6 *O. scutellatus.* Blots were scanned at 700nm and 800 nm for 2 minutes in each channel. Naïve sera comparator was from male CD1 mice (unimmunised), **and the same naive blot** was imaged multiple times to compare veVLP sera against, as indicated by the same title above the blot (i.e naïve1, naïve 2, etc, where the number represents different naïve blots performed on different days, due to the several days required to analyse sera from all individual animals). Multiple gels were used to compare each group (shown here on separate rows), and the individual gels are indicated by horizontal black dividing lines and/or white spaces. Blots for each animal within one immunisation group were processed in parallel.

CTx_C (Grp. A)

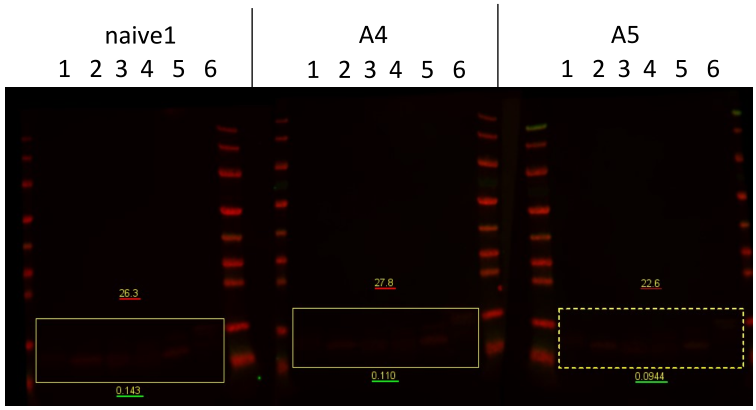

CTx_C
No Adjuvant
(Grp.M)

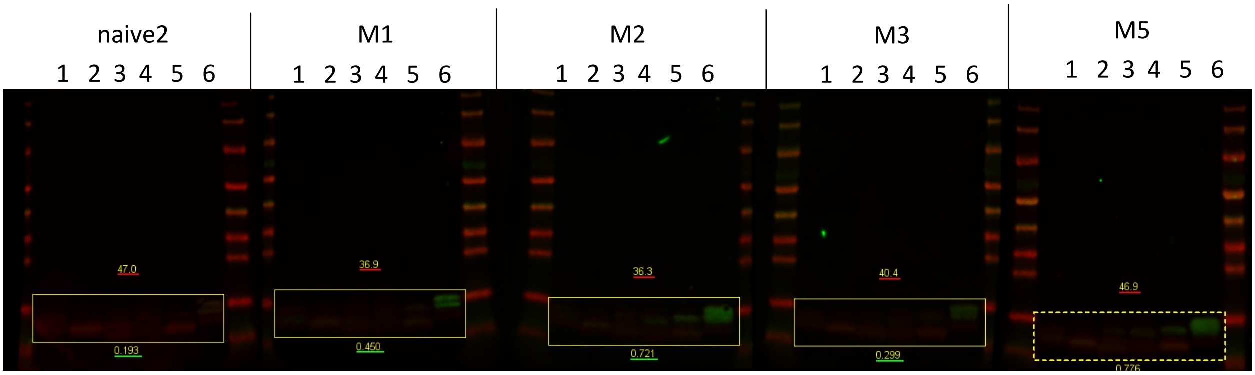

CTx_F (Grp. B)

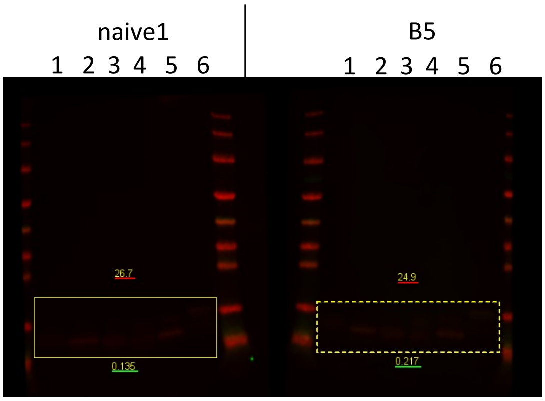

ATx_C
(Grp. C)

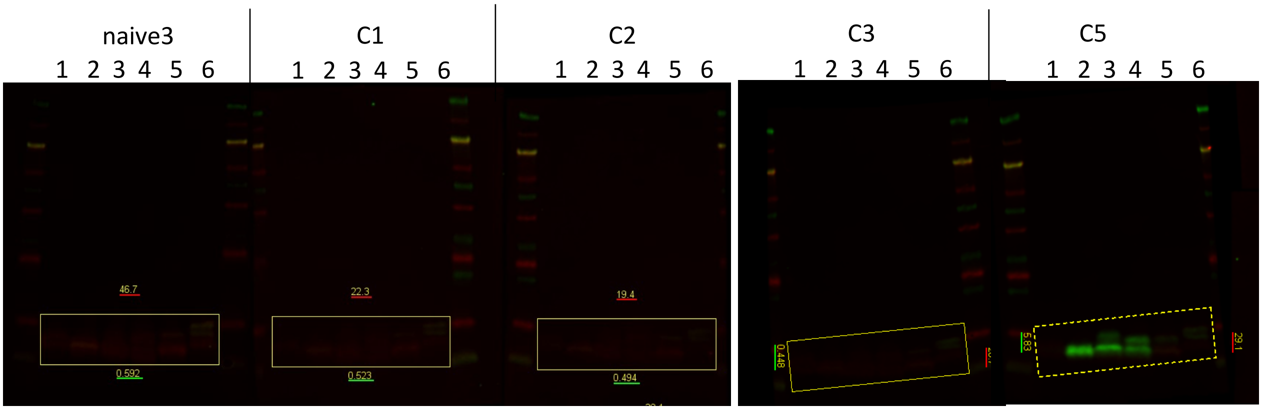

Supplementary File S6 –continued

naive4 1 2 3 4 5 6 | D1 1 2 3 4 5 6 | D3 1 2 3 4 5 6 | D5 1 2 3 4 5 6

ATx_F (Grp. D)

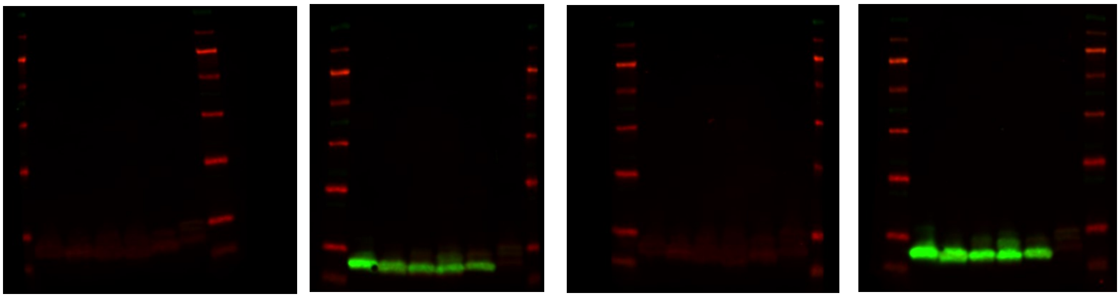

naive4 1 2 3 4 5 | E1 1 2 3 4 5 | E2 1 2 3 4 5

sNTx_C (Grp. E)

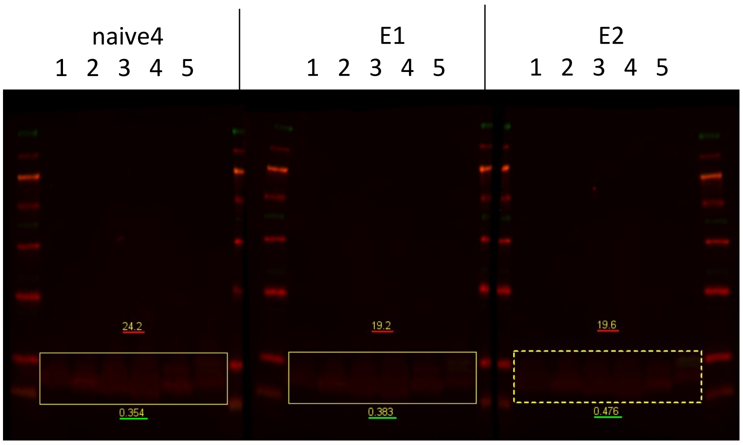

naive3 1 2 3 4 5 6 | F1 1 2 3 4 5 6 | F2 1 2 3 4 5 6 | F3 1 2 3 4 5 6 | F5 1 2 3 4 5 6

sNTx_F1 (Grp. F)

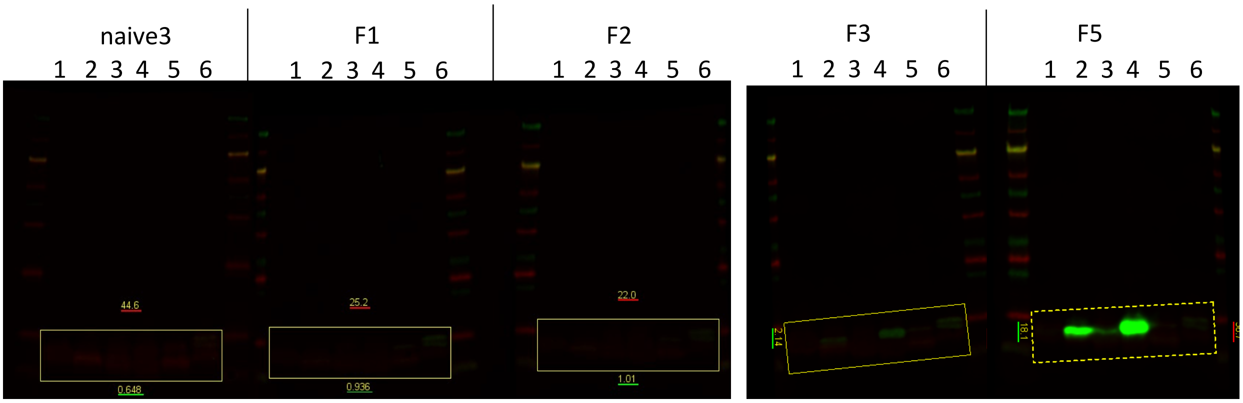

naive 1 2 3 4 5 6 | G1 1 2 3 4 5 6 | G2 1 2 3 4 5 6 | G3 1 2 3 4 5 6 | G4 1 2 3 4 5 6 | G5 1 2 3 4 5 6

sNTx_F2 (Grp. G)

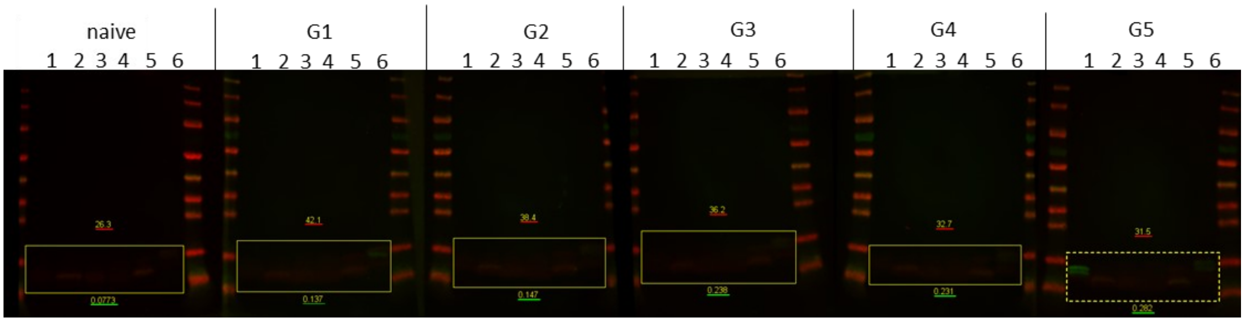

Supplementary File S6 –continued

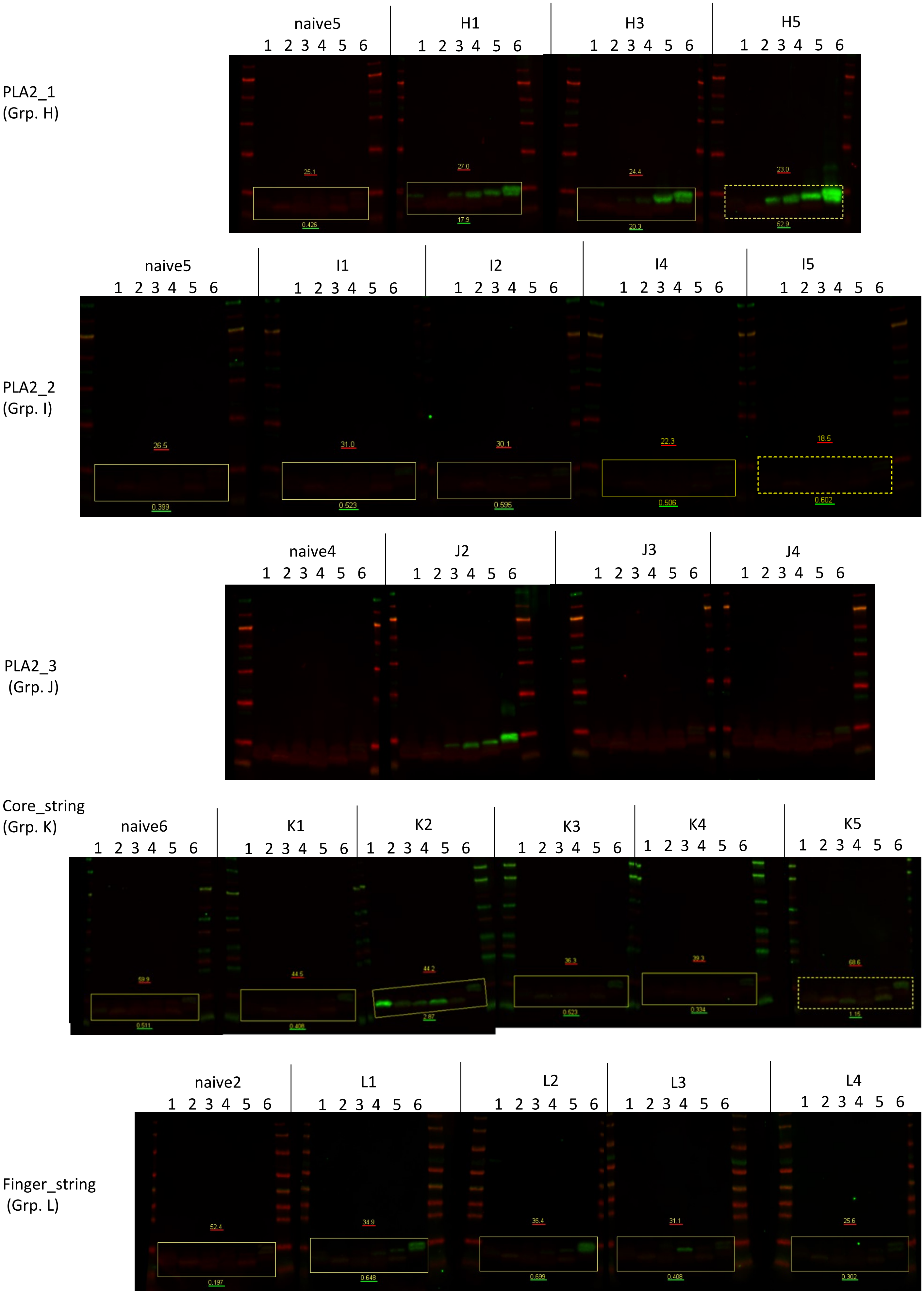

Supplement: Supplementary file 6 — Supplementary Information 6. [file 41598_2022_13376_MOESM6_ESM.pdf]
