# Supplementary material for: Virus-like particles displaying conserved toxin epitopes stimulate polyspecific, murine antibody responses capable of snake venom recognition
Source: Sci Rep. 2022 Jul 5;12:11328. doi: 10.1038/s41598-022-13376-x (PMC9256628; doi:10.1038/s41598-022-13376-x)

Supplementary File S7 – Recognition of purified neurotoxins by individual mice sera, to determine toxin specificity. Raw western blot images for individual mice sera or controls are shown, and the normalised data is shown in the histogram (expressed as fold-difference over naïve control sera). Toxins used were: MTx3 - muscarinic toxin 3 from *D. angusticeps*, bought from Alomone Labs (Jerusalem, Israel), Cyt3FTx1 – cytotoxic 3FTx from *N. haje,* sc3FTx – short chain 3FTx from *N. haje* and bPLA$_2$ – basic PLA$_2$ from *N. nigricollis*. Blots were scanned at 700 nm and 800 nm for 2 minutes in each channel. Naïve sera comparator was from male CD1 sera (unimmunised), and the same blot was imaged multiple times to compare veVLP sera against, as indicated by the same title above the blot (i.e naïve1, naïve 2, where the number represents different naïve blots performed on different days, due to the processing of these sera on different days). Multiple gels were used to compare each group (shown here on separate rows), and the individual gels are indicated by horizontal black dividing lines and/or white soaces. Blots in the same figure were processed in parallel.

Anti-cyt3FTx sera

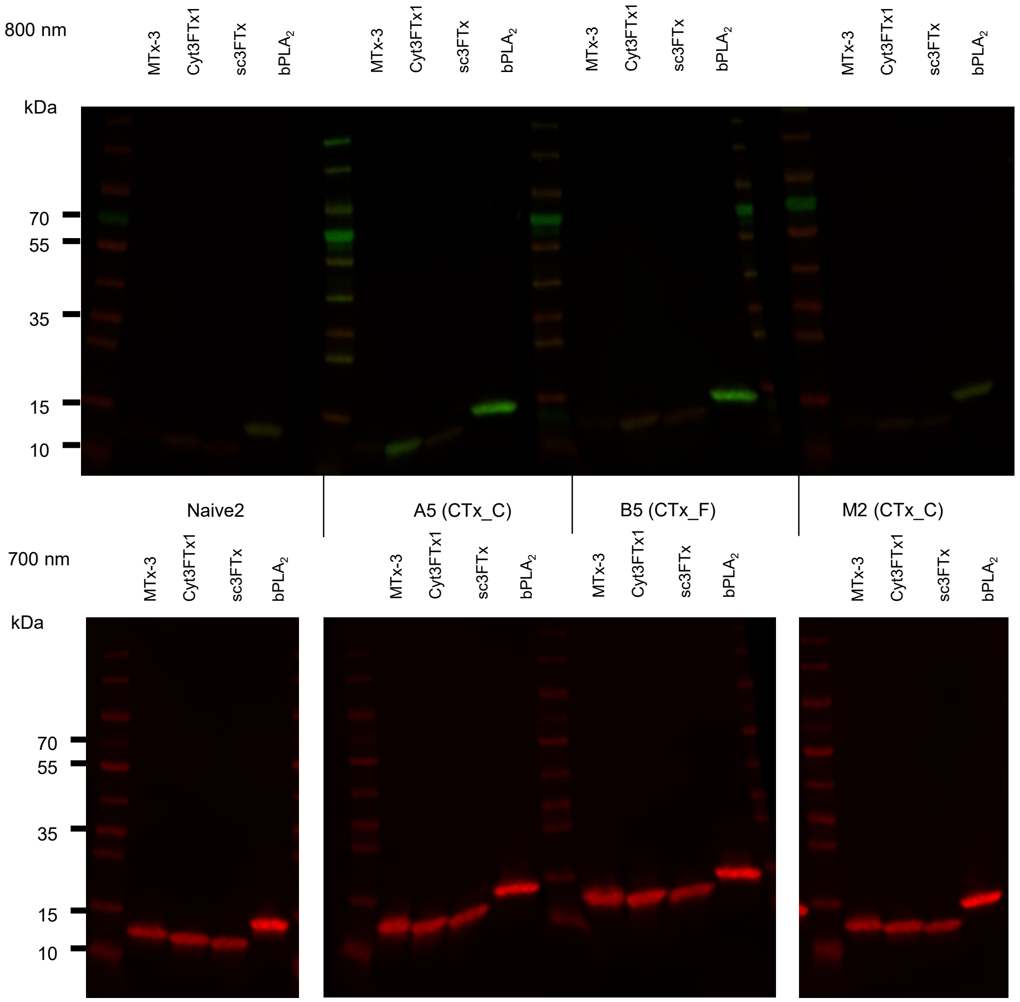

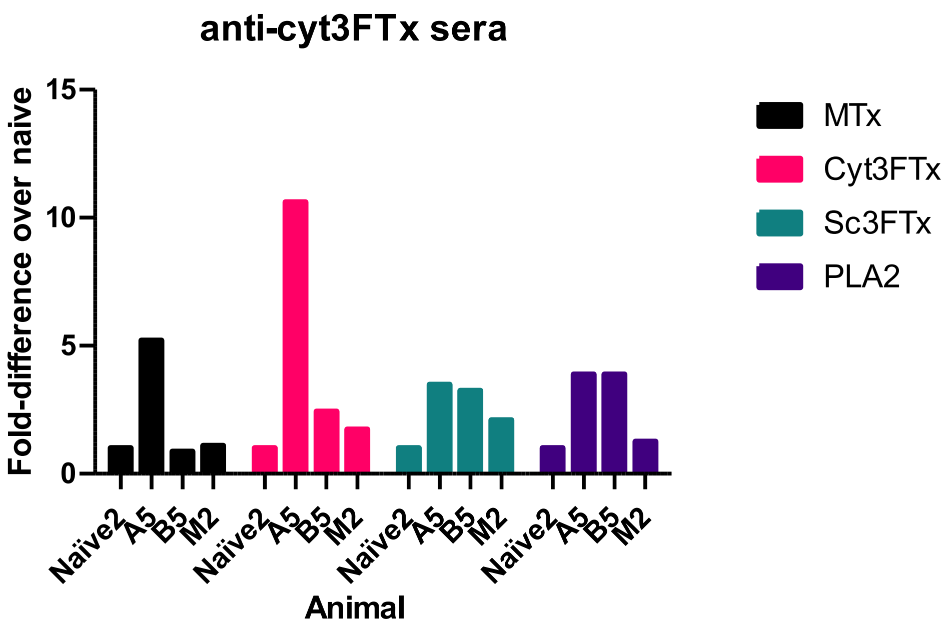

Anti-ATx sera

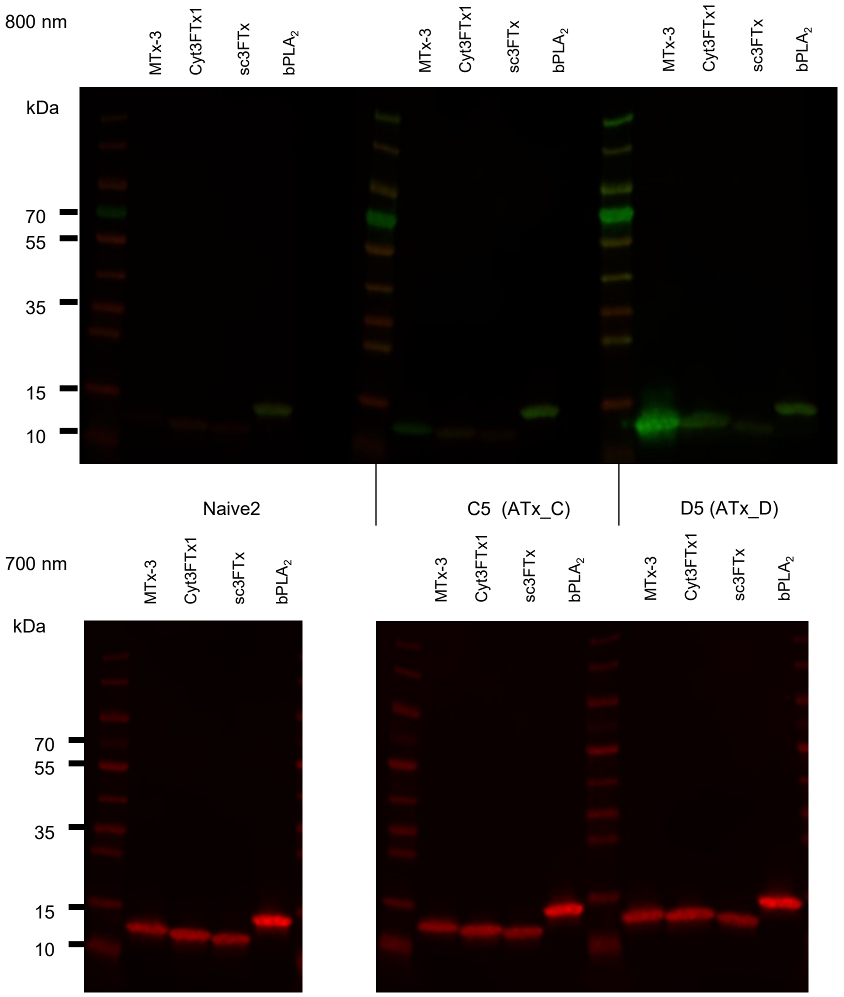

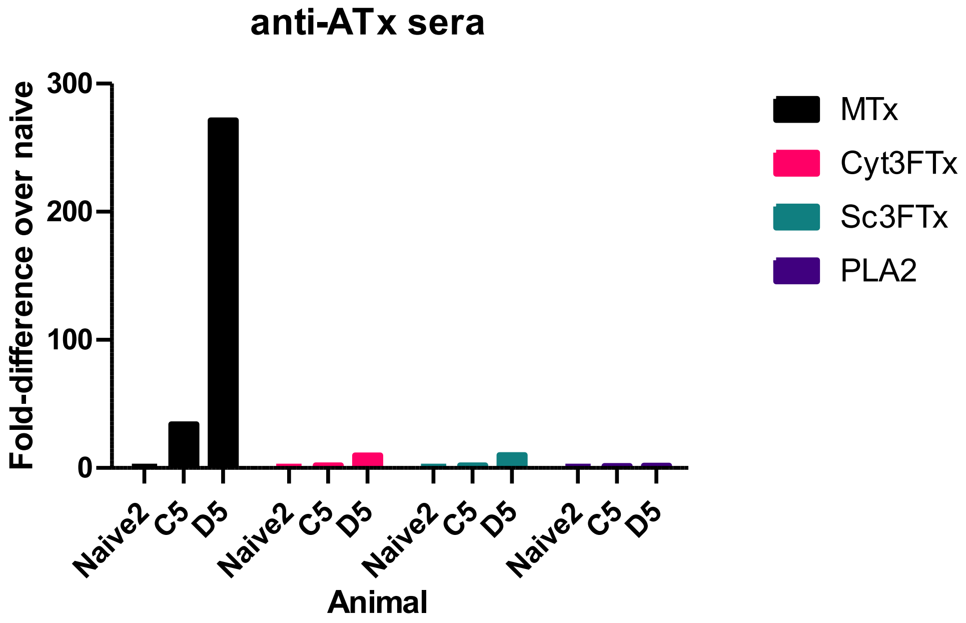

Anti-scNTx sera

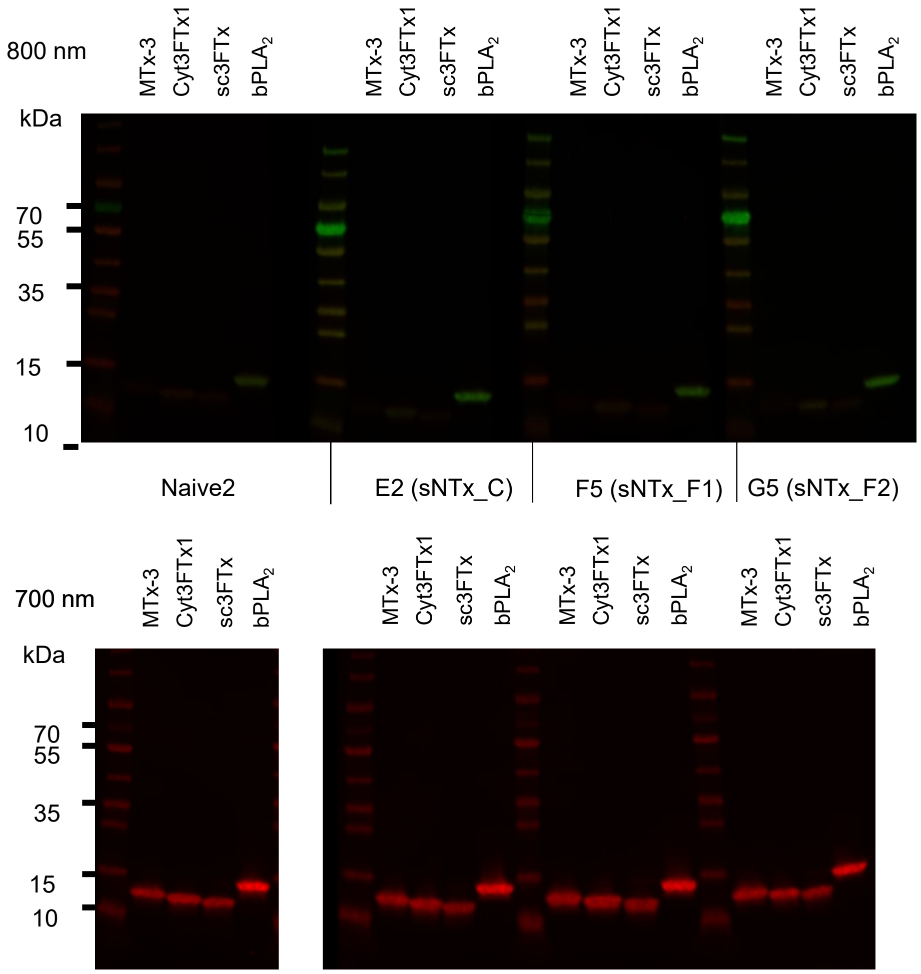

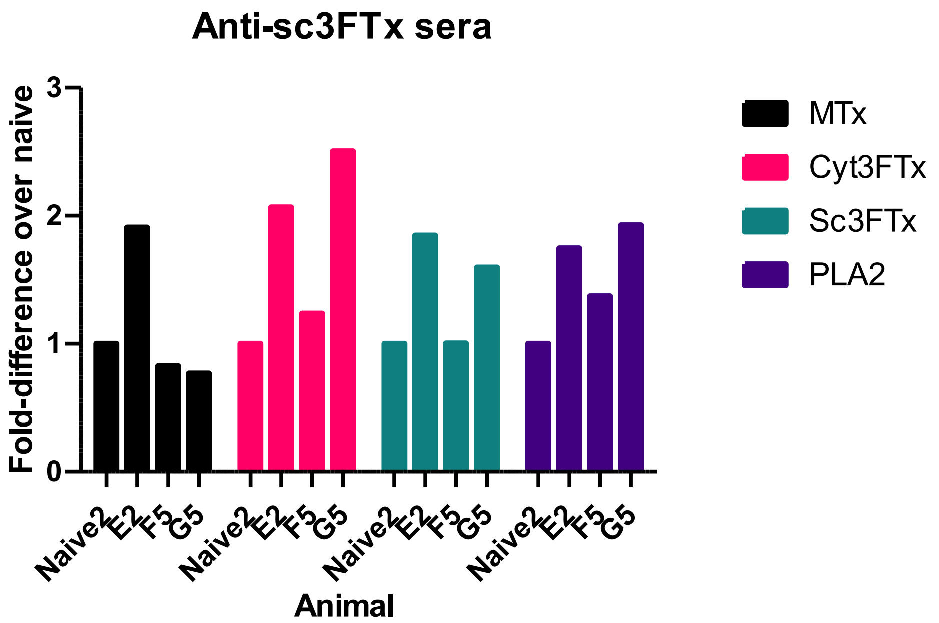

Anti-PLA_2 sera

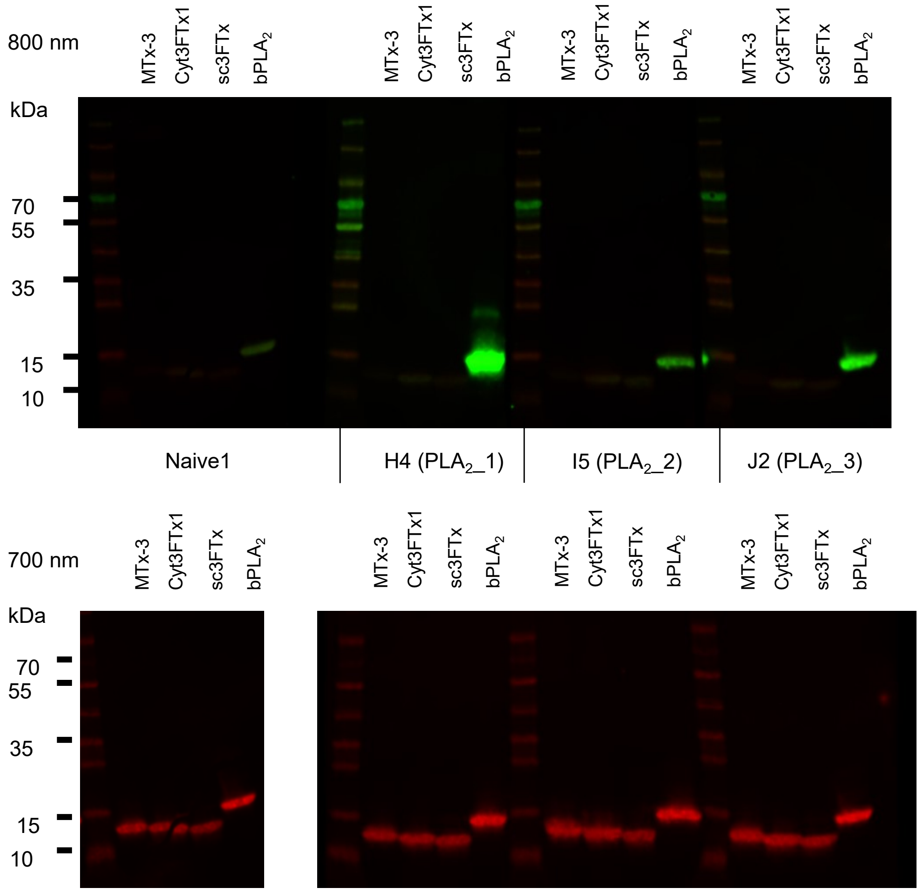

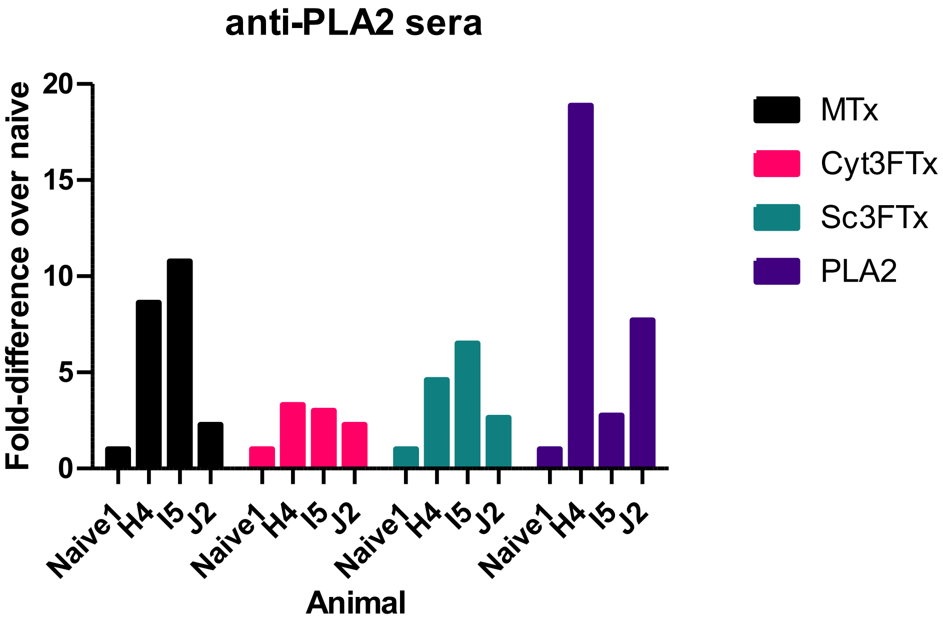

Anti-3FTx string sera

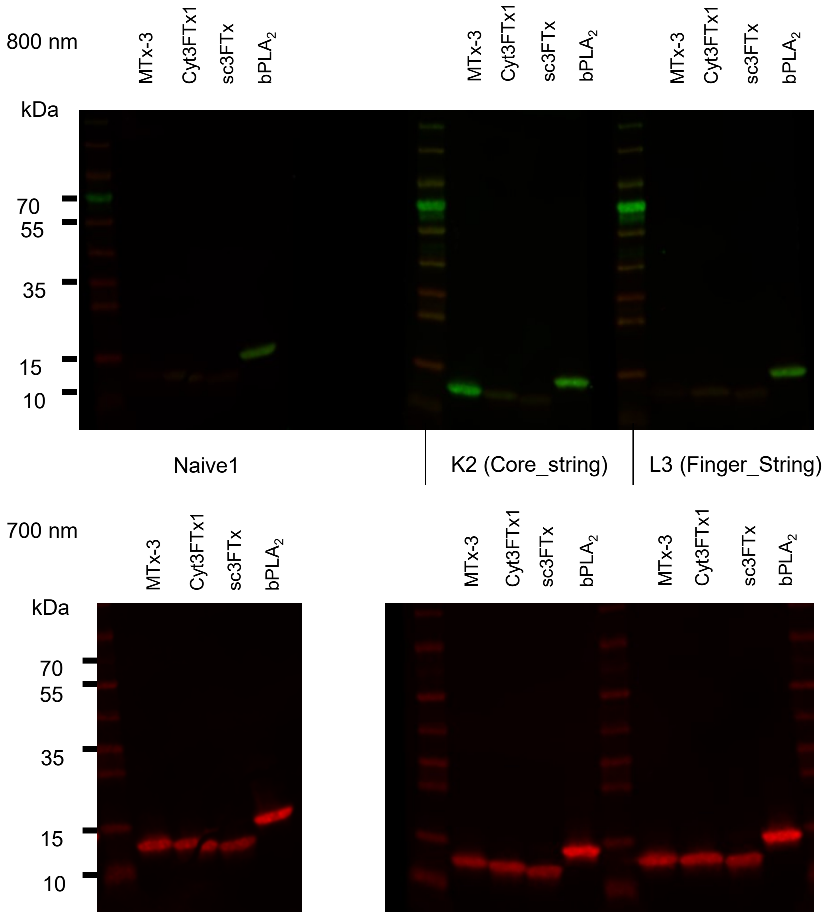

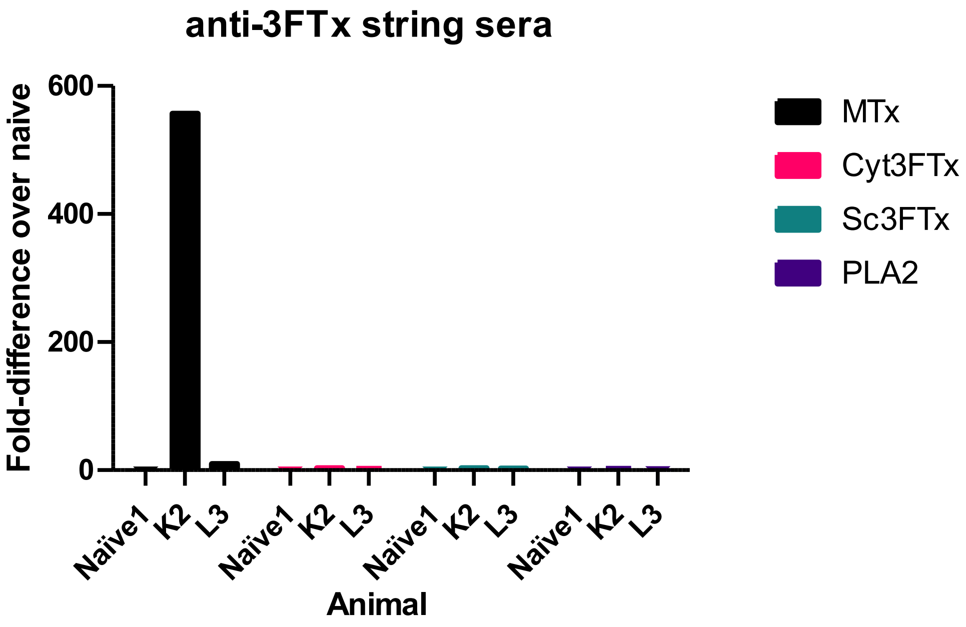

Supplement: Supplementary file 7 — Supplementary Information 7. [file 41598_2022_13376_MOESM7_ESM.pdf]
