# Supplementary material for: Virus-like particles displaying conserved toxin epitopes stimulate polyspecific, murine antibody responses capable of snake venom recognition
Source: Sci Rep. 2022 Jul 5;12:11328. doi: 10.1038/s41598-022-13376-x (PMC9256628; doi:10.1038/s41598-022-13376-x)
Supplement: Supplementary file 8 — Supplementary Information 8. [file 41598_2022_13376_MOESM8_ESM.pdf]

PDB ID	Entry	Entry name	Protein names	Organism	Length (AA)
1CB9	P01441	3SA2_NAJOX	Cytotoxin 2 (Cytotoxin II) (CTII)	*Naja oxiana*	60
1CDT	P01452	3SA4_NAJMO	Cytotoxin 4 (CTX M3) (Cardiotoxin VII4) (Cytotoxin V(II)4)	*Naja mossambica*	60
1FF4	P18328	3SIM2_DENAN	Muscarinic toxin 2 (MT2) (MTx2)	*Dendroaspis angusticeps*	86
1F94,1IJC	P81782	3NOJ_BUNCA	Bucandin	*Bungarus candidus*	63
1NOR	P01427	3S11_NAJOX	Short neurotoxin 1 (Neurotoxin II) (NT II) (NTII) (NTX II) (Neurotoxin alpha)	*Naja oxiana*	61
1NTN	P01382	3L21_NAJOX	Alpha-elapitoxin-Nno2a (Alpha-EPTX-Nno2a) (Long neurotoxin 1) (Neurotoxin I) (NT I) (Ntx-1) (Toxin I) (NnoI)	*Naja oxiana*	73
1NTX	P01416	3S11_DENPO	Short neurotoxin 1 (Neurotoxin alpha)	*Dendroaspis polylepis*	60
1ONJ	P80958	3S1CC_NAJAT	Cobrotoxin-b (CBT-b) (Atratoxin-b) (Cobrotoxin III) (CBT-III) (Cobrotoxin IV) (CBT IV) (NT3) (Short neurotoxin)	*Naja atra*	82
1RL5	P01451	3SA1_NAJOX	Cytotoxin 1 (Cytotoxin I) (CTI)	*Naja oxiana*	60
1TFS	P01414	3SL2_DENPO	Toxin FS-2 (FS2)	*Dendroaspis polylepis*	60
1TGX	P01468	3SA1_NAJPA	Cytotoxin 1 (CTX-1) (Cardiotoxin gamma)	*Naja pallida*	60
2CCX	P01467	3SA1_NAJMO	Cytotoxin 1 (CTX M1) (Cardiotoxin IIB) (CTX-IIB) (Cytotoxin V(II)1)	*Naja mossambica*	60
1DRS,2LA1	P28375	3SPM_DENJA	Dendroaspin (Glycoprotein IIb-IIIa antagonist) (Mambin) (Platelet aggregation inhibitor)	*Dendroaspis jamesoni kaimosae*	59
2MFA	P0DKS3	3SX2_DENPO	Mambalgin-2 (Ma-2) (Mamb-2) (Pi-Dp2)	*Dendroaspis polylepis*	57
2MJY	P0DKR6	3SX1_DENPO	Mambalgin-1 (Mamb-1) (Pi-Dp1)	*Dendroaspis polylepis*	78
3HH7	A8N286	3SO8_OPHHA	Haditoxin (Muscarinic toxin-like protein 3 homolog) (MTLP-3 homolog)	*Ophiophagus hannah*	86
1NEA,3NDS	P01426	3S11_NAJPA	Short neurotoxin 1 (Neurotoxin alpha) (Toxin alpha)	*Naja pallida*	61
2VLW,3FEV,3NEQ	Q8QGR0	3SIM7_DENAN	Muscarinic toxin 7 (MT-7) (MT7) (Muscarinic toxin 1) (m1-toxin)	*Dendroaspis angusticeps*	86
3FEV,3NEQ,4DO8	P81030	3SIM1_DENAN	Muscarinic toxin 1 (MT1) (MTx1)	*Dendroaspis angusticeps*	66
4LFT	C0HJD7	3L24_DENPO	Alpha-elapitoxin-Dpp2d (Alpha-EPTX-Dpp2d)	*Dendroaspis polylepis*	72
4IYE,5MG9	P85092	3SI1A_DENAN	Toxin AdTx1 (Rho-elapitoxin-Da1a) (Rho-Da1a) (Rho-EPTX-Da1a)	*Dendroaspis angusticeps*	65

Table S1: Details of PDB entries used in 3FTx epitope design.

Homology group	#seqs in group	Group descriptor
GR7	55	Type 1A cytotoxins
GR1	38	Aminergic type toxins
GR17	36	Type I alpha-neurotoxins
GR10	28	Non-conventional toxins
GR13	19	Orphan group VIII
GR15	18	Type I alpha-neurotoxins
GR8	13	L-type calcium blockers
GR5	11	Weak neurotoxins
GR4	10	Orphan group I
GR9	9	Mambalgins
GR2	7	Synergistic type toxins
GR16	6	Type B muscarinic toxins
GR14	5	Antiplatelet toxins
GR6	2	Type II alpha-neurotoxins
GR19	2	Type I alpha-neurotoxins
GR3	1	Muscarinic type toxins
GR11	1	Orphan group XIX
GR12	1	Orphan group I
GR18	1	Weak neurotoxins
GR20	1	Orphan group II
GR21	1	Orphan group XII

Table S2: Identified homology groups with number of representative sequences and group descriptors

3FTx Group	Epitope	Epitope Length (AA)	GR1	GR10	GR11	GR12	GR13	GR15	GR16	GR17	GR18	GR19	GR3	GR4	GR5	GR6	GR7	GR8	GR9	Aminergic toxin	L-type calcium blocker	Orphan group I	Orphan group II	Orphan group IV	Orphan group IX	Orphan group VI	Orphan group VIII	Orphan group X	Orphan group XI	Orphan group XIX	Orphan group XV	Orphan group XX	Type C muscarinic toxin	Type I alpha-neurotoxin	Type IA cytotoxin	Type II alpha-neurotoxin	not stated
**GR1_c**	DCPDGQNLC	9	79	0	0	0	0	0	0	0	0	0	0	0	0	0	0	0	0	24	0	0	0	0	0	6	0	0	0	0	2	0	43	0	0	4	0
**GR1_f**	TRGCAATCP	9	##	0	0	0	0	0	0	0	0	0	0	0	0	0	0	0	0	12	0	0	45	3	0	0	0	0	0	0	0	0	39	0	0	1	0
**GR10_c**	CAKTCTEE	8	0	36	0	0	0	0	0	0	0	0	0	0	0	0	0	0	0	0	0	0	0	0	0	0	0	0	0	36	0	0	0	0	0	0	0
**GR10_f**	SGCHLKIT	8	0	28	0	0	0	0	0	0	0	0	0	0	0	0	0	0	0	0	0	0	0	0	0	0	0	0	0	28	0	0	0	0	0	0	0
**GR13_c1**	GCTFSCPE	8	0	0	0	0	39	0	0	0	0	0	0	0	0	0	0	0	0	0	0	0	0	0	0	0	36	0	0	0	0	1	0	0	0	0	2
**GR13_c2**	GCTFTCPE	8	0	0	0	0	52	0	0	0	0	0	0	0	0	0	0	0	0	8	0	0	0	4	0	0	39	0	0	0	0	0	0	0	0	1	0
**GR13_f**	TPETTEICP	9	0	0	0	0	72	0	0	0	0	0	0	0	0	0	0	0	0	0	0	17	0	0	0	0	40	0	0	0	0	0	15	0	0	0	0
**GR15_c1**	TKSCEENS	8	0	0	0	0	0	20	0	0	0	0	0	0	0	0	0	0	0	0	2	0	0	0	0	0	0	1	2	0	0	0	0	13	0	0	2
**GR15_c2**	TTSCGDYF	8	0	0	0	0	0	5	0	0	0	0	0	0	0	0	0	0	0	0	0	0	0	0	0	0	0	0	0	0	0	0	0	5	0	0	0
**GR15_f1**	TPATTKSC	8	0	0	0	0	0	22	0	0	0	0	0	0	0	0	0	0	0	0	0	0	0	0	0	0	0	0	0	0	0	0	0	22	0	0	0
**GR17_c**	CHNQQSSQ	8	0	0	0	0	0	0	0	100	0	0	0	0	0	0	0	0	0	0	0	0	0	0	0	0	0	0	0	0	0	0	0	100	0	0	0
**GR17_f1**	DHRGTIIE	8	0	0	0	0	0	0	0	100	0	0	0	0	0	0	0	0	0	0	0	0	0	0	0	0	0	0	0	0	0	0	0	100	0	0	0
**GR17_f2**	DHRGYRTE	8	0	0	0	0	0	0	0	89	0	0	0	0	0	0	0	0	0	0	0	0	0	0	0	0	0	0	0	0	0	0	0	89	0	0	0
**GR7_c**	TCPEGKNL	8	0	0	0	0	0	0	0	0	0	0	0	0	0	0	100	0	0	0	0	0	0	0	0	0	0	0	0	0	34	0	0	0	66	0	0
**GR7_f**	IDVCPKSSLL	10	0	0	0	0	0	0	0	0	0	0	0	0	0	0	100	0	0	0	0	0	0	0	0	0	0	0	0	0	0	0	0	0	100	0	0

Table S3 Frequency of individual similar 3FTx epitopes (minimum 7 AA alignment length, 80% identity, 80% coverage, mismatches ≤ 1) in expanded (1425 sequence) 3FTx data set. Data displayed as both vs. homology group and vs. UniProt subtype descriptors.

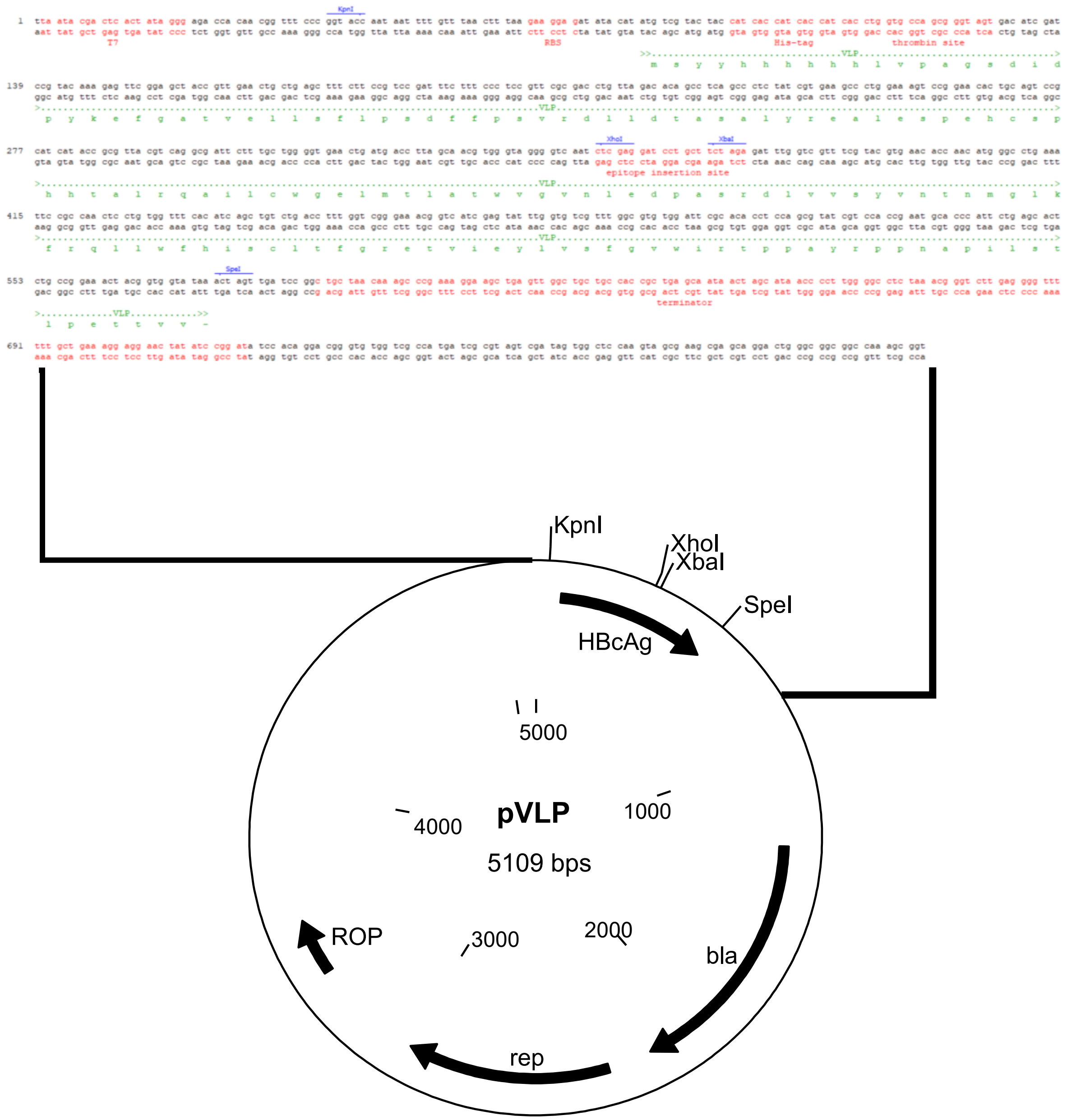

Figure S1. Plasmid map and sequence features of pVLP

A. GR7: Type 1A cytotoxins

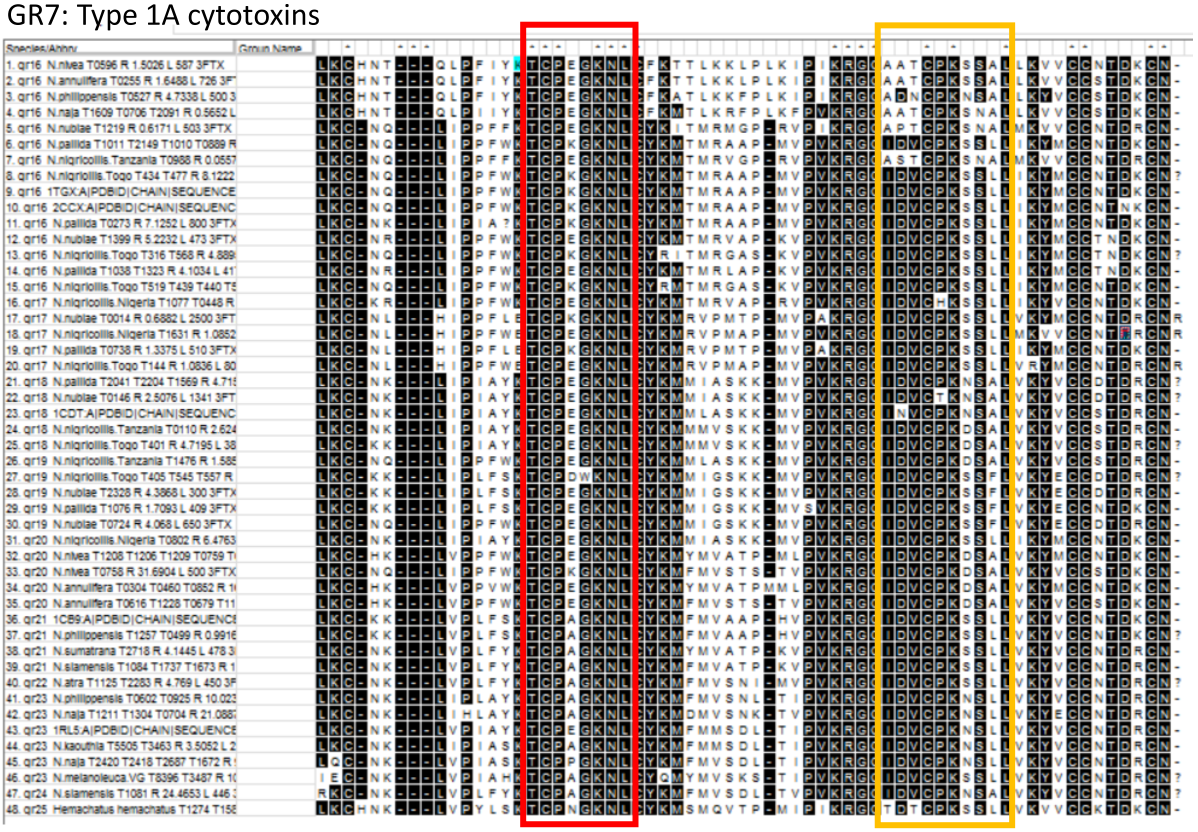

B. GR1: Aminergic toxins

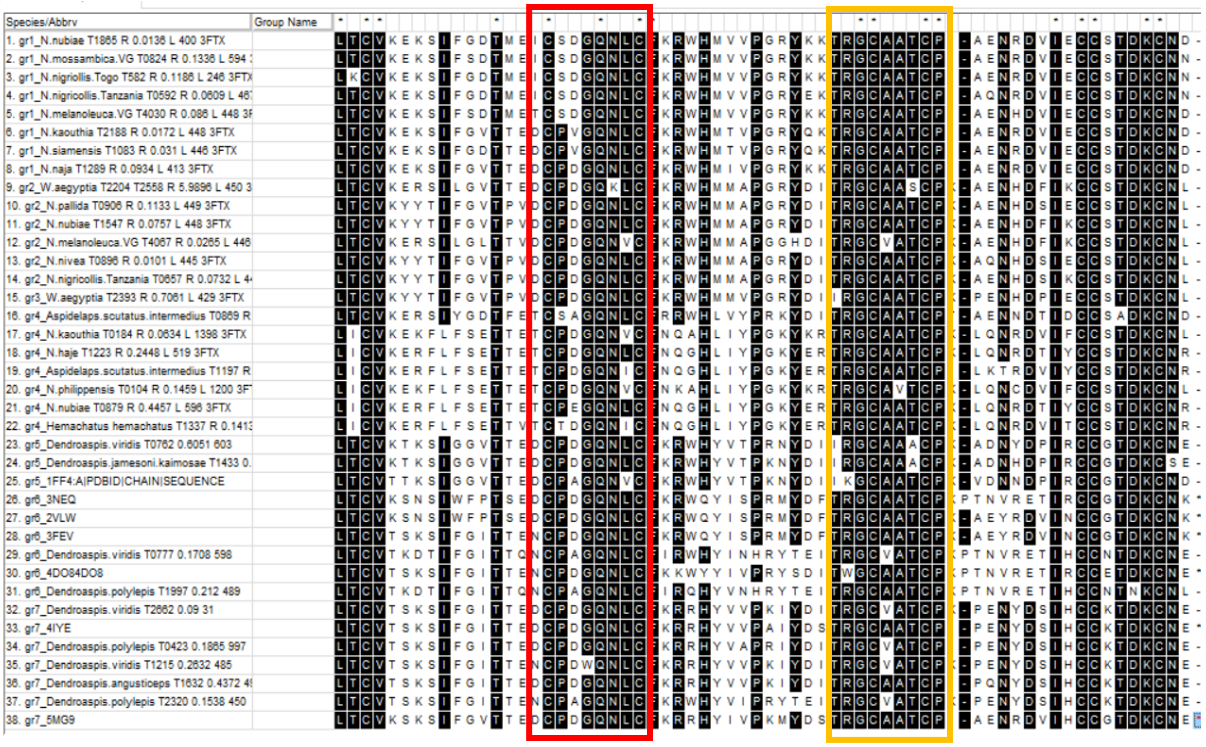

Figure S2. Alignments of A) GR7 Type 1A cytotoxins and B) GR1 Aminergic toxins. Black residues indicate 80% AA conservation. *= conserved. Red boxes indicate location of core epitopes, orange boxes indicate location of finger epitopes.

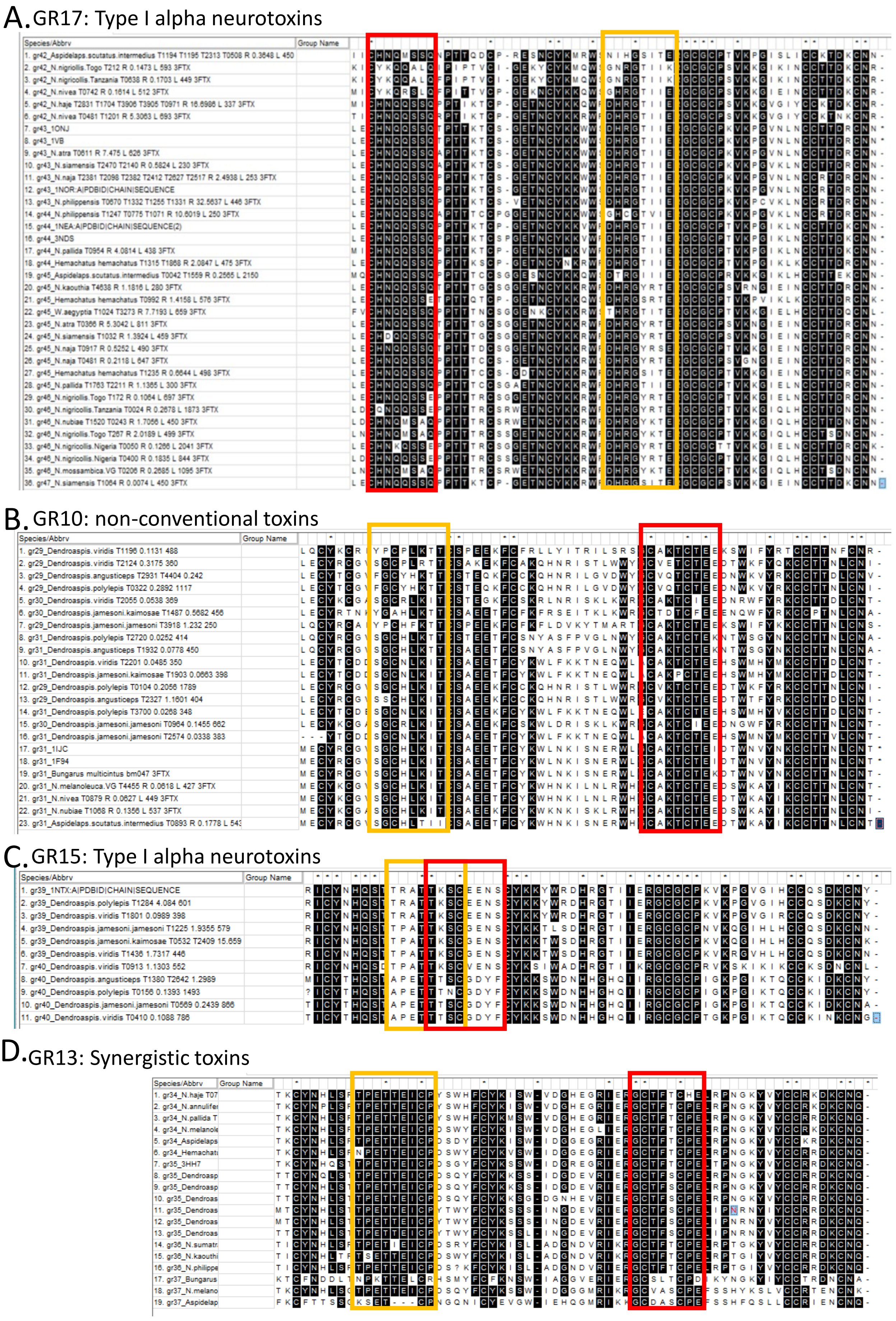

Figure S3. Alignments of A) GR17 Type I alpha-neurotoxins, B) GR10 non-conventional toxins, C) GR15 alternative Type I alpha neurotoxins and D) GR13 Synergistic toxins. Black residues indicate 80% AA conservation. *= conserved. Red boxes indicate location of core epitopes, orange boxes indicate location of finger epitopes.

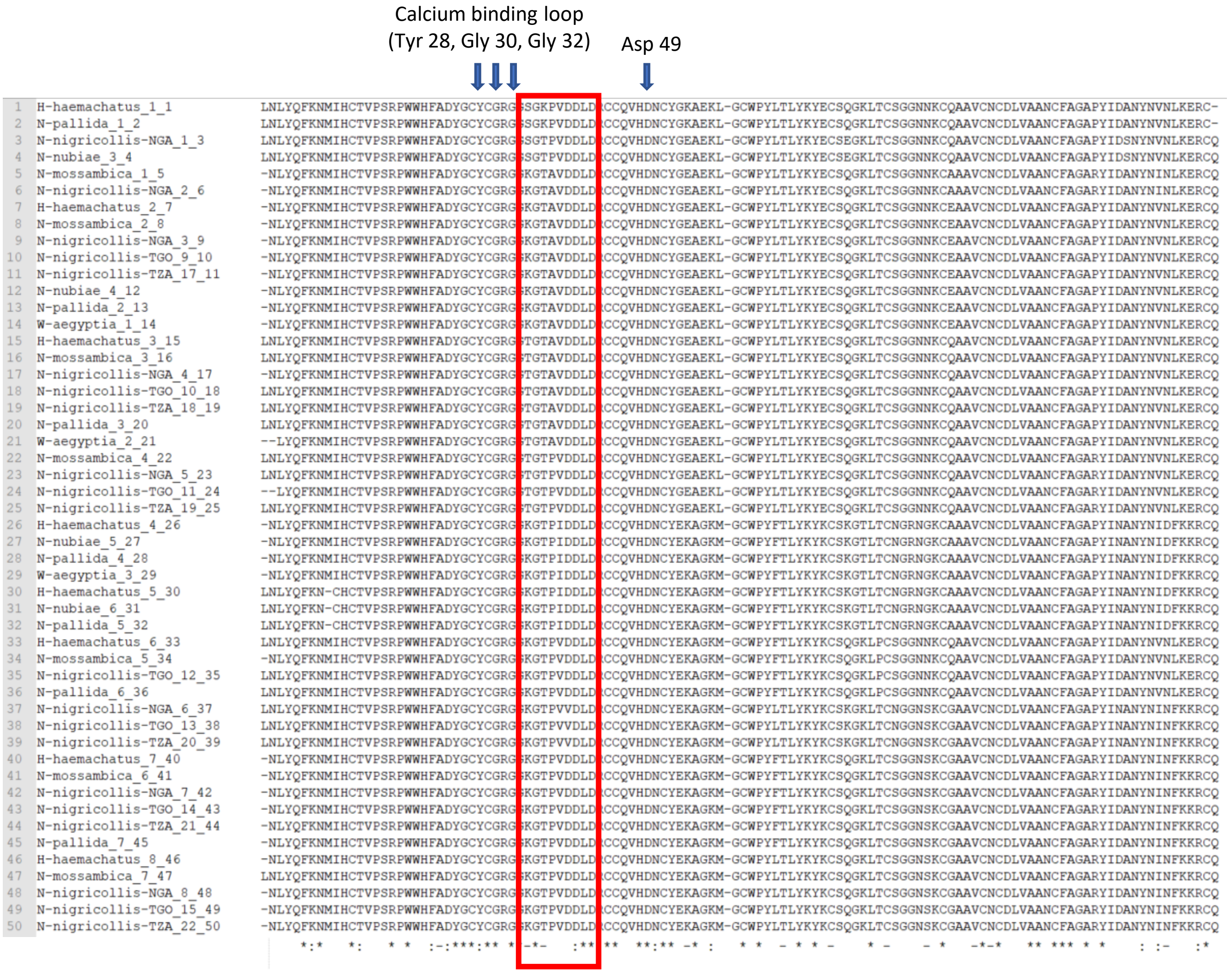

Figure S4. CLUSTAL Omega Alignments of Group I PLA_2 sequences used in this study (Supp. File S2). Note only the first 50 sequences are displayed. "*"= fully conserved residue, ":" = residues with strongly similar properties. "." = residues with weakly similar properties. Red boxes indicate location of the three individual PLA epitopes (PLA2_1, PLA2_2 & PLA2_3 – see manuscript table 1). Blue arrows represent positions of the calcium binding loop and Asp 49 required for calcium ion positioning.

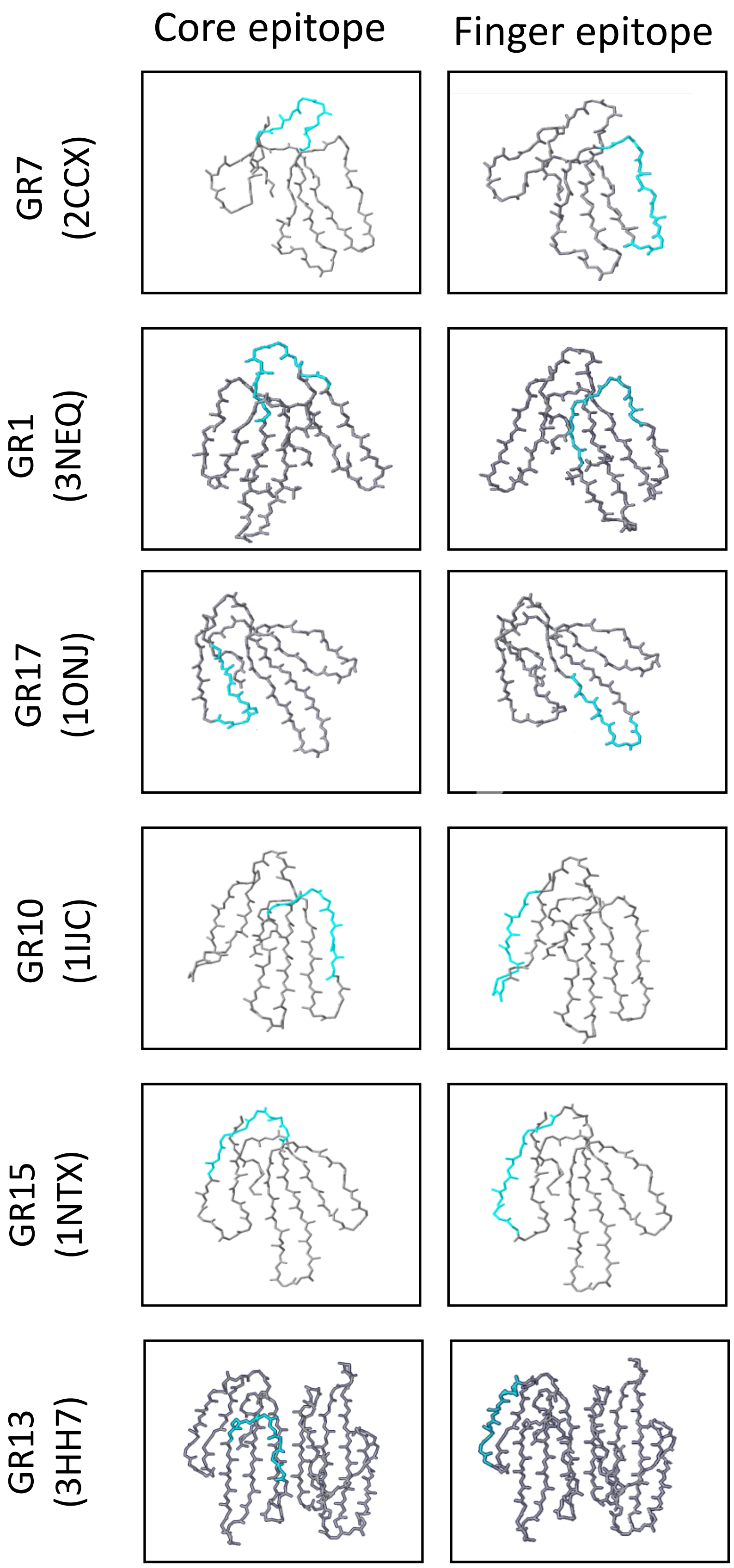

Figure S5. Structural models of 3FTxs detailing predicted core and finger epitope regions. Epitopes are highlighted blue. The Protein Data Bank numbers of structures epitopes were modelled on are indicated in brackets.

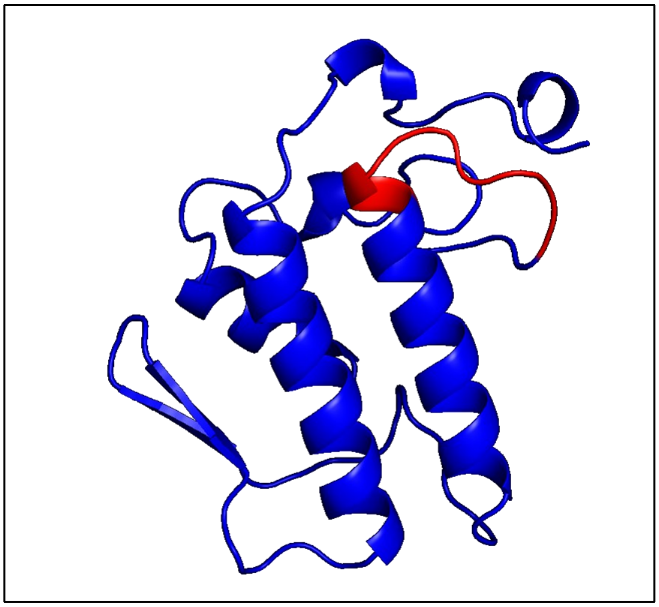

Figure S6. Structural modelling of group I PLA_2 epitopes. Epitope region for PLA2_1, PLA2_2 and PLA2_3 is shown in red. Epitopes were modelled on the structure of Protein Data Bank entry 1A3D, a PLA_2 from *N. naja* venom.

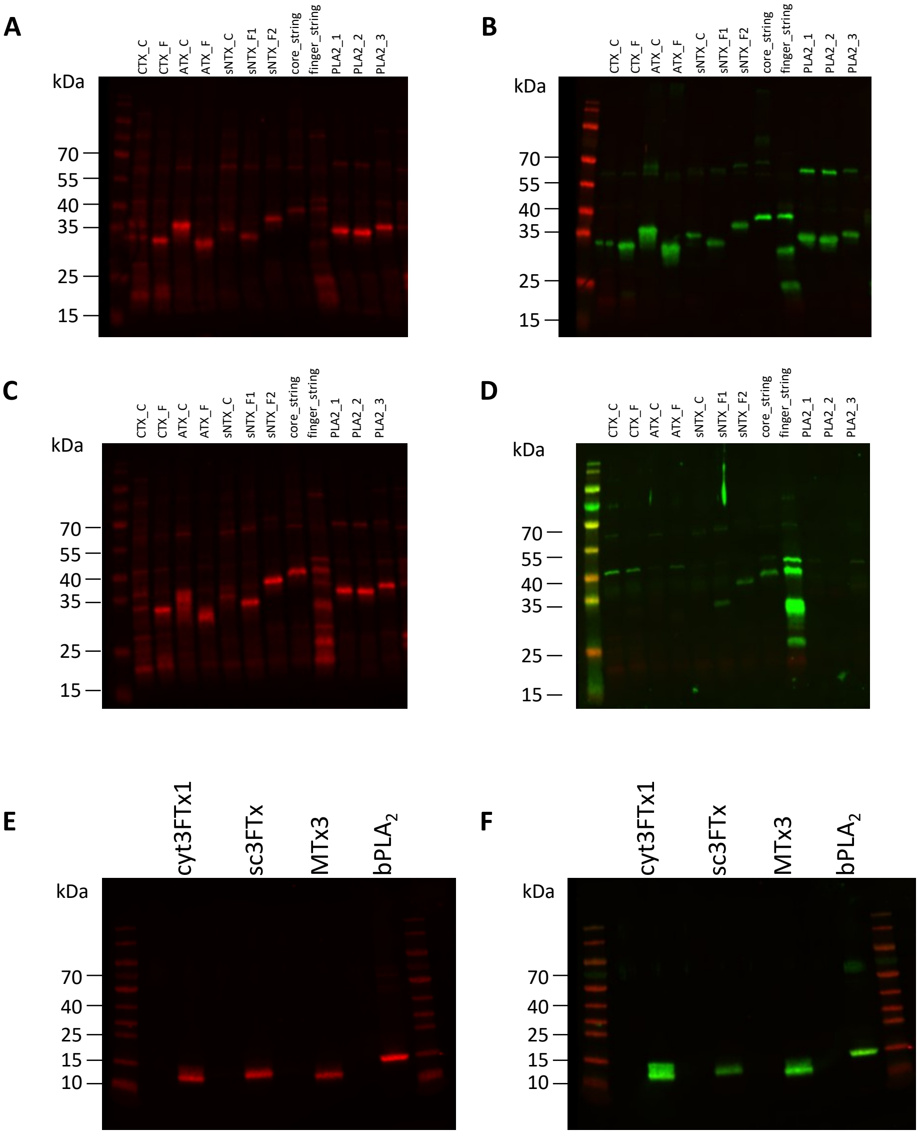

Figure S7. Antibody recognition of epitopes used for immunisation. Top panel (A and B) shows an anti-His Western blot to detect veVLP purified immunogens, where A = Total Protein stain (imaged in 700 nm channel) and B = protein bands detected by anti-His antibody (imaged in 800 nm channel). Middle panel (C and D) shows a Western blot using SAIMR Polyvalent antivenom as the primary antibody to detect veVLP purified immunogens, where C = Total protein stain (imaged in 700 nm channel) and D = protein bands detected by SAIMR Polyvalent antivenom (imaged in 800 nm channel). Bottom panel (E and F) shows a Western blot using SAIMR Polyvalent antivenom as the primary antibody to detect the purified whole toxins from which the epitopes were designed, where E = Total protein stain (imaged in 700 nm channel) and F = protein bands detected by SAIMR Polyvalent antivenom (imaged in 800 nm channel). Toxins used were: MTx3 - muscarinic toxin 3 from *D. angusticeps*, bought from Alomone Labs (Jerusalem, Israel), Cyt3FTx1 – cytotoxic 3FTx from *N. haje,* sc3FTx – short chain 3FTx from *N. haje* and bPLA2 – basic PLA_2 from *N. nigricollis*

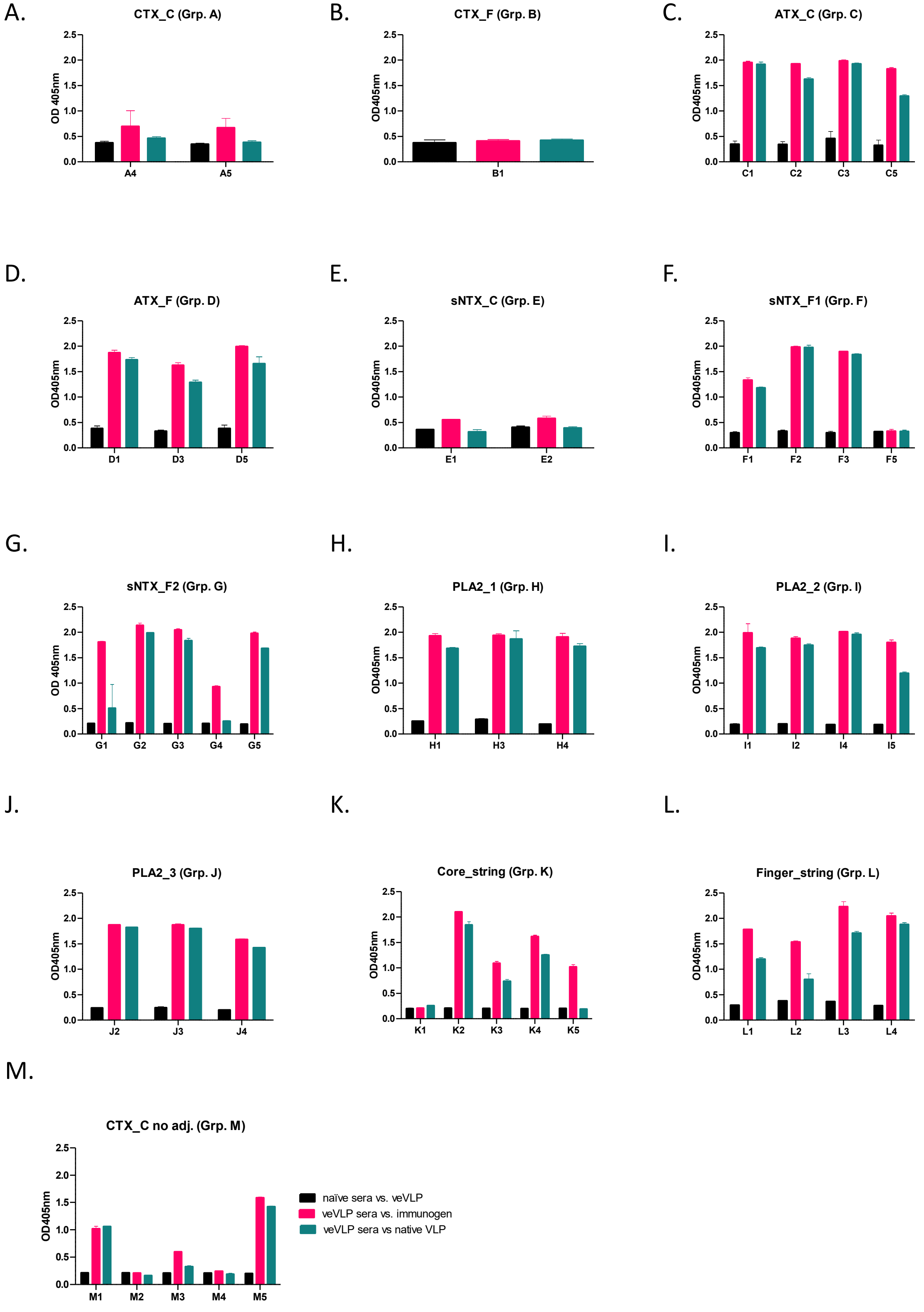

Fig. S8. ELISA results of naïve and individual terminal sera (week 14) at 1 in 500 dilutions vs respective veVLP. All results are of triplicate readings.

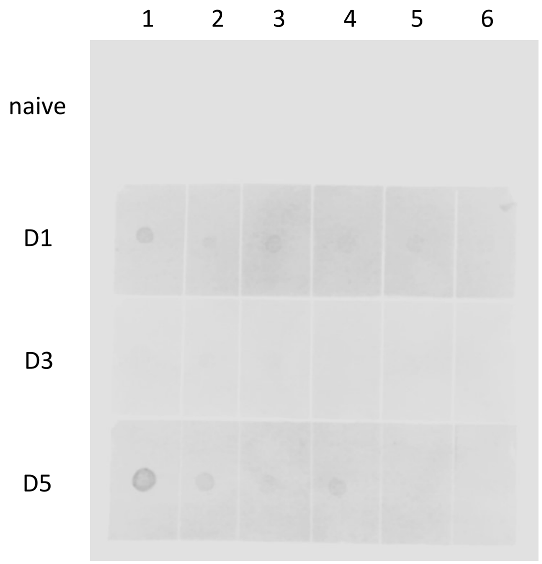

Figure S9. Dotblot of Group D (ATX_F) terminal sera. Dotblot of naïve sera (top) or individual terminal veVLP sera from animals in group D, all tested at 1 in 500 dilution. Venoms; 1 = *B. candidus,* 2 = *D. polylepis,* 3 = *N. kaouthia,* 4 = *N. subfulva,* 5 = *N. nigricollis,* 6 = *O. scutellatus.* Blots were imaged in the 800 nm channel for 2 minutes.
