# Supplementary material for: Virus-like particles displaying conserved toxin epitopes stimulate polyspecific, murine antibody responses capable of snake venom recognition
Source: Sci Rep. 2022 Jul 5;12:11328. doi: 10.1038/s41598-022-13376-x (PMC9256628; doi:10.1038/s41598-022-13376-x)
Supplement: Supplementary file 9 — Supplementary Information 9. [file 41598_2022_13376_MOESM9_ESM.docx]

**Supplemental Materials and Methods**

Epitope design

Conserved linear immunogenic epitopes of small molecular weight toxins found in sub-Saharan African and Asian elapid snakes were selected using in silico approaches. A database of full-length 3FTx amino acid (AA) sequences was compiled based on existing transcriptomic, proteomic and PDB available sequences (Supp. Table S1, Supp. File S1). The 264 3FTx sequences in this dataset, representing 27 species from three continents, were clustered into groups based on a low-resolution bootstrap consensus tree (500 replicates) generated using the neighbour joining method in MEGA 7^1^ (Supp. File S2). The evolutionary distances were computed using the number of differences method to display the number of amino acid differences per sequence. The analysis involved 268 amino acid sequences, the additional four sequences representing distantly-related outgroup candidates. All ambiguous positions were removed for each sequence pair, resulting in a total of 80 positions in the final dataset. Clades were then manually interrogated and assigned to homology groups based on sequence similarity determined using blastp^2^. A second dataset consisting of 156 Group I PLA_2_s (representing 17 species across Africa and Asia) were treated as a single homology group due to their high level of sequence conservation (Supp. File S3).

Groups were batch analysed for the presence of possible epitope regions using Bepipred 2.0 with epitope thresholds of 0.52-0.55^3^. This data was compared to sequence alignments and residue conservation analysis performed in MEGA 7. Sequences were shortlisted based on the presence of a predicted epitope region and high (≥80%) sequence conservation. For groups where this was not possible, sequences were shortlisted based on conservation alone. Finally, sequence accessibility, predicted using 3D modelling (PyMOL, Version 2.0, Schrödinger LLC), was used to finalise candidate epitope selection. For 3FTx, additional selection based on epitope location (either presence on the hydrophobic core and first and/or third finger) was employed^4^.

Recombinant antigen expression and purification

A plasmid to express HBcAg VLPs and to allow sub-cloning of epitopes into the major immunodominant region was generated that is hereafter referred to as pVLP. Briefly, primers p14F (AGCAGCACTAGTTGATCCGGCTGCTAACAAAGC) and p14R (AGCAGCGGTACCGGGAAACCGTTGTGGTCTC) were used to amplify the backbone of pDEST14 (Invitrogen) (from position 1915 to 48), removing the encoding Gateway cloning apparatus, but retaining the T7 promoter, whilst introducing 5’ SpeI and 3’ KpnI restriction sites. A DNA fragment based on Kratz et al 1999^5^ encoding an *Escherichia coli* codon optimised version of HBcAg, modified to encode an N-terminal 6 x HIS tag, thrombin cleavage site, and XhoI and Xbal flanking the immunodominant region, flanked by 5’ KpnI and SpeI restriction sites, was synthesised by MWG Eurofins (Germany). The subsequent synthesised HBcAg and amplified pDEST14 PCR product were digested with KpnI and SpeI, ligated (as below) prior to transformation into 5-alpha competent *E. coli* to produce pVLP (Supp. Fig S1) and then selected on Luria Broth (LB) plates with 100 µg/mL ampicillin.

DNAs encoding the identified epitopes (Table 1) were synthesised (Integrated DNA Technologies) as gene fragments with additional flanking GSG_3_ linker sequences (codon optimised for expression in *E. coli*) and 5’ XhoI and 3’ XbaI restriction sites. DNA encoding string epitopes were synthesised in the same manner, except epitopes were in sequential sequence separated by a double lysine linker^6^. Gene fragments and pVLP were digested with XhoI and XbaI, dephosphorylated using recombinant shrimp alkaline phosphatase, and purified using QIAquick PCR purification kit (Qiagen) according to manufacturer’s instructions. Gene fragments were ligated into pVLP using T4 DNA ligase and transformed into chemically competent *E. coli* BL21 (DE3). Positive clones were selected using LB plates with 100 µg/mL ampicillin. Single colonies were inoculated into 10 mL LB with ampicillin and incubated at 37 °C overnight with shaking at 220 rpm. The following day, cultures were diluted 1 in 100 into LB with ampicillin and incubated at 37 °C with shaking (220 rpm) until reaching an optical density (OD) at 600 nm of 0.5 - 0.6. Cultures were then induced with 400 µM IPTG and incubated at 30 °C for 4 hours with shaking at 220 rpm. Bacterial cultures were pelleted at 7650 x g for 8 minutes, with resulting pellets resuspended in 10 mL 50 mM Tris-Cl, 500 mM sodium chloride pH 7.0 with 1 X CellLytic B and frozen at -20 °C. Cell pellets were thawed and 100 µg lysozyme and 1 X mini-cOmplete EDTA-free Protease Inhibitor Tablets were added. The lysate was sonicated on ice for 3 x 20 seconds with 30 second pauses prior to addition of 5 µL benzonase.

Lysates were pelleted at 14,000 x g for 10 minutes and the supernatant was diluted 1:1 (v/v) in 50 mM Tris-Cl, 500 mM sodium chloride, 20 mM imidazole pH 7.0. Ni-NTA agarose was poured into a gravity flow column with a final column volume (CV) of 0.5 mL and equilibrated with 10 CV of 50 mM Tris-Cl, 500 mM sodium chloride, 10 mM imidazole pH 7.0. Lysate was then poured onto the nickel column and left to bind for 30 minutes at room temperature. Lysate was run through the column, then the column was washed with 10 CV of 50 mM Tris-Cl, 500 mM sodium chloride, 50 mM imidazole pH 7.0. An elution buffer of 50 mM Tris-Cl, 500 mM sodium chloride, 500 mM imidazole pH 7.0 was added to the column (5 CVs) and incubated for 10 minutes. Eluted protein was collected and immediately desalted into PBS pH 7.4 using a PD10 desalting column (GE Healthcare) according to manufacturer’s instructions. Protein was subsequently concentrated with a 100 kDa molecular weight cut off centrifugal filter which additionally reduced the presence any monomers and ensured that mainly assembled VLPs (~30 nm^7,8^) were retained. Protein concentration was estimated via absorbance measurements at A280 nm (NanoDrop, ThermoScientific) and protein purity was assessed using SDS-PAGE with Coomassie staining, and anti-His immunoblots (see below). Protein was diluted in PBS to 0.05 µg/µL in a total volume of 160 µL, flash frozen and stored at -80 °C until immunisation.

Purified toxins

Muscarinic toxin 3 from *D. angusticeps* was purchased from Alomone labs (Israel). The 3FTx cytotoxin 1 and group I basic PLA_2_ were purified from *N. nigricollis* (Tanzania) venom, and the 3FTx sNTx was purified from *N. haje* (Uganda) venom using the following chromatographic methods. In both cases, resuspended whole venom was initially fractionated by gel filtration chromatography on a column of Superdex 200HR (Cytiva). The buffer used was PBS (25 mM sodium phosphate, 0.15M NaCl, pH 7.2). This separated the 3FTx (*N. haje*) or 3FTx/PLA_2_ (*N. nigricollis*) venom components from the higher molecular weight venom proteins (e.g., snake venom metalloproteinases [SVMP], L-amino acid oxidases and cysteine-rich secretory proteins). The 3FTx/PLA_2_ proteins eluted as a large peak at the expected molecular weight (8-15 kDa). The protein in these peaks was dialysed against 50 mM sodium phosphate, pH 6.0 and subjected to a second chromatography step on a cation exchange column (4.7 mL HiScreen SP HP, Cytiva).

For *N. nigricollis*, cation exchange chromatography separated the various 3FTx/PLA_2_ forms into multiple peaks. Cytotoxin 1 eluted as a single dominant peak at around 0.28 M NaCl. This was fully purified using a third chromatography step on a Phenyl Superose hydrophobic interaction column. The protein was loaded in 25 mm sodium phosphate, 1.5 M NaCl, pH 7.2 and eluted with a gradient of decreasing NaCl concentration. The pure protein was seen as a single 9 kDa band on SDS-PAGE and through mass spectrometric analysis it was found to have an intact mass of 6817 Da (monoisotopic), matching that of cytotoxin 1 (P01476) of *N. mossambica*. Basic PLA_2_ eluted from the cation exchange column as the last major peak (Ca. 0.45 M NaC). This was further purified using hydroxyapatite chromatography (CHTI matrix, BioRad). Thus, after dialysing into 5 mM sodium phosphate, pH 6.8, the protein was loaded onto the CHTI column and then eluted with an increasing concentration of sodium phosphate (up to 500 mM). The pure protein was observed as a single band on SDS-PAGE at 14 kDa and was found to have an intact mass of 13,249 Da (monoisotopic).

Cation exchange chromatography of the *N. haje* 3FTx material resulted in one major peak (Ca. 80% of the venom protein) eluting at Ca. 0.2 M NaCl and one later-eluting smaller peak. The major peak was subjected to a third chromatography step using reverse-phase on a Source 15RPC ST column (Cytiva) and using a gradient of acetonitrile in 0.1% TFA for the separation. This resulted in the elution of two equal-sized peaks, the second containing a protein with mass 6848 Da corresponding to the short neurotoxin 1 [Toxin CM-6, P68418] of *N. haje*.

Quantitative Western blots

For quantitative western blots, the appropriate amount of venom to load per lane (based on dry weight determination) was first determined to ensure the total protein was within the linear range for normalisation, as recommended by LI-COR Biosciences. Serial dilutions of each venom were run on SDS-PAGE gel and transferred to nitrocellulose membrane followed by Total Protein 700 staining, as described above. Blots were imaged for 2 minutes in the 700 nm channel on an Odyssey Fc Imaging System, and the fluorescence intensity in the whole lane for each venom dilution was recorded. A graph of venom dilution and fluorescence intensity was plotted to determine the linear range. For all venoms used in this study, 2 µg was within the linear range and thus was chosen for all western blots. For quantification of the fluorescence intensity in the 800 nm channel, all blots were normalised as per LI-COR Biosciences recommendations. Briefly, the total 700 nm (total protein) signals for each venom were recorded, and the normalisation ratio was determined by dividing the signal for the lane by the highest signal recorded for that venom. The normalisation ratio was then applied to the 800 nm fluorescence signal to determine normalised fluorescence intensity values.

Dotblots

Dotblotting experiments were performed as described above for western blotting, except that 2 µg venom was prepared in PBS and pipetted directly onto 0.2 µm nitrocellulose membrane and allowed to air dry. Thereafter, all subsequent steps were performed as outlined above.

References

1. Kumar, S., Stecher, G. & Tamura, K. MEGA7: Molecular Evolutionary Genetics Analysis Version 7.0 for Bigger Datasets. *Mol. Biol. Evol.* **33**, (2016).

2. Altschul, S. F., Gish, W., Miller, W., Myers, E. W. & Lipman, D. J. Basic local alignment search tool. *J. Mol. Biol.* **215**, (1990).

3. Jespersen, M. C., Peters, B., Nielsen, M. & Marcatili, P. BepiPred-2.0: Improving sequence-based B-cell epitope prediction using conformational epitopes. *Nucleic Acids Res.* **45**, (2017).

4. Rajagopalan, N., Manjunatha Kini, R., Doley, R. & Hegde, R. Snake Venom Three-Finger Toxins. in *Handbook of Venoms and Toxins of Reptiles* (2009). doi:10.1201/9781420008661.sec3.

5. Kratz, P. a, Böttcher, B. & Nassal, M. Native display of complete foreign protein domains on the surface of hepatitis B virus capsids. *Proc. Natl. Acad. Sci. U. S. A.* **96**, 1915–1920 (1999).

6. Wagstaff, S. C., Laing, G. D., Theakston, R. D. G., Papaspyridis, C. & Harrison, R. A. Bioinformatics and multiepitope DNA immunization to design rational snake antivenom. *PLoS Med.* **3**, 0832–0844 (2006).

7. Roose, K., Baets, S. De, Schepens, B. & Saelens, X. Hepatitis B core–based virus–like particles to present heterologous epitopes. *Expert Rev. Vaccines* **12**, 183–198 (2013).

8. Bruun, T. U. J., Andersson, A. M. C., Draper, S. J. & Howarth, M. Engineering a Rugged Nanoscaffold to Enhance Plug-and-Display Vaccination. *ACS Nano* **12**, (2018).
